# Synthesis, Molecular Docking Analysis, and Biological Evaluations of Saccharide-Modified Sulfonamides as Carbonic Anhydrase IX Inhibitors

**DOI:** 10.3390/ijms222413610

**Published:** 2021-12-19

**Authors:** Zuopeng Zhang, Huali Yang, Ye Zhong, Yueqing Wang, Jian Wang, Maosheng Cheng, Yang Liu

**Affiliations:** Key Laboratory of Structure-Based Drug Design & Discovery of Ministry of Education, School of Pharmaceutical Engineering, Shenyang Pharmaceutical University, Shenyang 110016, China; zzp_92@163.com (Z.Z.); zy_mc666@163.com (Y.Z.); wangyueqingpoem@163.com (Y.W.); wjmed@126.com (J.W.); mscheng@syphu.edu.cn (M.C.)

**Keywords:** saccharide-modified sulfonamides, CA IX, cell viability, migration, molecular docking

## Abstract

Based on the strategy of the “tail approach”, 15 novel saccharide-modified sulfonamides were designed and synthesised. The novel compounds were evaluated as inhibitors of three human carbonic anhydrase (CA) isoforms, namely cytoplasmic CA II, transmembrane CA IX, and XII. Most of these compounds showed good activity against CAs and high topological polar surface area (TPSA) values, which had a positive effect on the selective inhibition of transmembrane isoforms CA IX and XII. In the in vitro activity studies, compounds **16a**, **16b**, and **16e** reduced the viability of HT-29 and MDA-MB-231 cells with a high expression of CA IX under hypoxia. The inhibitory activity of compound **16e** on the human osteosarcoma cell line MG-63 with a high expression of CA IX and XII was better than that of AZM. Moreover, high concentrations of compounds **16a** and **16b** reversed the acidification of the tumour microenvironment. In addition, compound **16a** had a certain inhibitory effect on the migration of MDA-MB-231 cells. All the above results indicate that the saccharide-modified sulfonamide has further research value for the development of CA IX inhibitors.

## 1. Introduction

Hypoxia and acidification are salient features of many tumour tissues [1]. Such a microenvironment is of great significance to tumour cell survival, proliferation, invasion, and the development of drug resistance [1,2,3]. Hypoxia in the microenvironment is caused by an imbalance between the rapid growth of tumour cells and the oxygen supply capacity, while acidification is caused by a variety of proteins including carbonic anhydrase (CAs, EC 4.2.1.1) [1,4,5]. In the CA family, CA IX and XII are isoforms related to acidification of the tumour microenvironment [6]. CA IX is not significantly expressed in most normal tissues but is overexpressed in many aggressive tumours including lung, breast, colorectal, head and neck, lung, and kidney cancers. CA IX is of great significance for maintaining the tumour microenvironment and is closely related to the proliferation, migration, and invasion of tumour cells [7,8]. Therefore, CA IX has been validated as a promising target for cancer diagnostics and treatments. Compared with CA IX, CA XII is distributed in a variety of normal tissues. CA XII is minimally expressed in various normal tissues (including in the kidney, colon, prostate, pancreas, ovary, testis, lung, and brain), but its expression is upregulated in cancer cells derived from these tissues [9]. Several recent studies have shown that CA XII is an attractive and potential target for tumour treatment and diagnostic intervention, especially for hypoxic tumours that are resistant to traditional radiotherapy and chemotherapy [9,10,11]. Both CA IX and CA XII play an important role in maintaining the acidic microenvironment of tumours in a variety of tumour tissues and have been proven to be vital to tumour growth and survival [12,13].

To date, there is no small molecule selective inhibitor of CA IX/XII on the market. Only one small molecule drug, SLC-0111, which targets CA IX/XII, is in clinical phase I/II [14]. The main challenge in the development of carbonic anhydrase inhibitors (CAIs) for antitumour therapy is the lack of isoform selectivity. Due to the wide distribution of CAs, CAIs without selectivity will lead to serious side effects [15,16]. However, the 12 catalytically active human CAs show high homology in their active sites, which makes the study of selective CAIs complicated and unsatisfactory [17,18]. To solve this problem, the “tail approach” is widely used, which splices fragments with different polarities and sizes to the tail of the pharmacophore to selectively inhibit extracellular isoforms [19,20]. Widely used and effective tails include bulky groups, fluorophores, cationic groups, and saccharides [21]. Among them, saccharides are favoured because of their wide sources, high biocompatibility, and high polarity.

Here, to investigate the application of saccharide modification in CA IX/XII inhibitors, we designed and synthesised 15 novel compounds by modifying sulfonamide with glucose. After performing relevant isoenzyme (including, tumour-associated isoforms *h*CA IX and XII and off-target isoform *h*CA II) tests on these compounds, molecular docking was carried out to provide insight into the mechanism of action of the compounds. The biological evaluation at the cellular level included the effects on tumour cell viability, extracellular pH changes, and cell migration.

## 2. Results and Discussion

### 2.1. Chemistry

**Scheme 1:**
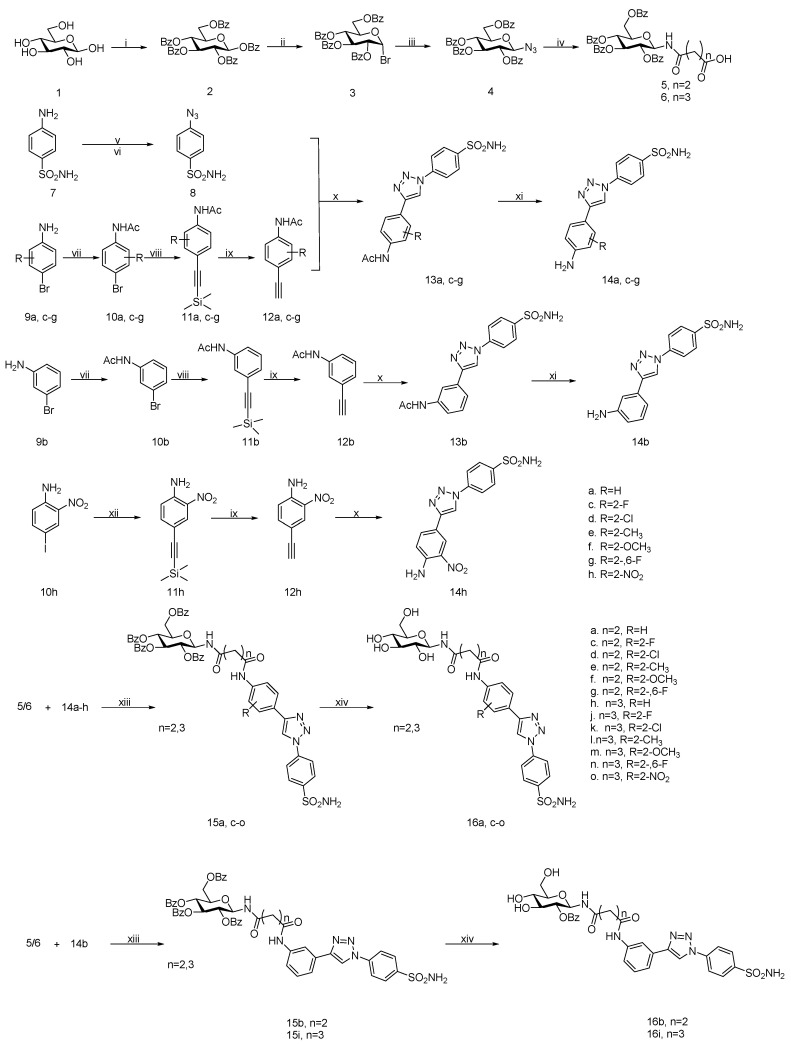
Synthesis of target compounds. Reagents and conditions: (**i**) BzCl, DMAP, Py, r.t.; (**ii**) HBr-HOAc, DCM, 0 °C-r.t.; (**iii**) NaN_3_, DMF, 50 °C; (**iv**) Pd/C, H_2_, succinic anhydride/glutaric anhydride, r.t.; (**v**) NaNO_2_, HCl, H_2_O, 0 °C; (**vi**) NaN_3_, H_2_O, 0 °C-r.t.; (**vii**) Ac_2_O, EtOAc, r.t.; (**viii**) Trimethylsilylacetylene, CuI, Pd(PPh_3_)_2_Cl_2_, TEA, DMF, Ar, 80 °C; (**ix**) TBAF, THF, r.t.; (**x**) CuSO_4_, sodium ascorbate, t-BuOH-H_2_O, 60 °C; (**xi**) NaOH, H_2_O, reflux; (**xii**) Trimethylsilylacetylene, Pd(PPh_3_)_2_Cl_2_, CuI, DIEA, THF, Ar, r.t.; (**xiii**) EDCI, Py, r.t.; (**xiv**) MeONa, MeOH, r.t.

The synthesis of novel derivatives of benzenesulfonamides bearing carbohydrate Groups **16a–o** is outlined in Scheme 1. This series of compounds adopted a convergent synthesis route, including two parts. One of the parts was the synthesis of the glycosyl motif. We selected *β*-D-glucose as the starting material to obtain benzoyl-protected intermediate **2** by reaction with benzoyl chloride. Then, through the reaction of **2** with HBr-HOAc, we obtained bromo-sugar (intermediate **3**) in good yield and the reaction of **3** and sodium azide in DMF produced azide-sugar (intermediate **4**). Finally, azide reduction and single-chain segmentation were completed in a one-pot reaction to obtain amino-sugar carboxylic acid derivatives (intermediates **5** and **6**). Thus far, we have completed part of the glycosyl motif.

The other part of the route was to build the fragment in the active site. 4-Azidobenzenesulfonamide (intermediate **8**) was obtained from sulfanilamide **7** treated with NaNO_2_ and NaN_3_ in acidic aqueous media. Moreover, *N*-(4-((trimethylsilyl)ethynyl)phenyl)acetamides (intermediates **11**) were obtained via Sonogashira coupling with various commercially available *m*- and *p*-bromoaniline derivatives **9** [22]. Intermediate **11** was deprotected with TBAF to obtain *N*-(4-ethynylphenyl)acetamide (intermediate **12**). Next, by reacting **8** and **12** through copper-catalysed azide-alkyne cycloadditions (CuAAC) [23], we obtained triazole derivatives (intermediates **13**). Then, deacetylated triazole derivatives (intermediates **14**) were prepared by refluxing intermediates **13** in a basic aqueous solution. To date, we have completed part of the fragment within the activity site.

The last was the splicing of the two parts. With the participation of EDCI, deprotected intermediates **14a–h** and **5**/**6** afforded glycosyl derivatives (intermediates **15a–o**), which were subsequently treated with MeONa/MeOH to furnish target compounds **16a–o**. ^1^H NMR, ^13^C-NMR, and HRMS techniques were used for the characterisation of the newly synthesised compounds.

### 2.2. hCAs Inhibitory Activity

All the synthesised compounds and key intermediates were assayed as inhibitors of two physiologically relevant CA isoforms, namely transmembrane tumour-associated *h*CA IX and off-target cytosolic *h*CA II, and some of the compounds with significant activity were supplemented with the *h*CA XII inhibitory activity test. The test used a method of monitoring the hydrolysis of 4-nitrophenylacetate (4-NPA.) The control compound was acetazolamide (AZM), which is clinically used as a carbonic anhydrase inhibitor. The in vitro enzyme inhibition test results of sulfatriazole derivatives **14a–h** and carbohydrate-based compounds **16a–o** are listed in Table 1.

Based on the data in Table 1, the structure–activity relationship (SAR) is summarised as follows:

(i). The inhibitory activities of sulfatriazole derivatives **14a–h** against *h*CA II and IX were 12.3–102.0 and 40.4–120.2 nM, respectively. Among compounds **14a–h**, **14a–c** showed comparable inhibitory activities to *h*CAII and IX as AZM, and the inhibitory activities of other compounds against *h*CAII and IX were weaker. The SAR of compounds **14a–h** for *h*CA II and IX was as follows: when the aromatic amine ring was unsubstituted or substituted by a fluorine atom with a small atomic volume, the inhibitory activities for *h*CAII and IX were good, but when the volume of substituents on aniline increased, both the electron-donating and electron-withdrawing groups caused the inhibitory activity against *h*CA II to decrease.

(ii). The inhibitory activities of saccharide-modified compounds **16a–o** against *h*CA II and IX were 10.1–177.2 and 51.6–543.9 nM, respectively. Compared with compounds **14a–h** without saccharide, the inhibitory activities of compounds **16a–o** on *h*CA II did not change significantly, but the activity of *h*CA IX decreased noticeably. At the same time, although the introduction of saccharide fragments did not significantly improve the inhibitory activities on the enzyme, the TPSA of the saccharide derivatives increased significantly, which reduced the membrane permeability of compounds, and provided the possibility of isoform selectivity based on subcellular localisation. 

(iii). Compounds **16a–b** and **16h–i** with unsubstituted benzene rings showed the best inhibitory activity against *h*CA II, ranging from 10.1 to 27.7 nM. When the benzene ring was substituted by halogen (F or Cl) and electron-withdrawing substituents (nitro or difluoro), the activity against *h*CA II decreased. When the benzene ring was substituted by electron-donating substituents, methyl substitution led to a slight decrease or maintenance of the inhibitory activity against *h*CA II compared with unsubstituted compounds, while methoxy caused a significant decrease. The effect of aliphatic chain length on *h*CA II inhibitory activities was not obvious.

(iv). The inhibitory effect of compounds **16a–o** on *h*CA IX was not as clear as that on *h*CA II. The compounds with the best inhibitory activity against *h*CA IX were **16a**, **16b**, **16e** and **16m**, ranging from 51.6 to 99.6 nM. When the aliphatic chain was succinamide (n = 2), the most active compound was **16a**, and replacement with meta-aniline or substitution with different groups both caused a decrease in activity. The decrease in activity caused by difluoro substitution was the most significant, resulting in a 10-fold decrease in activity. When the aliphatic chain was glutamide (n = 3), the most active compound was **16m**. Compared with succinamide, the methoxy group played a positive role in increasing the activity.

(v). Compounds **16a**, **16b**, **16e**, and **16m** were selected to test the inhibitory activity against *h*CA XII, and the values ranged from 135.3 to 255.9 nM. Their inhibitory effect on *h*CA XII was weaker than that on *h*CA II and IX.

### 2.3. Docking Studies

**Figure 1:**
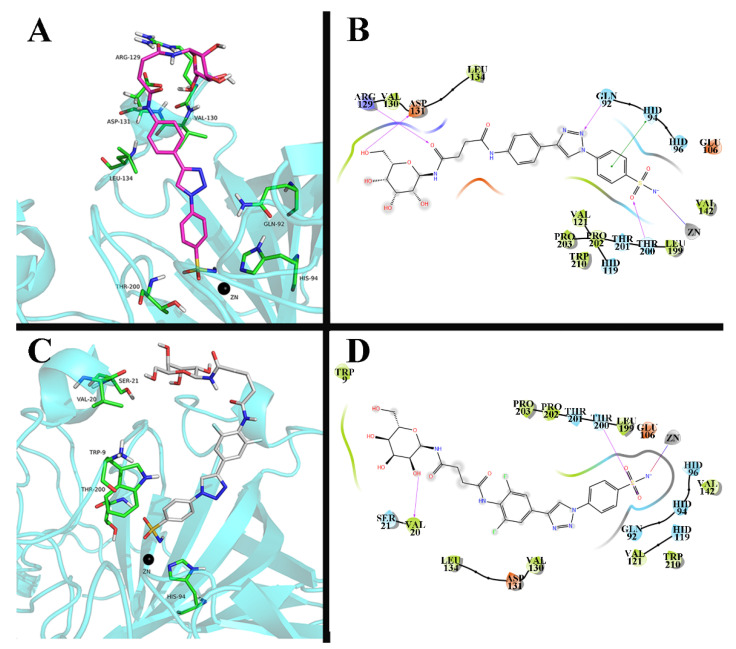
The modes of binding for compounds in *h*CA IX. (**A**,**B**). Compound **16a** (pink). (**C**,**D**). Compound **16g** (gray).

We re-docked the native ligand (9FK) into CA IX (PDB ID: 5FL4) with optimised docking parameters and successfully produced the binding modes observed in the crystal structures. The ligand formed hydrogen bonding with Gln92, His119, and Thr200 in the active site, and hydrophobic interaction with His94, His96, Val130, and Leu134. The RMSD values of docking and experimental posture is 0.699 Å (detailed data is provided in the Table S1 and Figure S1 of supplementary information). Then, we conducted docking studies on the saccharide-modified compounds **16a** and **16g** to explain why they exhibit different inhibitory activities. The results are shown in Figure 1.

As shown in Figure 1, the modes of action of compounds **16a** and **16g** at the active site were quite different. Compound **16a** formed strong interactions with Gln92, His94, and Thr200 at the active site, while **16g** only interacted with Thr200 through the sulfonamide group. In addition, the mode of binding of the two glycosyl fragments at the entrance of the active site was different. Compound **16a** formed hydrogen bonds with Arg129 and Asp131 through the glycosyl hydroxyl group and the amide in the structure, while the glycosyl in **16g** only had a hydrogen bond with Val20. Moreover, the docking scores of **16a** and **16g** were −8.37 kcal/mol and −6.49 kcal/mol, respectively. From the docking scores, it was apparent that compound **16a** was better than **16g**. The fewer interactions and lower binding stability might be responsible for the lower activity of **16g**.

### 2.4. In Vitro Cytotoxicity Studies on Cancer Cells

The CCK-8 assay was used to evaluate the cytotoxicity of compounds **16a**, **16b**, and **16e** on the breast cancer cell line MDA-MB-231 and colon cancer cell line HT-29. MDA-MB-231 and HT-29 are tumour cells that have been reported to express CA IX. We cultivated them under normoxia and hypoxia respectively and then evaluated the effects of the compounds on tumour cell viability at different concentrations. The clinical drug AZM was selected as the positive cytotoxic reference compound at the same concentrations (detailed data is provided in the Tables S2 and S3 of supplementary information).

**Figure 2:**
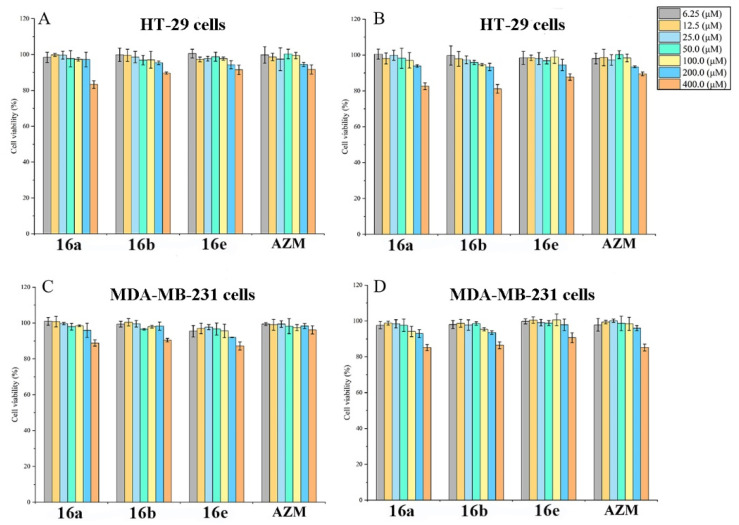
Effects of compounds **16a**, **16b**, **16e**, and AZM on the cell viability of HT-29 and MDA-MB-231 cells under normoxic (**A**,**C**) and hypoxic (**B**,**D**) conditions.

According to the results of the cell viability test, we summarised the following conclusions:

(i). Data from Figure 2 show that both AZM and the tested compounds had a very limited effect on the growth of the two tumour cell lines under normoxic conditions. When the cell lines were cultured under hypoxia, the inhibitory effect of the compounds was improved expect for that of 16e; the inhibitory effect of **16e** on MDA-MB-231 under hypoxia is slightly weaker than that under normoxic condition. 

(ii). The results showed that HT-29 colon cancer cells were more susceptible to inhibition by the tested compounds, and a 20% decrease in cell viability was observed in compounds **16a** and **16b** at the highest concentration of 400 μM.

(iii). Compared to AZM, compounds **16a**, **16b**, and **16e** exhibited higher inhibitory activity against HT-29 and MDA-MB-231 cells, which was particularly evident in the hypoxic HT-29 cell line.

**Figure 3:**
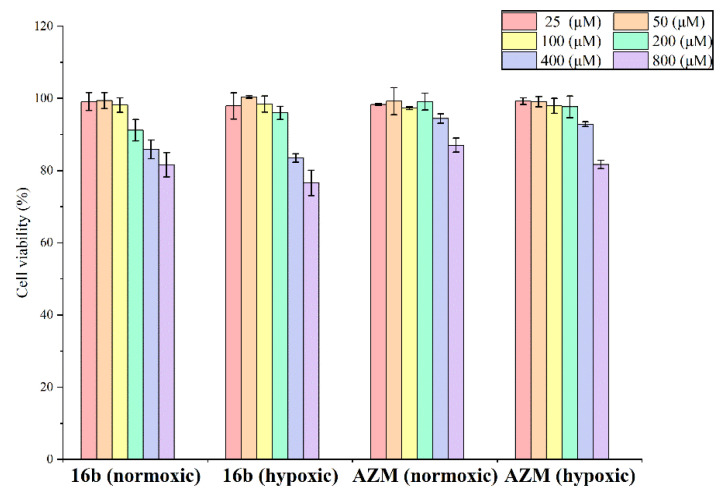
Effects of compounds **16b** and AZM on the cell viability of MG-63 cell under normoxic and hypoxic conditions.

The human osteosarcoma cell line MG-63 is a high-expressing cell line of CA IX and XII [24]. We selected compound **16b** with the best activity in the above studies to study the cell viability inhibitory activity of MG-63 (detailed data is provided in the Table S4 of supplementary information). The results are shown in Figure 3.

The test results showed that the inhibition of MG-63 by **16b** was concentration-dependent, and its inhibitory activity was better than that of the positive control AZM. Moreover, under a hypoxic environment, the inhibitory effect of **16b** on MG-63 cells was slightly higher than that of normoxia.

### 2.5. Measurement of Extracellular pH

**Figure 4:**
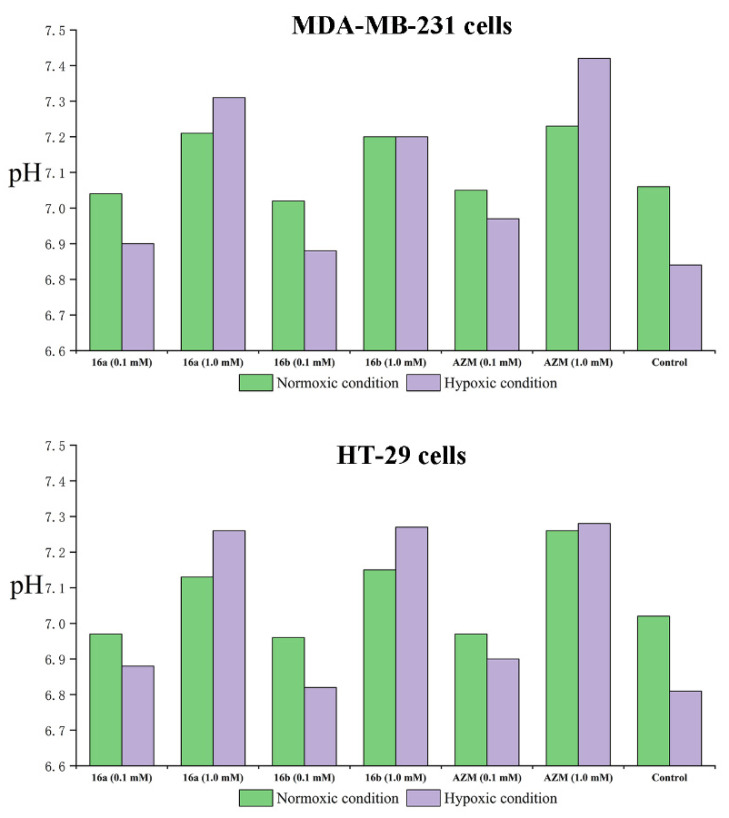
Extracellular pH measurement of MDA-MB-231 and HT-29 cells under normoxic and hypoxic conditions.

The main role of CA IX and XII in tumour development is to regulate the pH of the tumour growth microenvironment so that tumour cells are in a weakly acidic environment. Such an acidic environment is conducive to the growth of tumour cells and protects tumour cells from being killed by chemotherapeutics. We evaluated the effects of 0.1 mM and 1.0 mM of compounds **16a** and **16b** on the pH of the tumour cell growth environment under normoxia or hypoxia (detailed data is provided in the Tables S5 and S6 of supplementary information). The results are shown in Figure 4.

The results showed that both **16a** and **16b** could improve the extracellular pH of MDA-MB-231 and HT-29 cells at concentrations of 0.1 mM and 1.0 mM, and the effect was concentration-dependent. The pH change of the extracellular space was especially distinct in hypoxic culture. After the tumour cells were cultured with the 1.0 mM compounds, the extracellular pH increased significantly so that the environment changed from acidic to alkaline.

### 2.6. Cell Migration Assay

**Figure 5:**
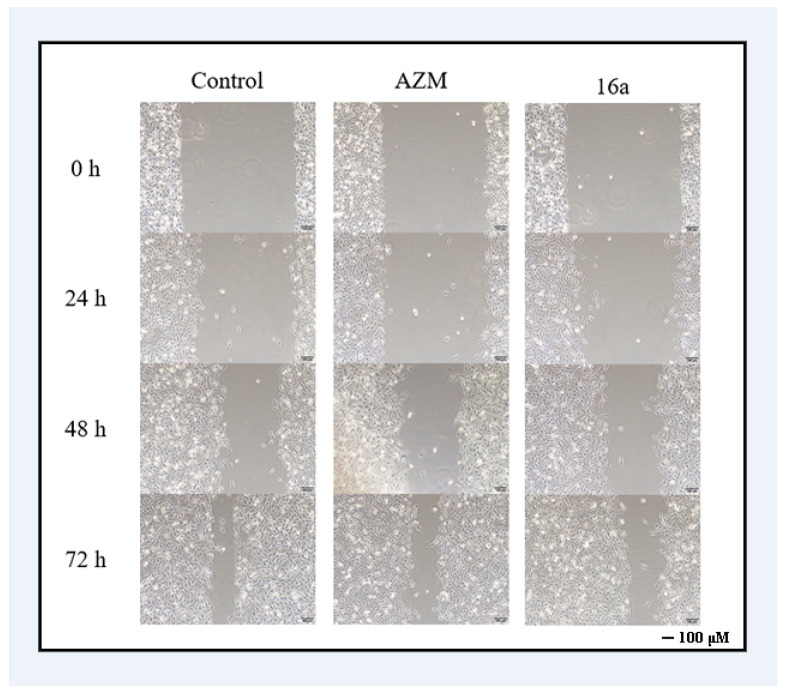
The inhibitory effect of **16a** at a concentration of 400 μM on the migration of MDA-MB-231 cell in vitro.

Tumour cell migration is one of the basic biological characteristics of tumour cells. We carried out a study on the antitumour migration activity of compound **16a**, which showed high CA IX inhibitory activity, to investigate the effect of a CA IX inhibitor on tumour cell migration. MDA-MB-231 was selected as the experimental cell line to explore the ability of **16a** to reduce tumour cells migration under hypoxia. As shown in Figure 5, after the positive control AZM and **16a** at a concentration of 400 μM were incubated with MDA-MB-231 hypoxia for 72 h. Compared with the blank group, **16a** had a slight inhibitory effect on tumour cell migration in the first 24 h, and this inhibitory effect could hardly be detected at 72 h. Compared with AZM, **16a** improved the inhibition of tumour cell migration, but it was not obvious.

## 3. Materials and Methods

### 3.1. Chemistry

All the reagents were obtained from commercial sources and used without further purification unless otherwise specified. Solvents were dried and redistilled before use in the usual manner. All chemicals and solvents were analytical grades. Analytical TLC was performed using silica gel HF_254_ (Qingdao Haiyang Chemical, Qingdao, China). Preparative column chromatography was performed with silica gel H. Melting points were obtained on a Büchi melting point B-540 apparatus (Büchi Labortechnik, Flawil, Switzerland). ^1^H and ^13^C NMR spectra (details of raw data for compounds, see Figures S2–S83) were recorded on Bruker ARX 600 MHz or 400 MHz spectrometers (Bruker, Zurich, Switzerland). HRMS was obtained on an Agilent ESI-QTOF instrument (Agilent, Santa Clara, CA, USA).

#### 3.1.1. Synthesis of Penta-O-Benzoyl-β-D-Glucopyranose (**2**) and 2,3,4,5-Tetra-O-Benzoyl-α-D-Glucopyranosyl Bromide (**3**)

Intermediates **2** and **3** were synthesised as reported previously [25]: yield (92% and 88%); white powder; m.p. 129–131 °C and 174–175 °C, respectively.

#### 3.1.2. Synthesis of 2,3,4,6-Tetra-O-Benzoyl-β-D-Glucopyranosyl Azide (**4**)

To a solution of intermediate **3** (6.6 g, 10.0 mmol) in anhydrous DMF (75 mL), NaN_3_ (5.2 g, 80.0 mmol) was added. The mixture weas heated at 50 °C under anhydrous condition followed by constant stirring overnight. After the reaction completion (monitored by TLC), the mixture was diluted with DCM (150 mL), washed with H_2_O (200 mL) and brine (100 mL), and dried over Na_2_SO_4_. After the filtration and evaporation of DCM, the product was purified chromatographically (PE:EA = 4:1) to provide intermediate **4**. White solid (4.5 g, 73% yield). ^1^H-NMR (DMSO-*d_6_*, 600 MHz) *δ* 8.04–7.97 (m, 2H), 7.90 (d, *J* = 8.3 Hz, 2H), 7.85 (d, *J* = 7.5 Hz, 2H), 7.74 (d, *J* = 8.3 Hz, 2H), 7.65 (ddd, *J* = 21.3, 14.9, 7.8 Hz, 3H), 7.57–7.50 (m, 5H), 7.46 (t, *J* = 7.3 Hz, 2H), 7.40 (t, *J* = 7.0 Hz, 2H), 6.06 (td, *J* = 9.4, 2.3 Hz, 1H), 5.74 (td, *J* = 9.8, 2.2 Hz, 1H), 5.57–5.51 (m, 1H), 5.47 (td, *J* = 9.2, 2.5 Hz, 1H), 4.68–4.61 (m, 1H), 4.59 (d, *J* = 12.4 Hz, 1H), 4.53 (dd, J = 12.4, 4.4 Hz, 1H) ppm. ^13^C-NMR (DMSO-*d_6_*, 151 MHz) δ 165.84, 165.57, 165.07, 134.53, 134.31, 134.00, 129.75, 129.46, 129.25, 128.94, 128.75, 87.10, 73.40, 71.73, 69.23, 62.93, 40.53 ppm.

#### 3.1.3. General Procedure of Synthesis of [(2,3,4,6-Tetra-O-Benzoyl-β-D-Glucopyranosyl)amino] acid Derivatives (**5**) and (**6**)

Intermediates **5** and 6 were synthesised as reported previously [26] Intermediate **5**, m.p. 88–90 °C. ^1^H-NMR (DMSO-*d_6_*, 600 MHz) *δ* 12.12 (s, 1H), 8.93 (d, *J* = 9.5 Hz, 1H), 7.97 (d, *J* = 7.2 Hz, 2H), 7.84 (d, *J* = 7.3 Hz, 2H), 7.79 (d, *J* = 7.5 Hz, 2H), 7.72 (d, *J* = 7.4 Hz, 2H), 7.69 (t, *J* = 7.2 Hz, 1H), 7.61 (dd, *J* = 16.8, 4.1 Hz, 2H), 7.55 (q, *J* = 7.3 Hz, 3H), 7.46 (dd, *J* = 9.4, 6.1 Hz, 4H), 7.41 (t, *J* = 7.7 Hz, 2H), 6.06 (t, *J* = 10.1 Hz, 1H), 5.81 (t, *J* = 9.3 Hz, 1H), 5.60 (t, *J* = 9.7 Hz, 1H), 5.35 (t, *J* = 9.4 Hz, 1H), 4.61 (d, *J* = 9.8 Hz, 1H), 4.52 (d, *J* = 11.1 Hz, 1H), 4.46 (dd, *J* = 12.4, 3.5 Hz, 1H), 2.29 (tdd, *J* = 23.4, 20.7, 7.6 Hz, 4H) ppm. ^13^C-NMR (DMSO-*d_6_*, 151 MHz) *δ* 173.92, 173.08, 172.26, 165.80, 165.54, 165.25, 134.21, 133.99, 129.64, 129.25, 129.03, 77.75, 74.40, 72.73, 72.16, 69.39, 62.92, 30.47, 29.14 ppm. 

Intermediate **6**, m.p. 94-96 °C. ^1^H-NMR (DMSO-*d_6_*, 600 MHz) *δ* 12.03 (s, 2H), 8.87 (d, *J* = 9.5 Hz, 1H), 7.97 (dd, *J* = 8.2, 1.2 Hz, 2H), 7.84 (dd, *J* = 8.3, 1.1 Hz, 2H), 7.79 (dd, *J* = 8.3, 1.2 Hz, 2H), 7.72 (dd, *J* = 8.2, 1.1 Hz, 2H), 7.70–7.65 (m, 1H), 7.61 (td, *J* = 7.4, 1.6 Hz, 2H), 7.55 (q, *J* = 8.0 Hz, 3H), 7.46 (dd, *J* = 14.0, 7.6 Hz, 4H), 7.41 (t, *J* = 7.8 Hz, 2H), 6.06 (t, *J* = 9.5 Hz, 1H), 5.81 (t, *J* = 9.4 Hz, 1H), 5.59 (t, *J* = 9.7 Hz, 1H), 5.36 (t, *J* = 9.4 Hz, 1H), 4.64–4.57 (m, 1H), 4.52 (dd, *J* = 12.4, 2.4 Hz, 1H), 4.45 (dd, *J* = 12.5, 3.8 Hz, 1H), 2.25 (t, *J* = 7.4 Hz, 3H), 2.17–2.02 (m, 4H) ppm. ^13^C-NMR (DMSO-*d_6_*, 151 MHz) *δ* 174.56, 172.88, 165.79, 165.53, 165.23, 134.50, 134.11, 133.99, 129.81, 129.65, 129.48, 129.15, 129.02, 77.66, 74.36, 72.77, 72.14, 69.43, 62.95, 60.23, 34.83, 33.22, 20.75 ppm.

#### 3.1.4. Synthesis of p-azidobenzenesulfonamide (**8**)

Intermediate **8** was synthesised as reported previously [27] and was used without further purification.

#### 3.1.5. General Procedure of Synthesis of N-(4-Bromophenyl)acetamide Derivatives (**10a–g**)

To a solution of appropriate 4-bromoaniline (5.8 mmol) in EtOAc (15 mL), Ac_2_O (0.8 mL, 8.7 mmol) was added. The solution was stirred at room temperature for 3 h. After the reaction completed (monitored by TLC), the mixture was washed with H_2_O (20 mL) saturated sodium bicarbonate solution (15 mL) and brine (20 mL) and dried over Na_2_SO_4_. After filtration and evaporation of EtOAc, the product was used without further purification. Yield 80–95%.

*N*-(4-bromophenyl)acetamide (**10a**): white solid (1.2 g, 95% yield), m.p. 165–166 °C. ^1^H-NMR (DMSO-*d_6_*, 600 MHz) *δ* 10.06 (s, 1H), 7.60–7.52 (m, 2H), 7.49–7.42 (m, 2H), 2.04 (s, 3H) ppm. ^13^C-NMR (DMSO-*d_6_*, 151 MHz) *δ* 168.93, 139.15, 131.94, 121.27, 114.94, 24.49 ppm.

*N*-(3-bromophenyl)acetamide (**10b**): white solid (1.2 g, 94% yield), m.p. 85–86 °C. ^1^H-NMR (DMSO-*d_6_*, 600 MHz) *δ* 10.10 (s, 1H), 7.95 (t, *J* = 1.9 Hz, 1H), 7.55–7.37 (m, 1H), 7.26 (t, *J* = 8.0 Hz, 1H), 7.22 (d, *J* = 8.0 Hz, 1H), 2.05 (s, 3H) ppm. ^13^C-NMR (DMSO-*d_6_*, 151 MHz) *δ* 169.14, 141.35, 131.17, 126.04, 121.98, 121.69, 118.12, 24.51 ppm.

*N*-(4-bromo-2-fluorophenyl)acetamide (**10c**): white solid (1.2 g, 86% yield), m.p. 157–158 °C. ^1^H-NMR (DMSO-*d_6_*, 600 MHz) *δ* 9.82 (s, 1H), 7.89 (t, *J* = 8.6 Hz, 1H), 7.57 (d, *J* = 10.5 Hz, 1H), 7.36 (d, *J* = 8.7 Hz, 1H), 2.09 (s, 3H) ppm. ^13^C-NMR (DMSO-*d_6_*, 151 MHz) *δ* 169.27, 154.52, 152.87, 127.83, 126.44, 125.65, 119.29, 119.14, 115.78, 23.97 ppm.

*N*-(4-bromo-2-chlorophenyl)acetamide (**10d**): light yellow solid (1.2 g, 85% yield), m.p. 151–153 °C. ^1^H-NMR (DMSO-*d_6_*, 600 MHz) *δ* 9.58 (s, 1H), 7.76 (s, 1H), 7.70 (d, *J* = 8.7 Hz, 1H), 7.57–7.42 (m, 1H), 2.10 (s, 3H) ppm. ^13^C-NMR (DMSO-*d_6_*, 151 MHz) *δ* 169.19, 135.10, 131.99, 130.82, 127.84, 117.36, 23.83 ppm.

*N*-(4-bromo-2-methylphenyl)acetamide (**10e**): white solid (1.1 g, 86% yield), m.p. 153-154 °C. ^1^H-NMR (DMSO-*d_6_*, 600 MHz) *δ* 9.31 (s, 1H), 7.41 (dd, *J* = 7.4, 5.4 Hz, 2H), 7.32 (dd, *J* = 8.5, 2.1 Hz, 1H), 2.19 (s, 3H), 2.06 (s, 3H) ppm. ^13^C-NMR (DMSO-*d_6_*, 151 MHz) *δ* 168.77, 136.44, 134.49, 133.09, 129.11, 127.05, 117.34, 23.79, 18.05 ppm.

*N*-(4-bromo-2-methoxyphenyl)acetamide (**10f**): white solid (1.1 g, 86% yield), m.p. 156–158 °C. ^1^H-NMR (DMSO-*d_6_*, 600 MHz) *δ* 9.21 (s, 1H), 7.91 (d, *J* = 8.6 Hz, 1H), 7.21 (d, *J* = 2.1 Hz, 1H), 7.08 (dd, *J* = 8.6, 2.1 Hz, 1H), 3.85 (s, 3H), 2.08 (s, 3H) ppm. ^13^C-NMR (DMSO-*d_6_*, 151 MHz) *δ* 169.09, 150.81, 127.42, 123.59, 123.34, 116.06, 114.71, 56.58, 24.30 ppm.

*N*-(4-bromo-2,6-difluorophenyl)acetamide (**10g**): light yellow solid (1.2 g, 80% yield), m.p. 189–191 °C. ^1^H-NMR (DMSO-*d_6_*, 600 MHz) *δ* 9.74 (s, 1H), 7.54 (d, *J* = 7.2 Hz, 2H), 2.06 (s, 3H). ppm. ^13^C-NMR (DMSO-*d_6_*, 151 MHz) *δ* 168.76, 158.96, 158.92, 157.29, 157.24, 118.96, 118.88, 118.80, 116.25, 116.22, 116.07, 115.12, 115.01, 114.90, 22.86 ppm.

#### 3.1.6. General Procedure of Synthesis of N-(4-((trimethylsilyl)ethynyl)phenyl)acetamide Derivatives (**11a–g**)

To a solution of different intermediates **10a–g** (5 mmol), Pd(PPh_3_)_2_Cl_2_ (0.18 g, 0.25 mmol) and CuI (0.095 g, 0.5 mmol) in dry DMF (30 mL), TEA (1.4 mL, 10 mmol) was added. After degassing, trimethylsilylacetylene (1.1 mL, 7.5 mmol) was added to the reaction mixture under Ar atmosphere. The solution was stirred at 80 °C for 30 h. Once the reaction was completed (monitored by TLC), the mixture was diluted with EtOAc (100 mL) and filtered through the celite. The filtrate was washed with H_2_O (50 mLX3) and brine (50 mL) before dried over Na_2_SO_4_. After filtration and evaporation of EtOAc, the intermediates **11a–g** were isolated through silica column chromatograph (PE:EA=8:1).

*N*-(4-((trimethylsilyl)ethynyl)phenyl)acetamide (**11a**): white solid (0.68 g, 61% yield), m.p. 331–333 °C. ^1^H-NMR (DMSO-*d_6_*, 600 MHz) *δ* 10.10 (s, 1H), 7.58 (d, *J* = 8.7 Hz, 2H), 7.40–7.34 (m, 2H), 2.05 (s, 3H), 0.21 (s, 9H) ppm. ^13^C-NMR (DMSO-*d_6_*, 151 MHz) *δ* 169.50, 140.85, 133.23, 119.57, 117.19, 106.40, 93.82, 25.03, 0.92 ppm.

*N*-(3-((trimethylsilyl)ethynyl)phenyl)acetamide (**11b**): white solid (0.70 g, 62% yield), m.p. 342–346 °C. ^1^H-NMR (DMSO-*d_6_*, 600 MHz) *δ* 10.01 (s, 1H), 7.79 (s, 1H), 7.48 (ddd, *J* = 8.2, 2.0, 0.9 Hz, 1H), 7.28 (t, *J* = 7.9 Hz, 1H), 7.12–7.07 (m, 1H), 2.04 (s, 3H), 0.23 (s, 9H) ppm. ^13^C-NMR (DMSO-*d_6_*, 151 MHz) *δ* 169.49, 140.40, 130.09, 126.98, 123.32, 122.58, 120.47, 106.10, 94.75, 24.97, 0.81 ppm.

*N*-(2-fluoro-4-((trimethylsilyl)ethynyl)phenyl)acetamide (**11c**): light yellow solid (0.81 g, 64% yield), m.p. 352–355 °C. ^1^H-NMR (DMSO-*d_6_*, 600 MHz) *δ* 9.86 (s, 1H), 8.02 (t, *J* = 8.2 Hz, 1H), 7.35 (dd, *J* = 11.5, 1.7 Hz, 1H), 7.24 (dd, *J* = 8.4, 1.2 Hz, 1H), 2.10 (s, 3H), 0.22 (s, 9H) ppm. ^13^C-NMR (DMSO-*d_6_*, 151 MHz) *δ* 169.90, 153.84, 152.21, 129.11, 128.58, 128.50, 123.95, 119.25, 119.11, 118.75, 118.69, 104.82, 95.30, 24.61, 0.78 ppm.

*N*-(2-chloro-4-((trimethylsilyl)ethynyl)phenyl)acetamide (**11d**): light yellow solid (0.63 g, 47% yield), m.p. 337–338 °C. ^1^H-NMR (DMSO-*d_6_*, 600 MHz) *δ* 9.56 (s, 1H), 7.84 (d, *J* = 8.4 Hz, 1H), 7.57 (s, 1H), 7.39 (d, *J* = 8.5 Hz, 1H), 2.12 (s, 3H), 0.23 (s, 9H) ppm. ^13^C-NMR (DMSO-*d_6_*, 151 MHz) *δ* 169.78, 136.80, 133.07, 131.62, 125.90, 120.04, 104.37, 95.93, 52.14, 24.49, 0.75 ppm.

*N*-(2-methyl-4-((trimethylsilyl)ethynyl)phenyl)acetamide (**11e**): white solid (0.69 g, 57% yield), m.p. 360–362 °C. ^1^H-NMR (DMSO-*d_6_*, 600 MHz) *δ* 9.28 (s, 1H), 7.55 (d, *J* = 8.2 Hz, 1H), 7.31 (s, 1H), 7.23 (dd, *J* = 8.3, 1.8 Hz, 1H), 2.19 (s, 3H), 2.07 (s, 3H), 0.21 (s, 9H) ppm. ^13^C-NMR (DMSO-*d_6_*, 151 MHz) *δ* 169.28, 138.33, 134.42, 131.76, 130.20, 124.93, 118.88, 106.27, 94.09, 24.43, 18.48, 0.90 ppm.

*N*-(2-methoxy-4-((trimethylsilyl)ethynyl)phenyl)acetamide (**11f**): white solid (0.69 g, 50% yield), m.p. 340–342 °C. ^1^H-NMR (DMSO-*d_6_*, 600 MHz) *δ* 9.22 (s), 8.03 (d, *J* = 8.2 Hz), 7.05 (d, *J* = 1.7 Hz), 7.01 (dd, *J* = 8.3, 1.7 Hz), 3.85 (s), 2.10 (s), 0.22 (s) ppm. ^13^C-NMR (DMSO-*d_6_*, 151 MHz) *δ* 169.66, 149.49, 129.63, 125.20, 121.74, 118.01, 114.63, 106.45, 93.83, 56.79, 24.90, 0.88 ppm.

*N*-(2,6-difluoro-4-((trimethylsilyl)ethynyl)phenyl)acetamide (**11g**): light yellow solid (0.62 g, 46% yield), m.p. 368–370 °C. ^1^H-NMR (DMSO-*d_6_*, 600 MHz) *δ* 9.80 (s, 1H), 7.29 (d, *J* = 8.0 Hz, 2H), 2.06 (s, 3H), 0.23 (s, 9H) ppm. ^13^C-NMR (DMSO-*d_6_*, 151 MHz) *δ* 169.13, 158.90, 158.86, 157.25, 157.20, 122.01, 121.93, 121.86, 117.20, 117.08, 116.97, 116.19, 116.16, 116.05, 116.02, 103.34, 97.54, 23.38, 0.62 ppm.

#### 3.1.7. Synthesis of 2-Nitro-4-((trimethylsilyl)ethynyl)aniline (**11h**)

4-Iodo-2-nitroaniline (1.3 g, 5.0 mmol) was dissolved in dry THF (25 mL). Pd (PPh_3_)_2_Cl_2_ (0.35 g, 0.5 mmol), CuI (0.19 g, 1 mmol), and DIEA (2.6 mL, 15.0 mmol) was added to the solution. After degassing, trimethylsilylacetylene (1.1 mL, 7.5 mmol) was added into the reaction mixture under Ar atmosphere. The mixture was stirred at room temperature for 3 h (monitored by TLC); then, H_2_O (30 mL) was added and extracted with EtOAc (30 mL). The combined organic layer was washed with brine (30 mL) and dried over Na_2_SO_4_. After the filtration and evaporation of EtOAc, the compound **11h** was isolated through a silica column chromatograph (PE:EA=4:1). Kermesinus solid (0.53 g, 45%), m.p. 316–319 °C. ^1^H-NMR (DMSO-*d_6_*, 600 MHz) *δ* 7.98 (d, *J* = 2.0 Hz, 1H), 7.74 (s, 2H), 7.41 (dd, *J* = 8.8, 2.0 Hz, 1H), 6.98 (d, *J* = 8.8 Hz, 1H), 0.21 (s, 9H) ppm. ^13^C-NMR (DMSO-*d_6_*, 151 MHz) *δ* 147.15, 138.67, 130.54, 129.98, 120.70, 109.48, 105.04, 93.35, 0.88 ppm.

#### 3.1.8. General Procedure of Synthesis of N-(4-Ethynylphenyl)acetamide Derivatives (**12a–h**)

Intermediates **12a–h** were synthesized from compounds **11a–h** by deprotection of trimethylsilyl using TBAF [28] and extraction after the reaction and used without further purification.

#### 3.1.9. General Procedure of Synthesis of N-(4-(1-(4-sulfamoylphenyl)-1H-1,2,3-triazol-4-yl)phenyl)acetamide derivatives (**13a–g**) and 4-(4-(4-amino-3-nitrophenyl)-1H-1,2,3-triazol-1-yl)benzenesulfonamide (**14h**)

The appropriate 4-ethynylbenzenamine derivatives **12a–h** (3 mmol) were added to a suspension of compound **8** (0.65 g, 3.3 mmol) in *t*-BuOH-H_2_O 1:1 (20 mL) at r.t., which was followed by CuSO_4_ (0.14 g, 0.9 mmol) and sodium ascorbate (0.36g, 1.8 mmol). The suspension was stirred at 60 °C until the starting materials were consumed (monitored by TLC); then, they were quenched with H_2_O (20 mL), and the formed precipitate was filtered off and washed with H_2_O affording the compounds **13a–g** and **14h**, which were used without further purification.

4-(4-(4-Amino-3-nitrophenyl)-1*H*-1,2,3-triazol-1-yl)benzenesulfonamide (**14h**): Kermesinus solid (0.37 g, 34%), m.p. 222–225 °C. ^1^H-NMR (DMSO-*d_6_*, 600 MHz) *δ* 9.41 (s, 1H), 8.55 (d, *J* = 1.9 Hz, 1H), 8.18 (d, *J* = 8.7 Hz, 2H), 8.07 (d, *J* = 8.7 Hz, 2H), 7.98 (dd, *J* = 8.8, 1.9 Hz, 1H), 7.67 (s, 2H), 7.55 (s, 2H), 7.17 (d, *J* = 8.8 Hz, 1H) ppm. ^13^C-NMR (DMSO-*d_6_*, 151 MHz) *δ* 146.88, 146.58, 144.25, 139.07, 133.31, 130.65, 128.05, 122.23, 120.59, 120.53, 119.40, 117.99 ppm.

#### 3.1.10. General Procedure of Synthesis of 4-(4-(4-aminophenyl)-1H-1,2,3-triazol-1-yl)benzenesulfonamide Derivatives (**14a–g**)

The appropriate intermediates **13a–g** (1.6 mmol) were suspended in a 5 N sodium hydroxide aqueous solution (20 mL). The suspension was refluxed for 1.5 h. Once the reaction was completed (monitored by TLC), it was cooled to room temperature and filtered. The solid was dissolved in MeOH/EtOH and purified chromatographically (DCM:MeOH = 60:1) to provide compounds **14a–g**.

4-(4-(4-Aminophenyl)-1*H*-1,2,3-triazol-1-yl)benzenesulfonamide (**14a**): Khaki solid (0.41 g, 81% yield), m.p. 256–258 °C. ^1^H-NMR (DMSO-*d_6_*, 600 MHz) *δ* 9.11 (s, 1H), 8.09 (d, *J* = 56.1 Hz, 4H), 7.61 (s, 2H), 7.45 (s, 2H), 6.67 (s, 2H), 5.34 (s, 2H) ppm. ^13^C-NMR (DMSO-*d_6_*, 151 MHz) *δ* 149.63, 149.13, 139.16, 127.97, 126.95, 120.33, 117.83, 117.72, 114.44 ppm.

4-(4-(3-Aminophenyl)-1*H*-1,2,3-triazol-1-yl)benzenesulfonamide (**14b**): Khaki solid (0.40 g, 79% yield), m.p. 270–273 °C. ^1^H-NMR (DMSO-*d_6_*, 600 MHz) *δ* 9.20 (s, 1H), 8.05 (dd, *J* = 48.3, 6.0 Hz, 4H), 7.22 (s, 1H), 7.11 (dd, *J* = 39.5, 6.5 Hz, 2H), 6.61 (d, *J* = 6.4 Hz, 1H) ppm. ^13^C-NMR (DMSO-*d_6_*, 151 MHz) *δ* 149.63, 148.72, 138.24, 130.98, 129.93, 127.65, 120.27, 119.72, 114.50, 113.72, 111.08 ppm.

4-(4-(4-Amino-3-fluorophenyl)-1*H*-1,2,3-triazol-1-yl)benzenesulfonamide (**14c**): Brown solid (0.41 g, 77% yield), m.p. 245–247 °C. ^1^H-NMR (DMSO-*d_6_*, 600 MHz) *δ* 9.16 (s, 1H), 8.02 (s, 3H), 7.69–7.38 (m, 2H), 6.86 (t, *J* = 8.7 Hz, 1H), 5.37 (d, *J* = 23.4 Hz, 2H) ppm. ^13^C-NMR (DMSO-*d_6_*, 151 MHz) *δ* 151.80, 150.23, 147.95, 137.26, 127.67, 122.54, 122.36, 120.13, 118.55, 116.79, 112.63, 112.50 ppm.

4-(4-(4-Amino-3-chlorophenyl)-1*H*-1,2,3-triazol-1-yl)benzenesulfonamide (**14d**): Brown solid (0.32 g, 57% yield), m.p. 201–203 °C. ^1^H-NMR (DMSO-*d_6_*, 600 MHz) *δ* 99.24 (s, 1H), 8.14 (d, *J* = 8.8 Hz, 2H), 8.05 (d, *J* = 8.8 Hz, 2H), 7.77 (d, *J* = 1.9 Hz, 1H), 7.62 (dd, *J* = 8.3, 1.9 Hz, 1H), 7.53 (s, 2H), 6.90 (d, *J* = 8.4 Hz, 1H), 5.63 (s, 2H) ppm. ^13^C-NMR (DMSO-*d_6_*, 151 MHz) *δ* 147.76, 145.44, 144.13, 139.14, 128.05, 126.53, 125.52, 120.42, 119.19, 118.58, 117.74, 116.06 ppm.

4-(4-(4-Amino-3-methylphenyl)-1*H*-1,2,3-triazol-1-yl)benzenesulfonamide (**14e**): Brown solid (0.45 g, 85% yield), m.p. 264–266 °C. ^1^H-NMR (DMSO-*d_6_*, 600 MHz) *δ* 9.18 (s, 1H), 8.24–8.19 (m, 2H), 8.12–8.08 (m, 2H), 7.60 (s, 1H), 7.59 (s, 2H), 7.55 (dd, *J* = 8.1, 1.8 Hz, 1H), 6.76 (d, *J* = 8.2 Hz, 1H), 5.18 (s, 2H), 2.20 (s, 3H) ppm. ^13^C-NMR (DMSO-*d_6_*, 151 MHz) *δ* 149.22, 147.63, 143.94, 139.24, 128.00, 127.82, 124.54, 121.67, 120.31, 118.08, 117.72, 114.49, 18.01 ppm.

4-(4-(4-Amino-3-methoxyphenyl)-1*H*-1,2,3-triazol-1-yl)benzenesulfonamide (**14f**): Brown solid (0.49 g, 89% yield), m.p. 230–233 °C. ^1^H-NMR (DMSO-*d_6_*, 600 MHz) *δ* 9.19 (s, 1H), 8.19–8.12 (m, 2H), 8.07–8.02 (m, 2H), 7.53 (s, 2H), 7.37 (d, *J* = 1.7 Hz, 1H), 7.30 (dd, *J* = 8.0, 1.8 Hz, 1H), 6.72 (d, *J* = 8.0 Hz, 1H), 4.99 (s, 2H), 3.87 (s, 3H) ppm. ^13^C-NMR (DMSO-*d_6_*, 151 MHz) *δ* 149.23, 146.88, 144.00, 139.22, 138.76, 128.02, 120.32, 118.98, 118.34, 118.07, 114.12, 108.30, 55.83 ppm.

4-(4-(4-Amino-3,5-difluorophenyl)-1*H*-1,2,3-triazol-1-yl)benzenesulfonamide (**14g**): Brown solid (0.36 g, 64% yield), m.p. 280–283 °C. ^1^H-NMR (DMSO-*d_6_*, 600 MHz) *δ* 9.29 (s, 1H), 8.18–8.09 (m, 2H), 8.09–8.03 (m, 2H), 7.54 (s, 2H), 7.48 (dd, *J* = 7.4, 2.2 Hz, 2H), 5.51 (s, 2H) ppm. ^13^C-NMR (DMSO-*d_6_*, 151 MHz) *δ* 152.50, 152.43, 150.92, 150.85, 147.12, 144.28, 139.04, 128.09, 126.47, 126.36, 126.25, 120.52, 119.40, 116.93, 116.87, 116.81, 108.82, 108.72, 108.65, 108.61 ppm.

#### 3.1.11. General Procedure of Synthesis of [(2,3,4,6-Tetra-O-Benzoyl-β-D-Glucopyranosyl)amino] Amide Derivatives (**15a–o**)

Appropriate [(2,3,4,6-tetra-*O*-benzoyl-β-D-glucopyranosyl)amino] acid derivatives **5/6** (1.0 mmol) and EDCI (0.29 g, 1.5 mmol) were dissolved in anhydrous Py (25 mL) and stirred for 1 h. Then, the appropriate **14** (1.0 mmol) was added. The mixture was stirred at room temperature for 8 h. After the reaction completed (monitored by TLC), the mixture was diluted with EtOAc (40 mL) and washed with H_2_O (3 × 20 mL) and brine (50 mL) before being dried over Na_2_SO_4_. After the filtration and evaporation of EtOAc, the crude intermediates **15a–o** were used without further purification.

#### 3.1.12. General Procedure of Synthesis of β-D-Glucopyranosyl)amino] Amide Derivatives (**16a–o**)

The crude intermediates **15a–o** obtained in the previous step were dissolved in MeOH (15 mL), and a newly prepared solution of NaOMe in MeOH (1.0 mol/L, 1 mL) was added. The solution was stirred at room temperature for 2.5 h and then neutralised with 6 N HCl solution to pH 7. After evaporation of MeOH, the residue was purified chromatographically (DCM:MeOH = 8:1) to provide compounds **16a–o**.

*N*^1^-(4-(1-(4-sulfamoylphenyl)-1*H*-1,2,3-triazol-4-yl)phenyl)-*N*^4^-((2*R*,3*R*,4*S*,5*S*,6*R*)-3,4,5-trihydroxy-6-(hydroxymethyl)tetrahydro-2*H*-pyran-2-yl)succinamide (**16a**): ^1^H-NMR (600 MHz, DMSO-*d_6_*) *δ* 10.16 (s, 1H), 9.33 (s, 1H), 8.51 (d, *J* = 9.0 Hz, 1H), 8.17 (d, *J* = 8.7 Hz, 2H), 8.06 (d, *J* = 8.7 Hz, 2H), 7.88 (d, *J* = 8.6 Hz, 2H), 7.73 (d, *J* = 8.6 Hz, 2H), 7.54 (s, 2H), 5.10–4.90 (m, 2H), 4.71 (t, *J* = 9.0 Hz, 1H), 4.53 (s, 1H), 3.64 (d, *J* = 10.7 Hz, 1H), 3.45–3.40 (m, 1H), 3.18 (dd, *J* = 11.5, 5.5 Hz, 1H), 3.12–3.01 (m, 3H), 2.63–2.56 (m, 2H), 2.47 (dd, *J* = 15.9, 7.7 Hz, 1H); ^13^C-NMR (151 MHz, DMSO-*d_6_*) *δ* 171.08, 170.03, 146.90, 143.14, 138.92, 138.03, 126.94, 125.25, 123.89, 119.50, 118.63, 118.48, 78.97, 77.93, 76.88, 71.86, 69.32, 60.25, 30.68, 29.62; HRMS (ESI): Calcd. for [M-H]^−^ C_24_H_27_N_6_O_9_S^−^: 575.1638, Found 575.1631.

*N*^1^-(3-(1-(4-sulfamoylphenyl)-1*H*-1,2,3-triazol-4-yl)phenyl)-*N*^4^-((2*R*,3*R*,4*S*,5*S*,6*R*)-3,4,5-trihydroxy-6-(hydroxymethyl)tetrahydro-2*H*-pyran-2-yl)succinamide (**16b**): ^1^H-NMR (400 MHz, DMSO-*d_6_*) *δ* 10.14 (s, 1H), 9.37 (s, 1H), 8.49 (d, *J* = 8.9 Hz, 1H), 8.32 (s, 1H), 8.21 (d, *J* = 8.7 Hz, 2H), 8.05 (d, *J* = 8.7 Hz, 2H), 7.56 (dd, *J* = 11.0, 9.7 Hz, 4H), 7.42 (t, *J* = 7.9 Hz, 1H), 4.95 (d, *J* = 22.5 Hz, 3H), 4.72 (t, *J* = 9.0 Hz, 1H), 4.50 (s, 1H), 3.64 (d, *J* = 11.5 Hz, 1H), 3.42 (d, *J* = 10.3 Hz, 1H), 3.24–3.13 (m, 1H), 3.07 (q, *J* = 9.4 Hz, 3H), 2.67–2.55 (m, 2H); ^13^C-NMR (101 MHz, DMSO-*d_6_*) *δ* 172.45, 171.19, 148.08, 144.10, 140.18, 139.10, 130.75, 129.97, 128.06, 120.79, 120.26, 119.55, 116.35, 79.89, 78.81, 77.64, 72.72, 70.25, 61.23, 31.57, 30.61; HRMS (ESI): Calcd. for [M-H]^−^ C_24_H_27_N_6_O_9_S^−^: 575.1638, Found 575.1629.

*N*^1^-(2-fluoro-4-(1-(4-sulfamoylphenyl)-1*H*-1,2,3-triazol-4-yl)phenyl)-*N*^4^-((2*R*,3*R*,4*S*,5*S*,6*R*)-3,4,5-trihydroxy-6-(hydroxymethyl)tetrahydro-2*H*-pyran-2-yl)succinamide (**16c**): ^1^H-NMR (600 MHz, DMSO-*d_6_*) *δ* 9.93 (s, 1H), 9.44 (s, 1H), 8.54 (d, *J* = 8.9 Hz, 1H), 8.16 (d, *J* = 8.6 Hz, 2H), 8.07 (d, *J* = 8.6 Hz, 3H), 7.79 (d, *J* = 11.8 Hz, 1H), 7.74 (d, *J* = 8.4 Hz, 1H), 7.56 (s, 2H), 5.07 (s, 2H), 4.98 (s, 1H), 4.71 (t, *J* = 9.0 Hz, 1H), 4.54 (s, 1H), 3.63 (d, *J* = 11.2 Hz, 1H), 3.42 (d, *J* = 10.8 Hz, 1H), 3.18 (t, *J* = 8.5 Hz, 1H), 3.13–3.01 (m, 3H), 2.72–2.58 (m, 2H), 2.49–2.40 (m, 2H); ^13^C-NMR (151 MHz, DMSO-*d_6_*) *δ* 171.59, 170.98, 146.31, 143.82, 138.42, 127.49, 121.10, 120.09, 119.96, 79.52, 78.44, 77.36, 72.32, 69.81, 60.75, 30.78, 30.14; HRMS (ESI): Calcd. for [M-H]^−^ C_24_H_26_FN_6_O_9_S^−^: 593.1544, Found 593.1541.

*N*^1^-(2-chloro-4-(1-(4-sulfamoylphenyl)-1*H*-1,2,3-triazol-4-yl)phenyl)-*N*^4^-((2*R*,3*R*,4*S*,5*S*,6*R*)-3,4,5-trihydroxy-6-(hydroxymethyl)tetrahydro-2*H*-pyran-2-yl)succinamide (**16d**): ^1^H-NMR (600 MHz, DMSO-*d_6_*) *δ* 12.29 (s, 1H), 9.26 (s, 1H), 8.36 (d, *J* = 9.1 Hz, 1H), 8.15 (dd, *J* = 24.2, 8.8 Hz, 4H), 7.77 (d, *J* = 1.8 Hz, 1H), 7.62 (dd, *J* = 8.3, 1.8 Hz, 1H), 6.90 (d, *J* = 8.4 Hz, 1H), 5.64 (s, 2H), 4.95 (d, *J* = 4.3 Hz, 1H), 4.84 (dd, *J* = 19.3, 5.1 Hz, 2H), 4.65 (t, *J* = 9.1 Hz, 1H), 4.49 (t, *J* = 5.6 Hz, 1H), 3.60 (dd, *J* = 11.7, 4.0 Hz, 1H), 3.42–3.36 (m, 1H), 3.14 (td, *J* = 8.7, 4.0 Hz, 1H), 3.08–2.96 (m, 3H), 2.47–2.39 (m, 2H), 2.37–2.23 (m, 2H); ^13^C-NMR (151 MHz, DMSO-*d_6_*) *δ* 171.75, 171.63, 147.82, 145.36, 140.26, 139.39, 130.03, 126.55, 125.57, 120.45, 119.12, 118.60, 117.73, 116.03, 79.93, 78.86, 77.68, 72.79, 70.18, 61.12, 30.89, 29.50; HRMS (ESI): Calcd. for [M-H]^−^ C_24_H_26_ClN_6_O_9_S^−^: 609.1249, Found 609.1242.

*N*^1^-(2-methyl-4-(1-(4-sulfamoylphenyl)-1*H*-1,2,3-triazol-4-yl)phenyl)-*N*^4^-((2*R*,3*R*,4*S*,5*S*,6*R*)-3,4,5-trihydroxy-6-(hydroxymethyl)tetrahydro-2*H*-pyran-2-yl)succinamide (**16e**): ^1^H-NMR (600 MHz, DMSO-*d_6_*) *δ* 9.39 (d, *J* = 18.3 Hz, 2H), 8.51 (d, *J* = 9.0 Hz, 1H), 8.18 (d, *J* = 8.7 Hz, 2H), 8.06 (d, *J* = 8.7 Hz, 2H), 7.80 (s, 1H), 7.73 (d, *J* = 9.3 Hz, 1H), 7.61 (d, *J* = 8.2 Hz, 1H), 7.55 (s, 3H), 4.74 (s, 1H), 3.64 (d, *J* = 10.6 Hz, 1H), 3.42 (dd, *J* = 11.8, 5.2 Hz, 2H), 3.19 (t, *J* = 8.7 Hz, 1H), 3.14–2.99 (m, 3H), 2.67–2.57 (m, 2H), 2.30 (s, 4H); ^13^C-NMR (151 MHz, DMSO-*d_6_*) *δ* 171.19, 169.99, 146.86, 143.16, 138.03, 136.12, 131.03, 126.96, 126.60, 125.65, 124.37, 122.43, 119.50, 118.80, 79.00, 77.95, 76.88, 71.88, 69.35, 60.29, 30.33, 30.01, 17.42; HRMS (ESI): Calcd. for [M-H]^−^ C_25_H_29_N_6_O_9_S^−^: 589.1795, Found 589.1787.

*N*^1^-(2-methoxy-4-(1-(4-sulfamoylphenyl)-1*H*-1,2,3-triazol-4-yl)phenyl)-*N*^4^-((2*R*,3*R*,4*S*,5*S*,6*R*)-3,4,5-trihydroxy-6-(hydroxymethyl)tetrahydro-2*H*-pyran-2-yl)succinamide (**16f**): ^1^H-NMR (600 MHz, DMSO-*d_6_*) *δ* 9.41 (s, 1H), 9.29 (s, 1H), 8.59 (d, *J* = 8.8 Hz, 1H), 8.17 (d, *J* = 8.6 Hz, 2H), 8.14 (d, *J* = 8.2 Hz, 1H), 8.07 (d, *J* = 8.6 Hz, 2H), 7.60 (s, 1H), 7.56 (s, 2H), 7.50 (d, *J* = 9.5 Hz, 1H), 5.18 (s, 2H), 5.04 (s, 1H), 4.70 (t, *J* = 9.0 Hz, 1H), 4.56 (s, 1H), 3.95 (s, 3H), 3.63 (d, *J* = 11.0 Hz, 1H), 3.42 (d, *J* = 11.5 Hz, 1H), 3.18 (t, *J* = 8.4 Hz, 1H), 3.10–3.01 (m, 3H), 2.66 (dd, *J* = 25.0, 17.2 Hz, 2H), 2.46 (dd, *J* = 16.1, 8.2 Hz, 2H); ^13^C-NMR (151 MHz, DMSO-*d_6_*) *δ* 172.29, 171.31, 150.01, 148.08, 144.29, 139.09, 128.26, 128.06, 126.06, 122.13, 120.55, 119.94, 117.87, 108.44, 80.14, 79.03, 77.93, 72.87, 70.40, 61.34, 56.31, 31.76, 30.94; HRMS (ESI): Calcd. for [M-H]^−^ C_25_H_29_N_6_O_10_S^−^: 605.1744, Found 605.1740.

*N*^1^-(2,6-difluoro-4-(1-(4-sulfamoylphenyl)-1*H*-1,2,3-triazol-4-yl)phenyl)-*N*^4^-((2*R*,3*R*,4*S*,5*S*,6*R*)-3,4,5-trihydroxy-6-(hydroxymethyl)tetrahydro-2*H*-pyran-2-yl)succinamide (**16g**): ^1^H-NMR (600 MHz, DMSO-*d_6_*) *δ* 9.25 (d, *J* = 43.4 Hz), 8.39–8.17 (m), 8.02 (ddd, *J* = 36.2, 32.2, 8.6 Hz), 7.54 (s), 7.48 (d, *J* = 7.4 Hz), 5.49 (d, *J* = 17.4 Hz), 4.96 (s), 4.85 (s), 4.67 (t, *J* = 9.0 Hz), 4.52 (s), 3.67–3.56 (m), 3.43–3.37 (m), 3.16 (dd, *J* = 17.5, 8.7 Hz), 3.05 (dd, *J* = 16.8, 7.4 Hz), 2.45–2.17 (m); ^13^C-NMR (151 MHz, DMSO-*d_6_*) *δ* 172.96, 171.87, 152.50, 152.43, 150.92, 150.86, 147.11, 146.91, 144.27, 139.03, 137.88, 129.15, 128.09, 126.47, 126.36, 126.28, 126.21, 126.10, 120.52, 119.55, 119.40, 119.33, 117.11, 116.87, 116.80, 108.76, 108.72, 108.68, 108.65, 108.61, 108.57, 80.01, 78.99, 77.97, 72.99, 70.40, 61.31, 30.56, 29.29; HRMS (ESI): Calcd. for [M-H]^−^ C_24_H_25_F_2_N_6_O_9_S^−^: 611.1450, Found 611.1443.

*N*^1^-(4-(1-(4-sulfamoylphenyl)-1*H*-1,2,3-triazol-4-yl)phenyl)-*N*^5^-((2*R*,3*R*,4*S*,5*S*,6*R*)-3,4,5-trihydroxy-6-(hydroxymethyl)tetrahydro-2*H*-pyran-2-yl)glutaramide (**16h**): ^1^H-NMR (600 MHz, DMSO-*d_6_*) *δ* 10.13 (s), 9.36 (s), 8.18 (d, *J* = 8.2 Hz), 8.06 (d, *J* = 8.3 Hz), 7.88 (d, *J* = 7.9 Hz), 7.75 (d, *J* = 7.6 Hz), 7.55 (s), 5.01 (s), 4.90 (dd, *J* = 12.8, 4.3 Hz), 4.72 (t, *J* = 8.5 Hz), 4.51 (s), 3.64 (dd, *J* = 10.6, 4.3 Hz), 3.42 (d, *J* = 6.1 Hz), 3.18 (s), 3.07 (dd, *J* = 9.7, 4.7 Hz), 3.03–2.93 (m), 2.64–2.53 (m), 2.41–2.33 (m), 2.20 (ddd, *J* = 27.9, 14.0, 6.9 Hz), 1.89–1.78 (m);^13^C-NMR (151 MHz, DMSO-*d_6_*) *δ* 172.62, 171.57, 147.99, 144.23, 139.98, 139.11, 128.04, 126.32, 125.05, 120.59, 119.84, 119.58, 79.95, 79.02, 78.00, 77.91, 72.93, 70.46, 61.41, 36.23, 35.05, 21.23; HRMS (ESI): Calcd. for [M-H]^−^ C_25_H_29_N_6_O_9_S^−^: 589.1795, Found 589.1788.

*N*^1^-(3-(1-(4-sulfamoylphenyl)-1*H*-1,2,3-triazol-4-yl)phenyl)-*N*^5^-((2*R*,3*R*,4*S*,5*S*,6*R*)-3,4,5-trihydroxy-6-(hydroxymethyl)tetrahydro-2*H*-pyran-2-yl)glutaramide (**16i**): ^1^H-NMR (400 MHz, DMSO-*d_6_*) *δ* 10.06 (s, 1H), 9.38 (s, 1H), 8.41–8.27 (m, 2H), 8.21 (d, *J* = 8.5 Hz, 2H), 8.06 (d, *J* = 8.5 Hz, 2H), 7.59 (t, *J* = 7.4 Hz, 2H), 7.53 (s, 2H), 7.43 (t, *J* = 7.8 Hz, 1H), 4.98 (s, 1H), 4.88 (s, 2H), 4.73 (t, *J* = 9.0 Hz, 1H), 4.48 (s, 1H), 3.64 (d, *J* = 8.6 Hz, 1H), 3.48–3.37 (m, 1H), 3.49–3.38 (m, 1H), 3.19 (t, *J* = 8.4 Hz, 1H), 3.13–2.96 (m, 3H), 2.38 (t, *J* = 7.1 Hz, 2H), 2.31–2.05 (m, 2H), 1.96–1.75 (m, 2H); ^13^C-NMR (101 MHz, DMSO-*d_6_*) *δ* 172.63, 171.60, 148.08, 144.30, 140.43, 139.10, 130.84, 129.88, 128.00, 120.76, 120.70, 120.29, 119.61, 116.47, 79.96, 79.02, 78.02, 72.97, 70.50, 61.44, 36.21, 35.08, 21.26; HRMS (ESI): Calcd. for [M-H]^−^ C_25_H_29_N_6_O_9_S^−^: 589.1795, Found 589.1792.

*N*^1^-(2-fluoro-4-(1-(4-sulfamoylphenyl)-1*H*-1,2,3-triazol-4-yl)phenyl)-*N*^5^-((2*R*,3*R*,4*S*,5*S*,6*R*)-3,4,5-trihydroxy-6-(hydroxymethyl)tetrahydro-2*H*-pyran-2-yl)glutaramide (**16j**): ^1^H-NMR (600 MHz, DMSO-*d_6_*) *δ* 10.13 (s), 9.36 (s), 8.18 (d, *J* = 8.2 Hz), 8.06 (d, *J* = 8.3 Hz), 7.88 (d, *J* = 7.9 Hz), 7.75 (d, *J* = 7.6 Hz), 7.55 (s), 5.01 (s), 4.90 (dd, *J* = 12.8, 4.3 Hz), 4.72 (t, *J* = 8.5 Hz), 4.51 (s), 3.64 (dd, *J* = 10.6, 4.3 Hz), 3.42 (d, *J* = 6.1 Hz), 3.18 (s), 3.07 (dd, *J* = 9.7, 4.7 Hz), 3.03–2.93 (m), 2.64–2.53 (m), 2.41–2.33 (m), 2.20 (ddd, *J* = 27.9, 14.0, 6.9 Hz), 1.89–1.78 (m); ^13^C-NMR (151 MHz, DMSO-*d_6_*) *δ* 172.73, 171.93, 154.87, 153.25, 146.89, 144.27, 139.03, 128.12, 127.29, 126.67, 126.59, 124.95, 121.72, 120.73, 120.53, 112.79, 112.65, 79.82, 78.91, 77.77, 72.74, 70.31, 61.28, 35.64, 35.01, 21.30; HRMS (ESI): Calcd. for [M-H]^−^ C_25_H_28_FN_6_O_9_S^−^: 607.1701, Found 607.1695.

*N*^1^-(2-chloro-4-(1-(4-sulfamoylphenyl)-1*H*-1,2,3-triazol-4-yl)phenyl)-*N*^5^-((2*R*,3*R*,4*S*,5*S*,6*R*)-3,4,5-trihydroxy-6-(hydroxymethyl)tetrahydro-2*H*-pyran-2-yl)glutaramide (**16k**): ^1^H-NMR (400 MHz, DMSO-*d_6_*) *δ* 12.21 (s, 1H), 9.25 (s, 1H), 8.26 (d, *J* = 9.1 Hz, 1H), 8.16 (q, *J* = 8.9 Hz, 4H), 7.77 (d, *J* = 1.8 Hz, 1H), 7.62 (dd, *J* = 8.4, 1.8 Hz, 1H), 6.90 (d, *J* = 8.4 Hz, 1H), 5.62 (s, 2H), 4.92 (s, 1H), 4.81 (d, *J* = 15.1 Hz, 2H), 4.68 (t, *J* = 9.0 Hz, 1H), 4.44 (s, 1H), 3.62 (d, *J* = 11.3 Hz, 1H), 3.40 (dd, *J* = 11.5, 4.2 Hz, 1H), 3.21–3.12 (m, 1H), 3.05 (dt, *J* = 17.8, 9.2 Hz, 3H), 2.28 (t, *J* = 7.3 Hz, 2H), 2.19–1.88 (m, 2H), 1.64 (p, *J* = 7.3 Hz, 2H); ^13^C-NMR (101 MHz, DMSO-*d_6_*) *δ* 172.32, 172.05, 147.84, 145.47, 140.38, 139.25, 130.08, 126.57, 125.57, 120.47, 119.14, 118.63, 117.78, 116.08, 79.91, 78.99, 78.02, 72.94, 70.50, 61.43, 35.21, 34.54, 19.96; HRMS (ESI): Calcd. for [M-H]^−^ C_25_H_28_ClN_6_O_9_S^−^: 623.1405, Found 623.1408.

*N*^1^-(2-methyl-4-(1-(4-sulfamoylphenyl)-1*H*-1,2,3-triazol-4-yl)phenyl)-*N*^5^-((2*R*,3*R*,4*S*,5*S*,6*R*)-3,4,5-trihydroxy-6-(hydroxymethyl)tetrahydro-2*H*-pyran-2-yl)glutaramide (**16l**): ^1^H-NMR (400 MHz, DMSO-*d_6_*) *δ* 9.31 (s), 8.17 (d, *J* = 8.7 Hz), 8.07 (d, *J* = 8.7 Hz), 7.81 (s), 7.74 (d, *J* = 9.6 Hz), 7.58 (d, *J* = 8.2 Hz), 4.74 (d, *J* = 9.0 Hz), 3.65 (d, *J* = 11.5 Hz), 3.42 (dd, *J* = 11.8, 5.5 Hz), 3.21 (t, *J* = 8.8 Hz), 3.14 (dd, *J* = 8.3, 6.6 Hz), 3.07 (dd, *J* = 19.1, 10.1 Hz), 2.41 (t, *J* = 7.0 Hz), 2.30 (s), 2.23 (dd, *J* = 12.3, 7.2 Hz), 1.92–1.80 (m); ^13^C-NMR (101 MHz, DMSO-*d_6_*) *δ* 172.67, 171.48, 147.93, 144.27, 139.12, 137.17, 132.42, 128.04, 127.69, 126.92, 125.80, 123.52, 120.59, 119.90, 79.99, 79.02, 78.03, 72.97, 70.52, 61.46, 35.74, 35.19, 21.57, 18.53; HRMS (ESI): Calcd. for [M-H]^−^ C_26_H_31_N_6_O_9_S^−^: 603.1951, Found 603.1947.

*N*^1^-(2-methoxy-4-(1-(4-sulfamoylphenyl)-1*H*-1,2,3-triazol-4-yl)phenyl)-*N*^5^-((2*R*,3*R*,4*S*,5*S*,6*R*)-3,4,5-trihydroxy-6-(hydroxymethyl)tetrahydro-2*H*-pyran-2-yl)glutaramide (**16m**): ^1^H-NMR (600 MHz, DMSO-*d_6_*) *δ* 9.41 (s, 1H), 9.18 (s, 1H), 8.34 (d, *J* = 9.1 Hz, 1H), 8.21–8.15 (m, 2H), 8.13 (d, *J* = 8.2 Hz, 1H), 8.09–8.04 (m, 2H), 7.60 (d, *J* = 1.7 Hz, 1H), 7.55 (s, 2H), 7.51 (dd, *J* = 8.2, 1.7 Hz, 1H), 4.98 (d, *J* = 4.4 Hz, 1H), 4.88 (dd, *J* = 10.1, 5.4 Hz, 2H), 4.73 (t, *J* = 9.1 Hz, 1H), 4.50 (t, *J* = 5.8 Hz, 1H), 3.95 (s, 3H), 3.68–3.59 (m, 1H), 3.41 (dt, *J* = 11.5, 5.7 Hz, 1H), 3.18 (td, *J* = 8.7, 3.8 Hz, 1H), 3.12–3.01 (m, 3H), 2.44 (t, *J* = 7.1 Hz, 2H), 2.27–2.08 (m, 2H), 1.82 (p, *J* = 7.5 Hz, 2H); ^13^C-NMR (151 MHz, DMSO-*d_6_*) *δ* 172.68, 171.72, 150.20, 148.07, 144.27, 139.09, 128.14, 128.06, 126.22, 122.48, 120.56, 119.95, 117.86, 108.44, 79.95, 79.02, 78.02, 72.95, 70.48, 61.41, 56.31, 36.01, 35.14, 21.48; HRMS (ESI): Calcd. for [M-H]^−^ C_26_H_31_N_6_O_10_S^−^: 619.1901, Found 619.1889.

*N*^1^-(2,6-difluoro-4-(1-(4-sulfamoylphenyl)-1*H*-1,2,3-triazol-4-yl)phenyl)-*N*^5^-((2*R*,3*R*,4*S*,5*S*,6*R*)-3,4,5-trihydroxy-6-(hydroxymethyl)tetrahydro-2*H*-pyran-2-yl)glutaramide (**16n**): ^1^H-NMR (600 MHz, DMSO-*d_6_*) *δ* 12.25 (s), 9.31 (s), 8.28 (d, *J* = 9.1 Hz), 8.21–8.03 (m), 7.49 (dd, *J* = 7.4, 2.1 Hz), 5.51 (s), 4.95 (d, *J* = 4.4 Hz), 4.86 (d, *J* = 5.2 Hz), 4.82 (d, *J* = 5.0 Hz), 4.68 (t, *J* = 9.1 Hz), 4.47 (t, *J* = 5.4 Hz), 3.62 (dd, *J* = 11.4, 3.2 Hz), 3.45–3.36 (m), 3.15 (td, *J* = 8.7, 4.1 Hz), 3.11–3.05 (m), 3.02 (td, *J* = 9.1, 5.1 Hz), 2.27 (t, *J* = 7.4 Hz), 2.16–1.97 (m), 1.63 (p, *J* = 7.4 Hz); ^13^C-NMR (101 MHz, DMSO-*d_6_*) *δ* 172.64, 172.21, 152.93, 152.83, 150.56, 150.46, 147.17, 140.19, 139.58, 130.06, 126.36, 126.19, 126.02, 120.62, 119.42, 116.99, 108.88, 108.80, 108.65, 79.78, 78.82, 77.70, 72.67, 70.33, 61.30, 35.26, 34.53, 20.04; HRMS (ESI): Calcd. for [M-H]^−^ C_25_H_27_F_2_N_6_O_9_S^−^: 625.1607, Found 625.1601.

*N*^1^-(2-nitro-4-(1-(4-sulfamoylphenyl)-1*H*-1,2,3-triazol-4-yl)phenyl)-*N*^5^-((2*R*,3*R*,4*S*,5*S*,6*R*)-3,4,5-trihydroxy-6-(hydroxymethyl)tetrahydro-2*H*-pyran-2-yl)glutaramide (**16o**): ^1^H-NMR (400 MHz, DMSO-*d_6_*) *δ* 12.32 (s, 1H), 9.38 (s, 1H), 8.55 (d, *J* = 1.7 Hz, 1H), 8.24 (d, *J* = 9.0 Hz, 1H), 8.08 (q, *J* = 8.6 Hz, 4H), 7.98 (dd, *J* = 8.8, 1.7 Hz, 1H), 7.64 (s, 2H), 7.17 (d, *J* = 8.8 Hz, 1H), 4.92 (d, *J* = 3.1 Hz, 1H), 4.83 (d, *J* = 3.8 Hz, 2H), 4.68 (t, *J* = 9.0 Hz, 1H), 4.46 (s, 1H), 3.62 (d, *J* = 11.4 Hz, 1H), 3.39 (dd, *J* = 12.2, 3.5 Hz, 2H), 3.15 (d, *J* = 12.8 Hz, 2H), 3.10–2.93 (m, 4H), 2.16 (s, 2H), 2.06 (dd, *J* = 13.1, 7.1 Hz, 2H), 1.70–1.51 (m, 2H); ^13^C-NMR (101 MHz, DMSO-*d_6_*) *δ* 172.44, 146.29, 145.75, 141.75, 138.69, 132.89, 130.13, 129.09, 121.66, 119.91, 119.66, 118.83, 117.50, 79.24, 78.25, 77.14, 72.09, 69.74, 60.72, 36.07, 34.37, 20.29; HRMS (ESI): Calcd. for [M-H]^−^ C_25_H_28_N_7_O_11_S^−^: 634.1646, Found 634.1639.

### 3.2. CA Inhibition Assay

Carbonic anhydrase has the ability to catalyse the hydrolysis of 4-NPA. According to the previously reported method, the Perkin Elmer Envision 2104 plate reader was used to monitor the hydrolysis rate spectrophotometrically at 405 nm [29,30]. The inhibitory activities of these compounds were investigated with the inhibitor AZM as standard. More detailed operations and specific parameters are as reported earlier [30].

### 3.3. CCK-8 Assay In Vitro

Cell viability was tested by CCK-8 assay. The MDA-MB-231, HT-29 and MG-63 cell lines were provided by KeyGEN BioTECH Ltd. (Nanjing, Jiangsu, China). The cancer cell suspension was prepared to 5.0 × 10^4^ cells/mL. After adding 100 μL cell suspension into each well of 96-well plates, the 96-well plates were placed in the incubator at 37 °C and 5% CO_2_ for 24 h. The compounds were diluted to the required concentration with the medium, and 100 μL of the corresponding medium was added to each well. Meanwhile, the negative control group was set up. The plates were incubated in normoxia or hypoxia for 48 h. The CCK-8 solution (10 μL) was added to each well, and the cells were incubated for another 3 h. After removing the bubbles in each well, cell viability was assessed by measuring the absorbance at 450 nm wavelength using a microplate reader. The hypoxia atmosphere consisted of 0.5% O_2_, 5% CO_2_, and 94.5% N_2_. The normoxia atmosphere was air.

### 3.4. Measurement of Extracellular pH

The changes in extracellular pH were evaluated according to previously published procedures [31]. Cancer cells were grown in the medium containing 10% FBS and incubated in 6-well plates for 12 h. After removing the medium, we added fresh medium to the 6-well plates and incubated them for 48 h under hypoxia or normoxia. Then, we measured the pH of the culture medium immediately after collection. The tested compounds were dissolved in DMSO and diluted to the desired concentration. After the addition of tested compounds, the same procedure was carried out.

### 3.5. Cell Migration Assay

MDA-MB-231 cells (1 × 10^5^ cells/well) were cultured in 6-well plates and wounded by scratching with a pipette tip. The wounded cell monolayers were washed three times with PBS and incubated with serum-free medium. Then, cells were treated with AZM and **16a** respectively followed by a 72 h incubation under hypoxic condition, and they were photographed at 0, 24, 48, and 72 h with an inverted microscope. 

### 3.6. Molecular Docking Studies

The CA IX crystal structure (PDB code: 5FL4) was downloaded from the protein data bank (https://www.rcsb.org/, accessed on 9 November 2021) and prepared with the Protein Preparation Wizard in the Schrödinger suite. The preparation of protein structure included adding bond orders, adding hydrogen atoms, deleting water molecules, and producing appropriate protonation states. The protonation and tautomeric states of Asp, Lys, and His were assigned at pH 7.4 state. The co-crystal ligand of 5FL4 was separated from the crystal structure. The selected inhibitors were prepared by using LigPrep from the Schrödinger suite with the OPLS_2005 force field. The structure of the inhibitor has also been modified by adding all hydrogen atoms, checking the bond sequence and atom type.

Receptor grids were generated before docking with the activity site determined by the literature. The prepared protein–ligand complex was imported into Glide 9.7, which defined it as the receptor structure with a size box (20 Å × 20 Å × 20 Å) [32,33]. The grid of the CA IX crystal structure was generated based on the OPLS_2005 force field. The standard precision (SP) mode was set for docking studies [34].

The standard descriptors protocol in the DS (version 3.0, Accelrys Inc., San Diego, CA, USA) was used to assess the ADME/T profile of the selected compounds. With the help of the ADME/T module, various parameters including TPSA are used to predict the ADME/T characteristics of specified compounds [35].

## 4. Conclusions

In this work, 15 novel saccharide-modified carbonic anhydrase inhibitors, **16a–o,** were designed and synthesised via the “tail approach”. Saccharide-modified compounds **16a–o** and key intermediates **14a–h** were evaluated for their enzymatic activity against tumour-associated isoforms CA IX and XII and the common off-target isoform CA II. The results showed that all the saccharide-modified compounds had inhibitory activity against *h*CA II, IX, and XII, with IC_50_ values ranging from 13.3 to 177.2 nM, 51.6 to 543.9.6 nM, and 135.3 to 255.9 nM, respectively. Afterwards, the series of compounds were tested at the cellular level to investigate their effects on the cell viabilities of the MDA-MB-231, HT-29, and MG-63 cell lines. Compounds **16a**, **16b**, and **16e** could reduce cell viability at high concentrations, and this effect was particularly obvious under hypoxic conditions. Moreover, we found that compounds **16a** and **16b** could significantly increase the extracellular pH of tumour cells and transform the microenvironment from acidic to alkaline. In addition, in the cell migration experiment, **16a** exhibited a more apparent antimigration effect than the positive control AZM, and we predicted the pharmacokinetic properties of the synthesised compounds using the SwissADME website to find compounds that may have good pharmacokinetic properties (detailed results are provided in the Tables S7–S13 of supplementary information) [36]. Based on the above test results, we believe that modification of the glycosyl tail of targeted CA IX inhibitors is an effective strategy worthy of further study.

## Figures and Tables

**Table 1:** The IC_50_ of the compounds **14a–h** and **16a–o** against *h*CA II, IX, and XII.

Compound	Structure	IC_50_ (nM)			TPSA (Å^2^)
		*h*CA II	*h*CA IX	*h*CA XII	
**14a**	R=H	19.3	54.4	n.t.	114.1
**14b**	R=H *	12.3	50.4	n.t.	114.1
**14c**	R=2-F	15.7	40.4	n.t.	114.1
**14d**	R=2-Cl	39.9	71.0	n.t.	114.1
**14e**	R=2-CH_3_	37.9	110.9	n.t.	114.1
**14f**	R=2-OCH_3_	100.0	120.2	n.t.	123.4
**14g**	R=2-,6-F	87.0	107.3	n.t.	114.1
**14h**	R=2-NO_2_	102.0	58.2	n.t.	165.9
**16a**	n = 2, R=H	27.7	51.6	135.3	236.5
**16b**	n = 2, R=H *	13.3	86.6	255.9	236.5
**16c**	n = 2, R=2-F	70.2	150.0	n.t.	236.5
**16d**	n = 2, R=2-Cl	90.8	100.3	n.t.	236.5
**16e**	n = 2, R=2-CH_3_	41.6	84.7	162.1	236.5
**16f**	n = 2, R=2-OCH_3_	111.7	329.3	n.t.	245.7
**16g**	n = 2, R=2-,6-F	149.2	543.9	n.t.	236.5
**16h**	n = 3, R=H	26.0	270.3	n.t.	236.5
**16i**	n = 3, R=H *	10.1	299.7	n.t.	236.5
**16j**	n = 3, R=2-F	88.4	230.1	n.t.	236.5
**16k**	n = 3, R=2-Cl	91.0	290.4	n.t.	236.5
**16l**	n = 3, R=2-CH_3_	25.6	160.9	n.t.	236.5
**16m**	n = 3, R=2-OCH_3_	88.1	99.6	146.1	245.7
**16n**	n = 3, R=2-,6-F	177.2	351.6	n.t.	236.5
**16o**	n = 3, R=2-NO_2_	129.2	216.1	n.t.	288.3
AZM		13.0	50.6	38.2	113.8

Notes: * Amino and triazole groups are in positions 1–3; Relative errors were in the range of 5–10% of reported values, which were calculated from three independent assays.

## Supplementary Materials

The supplementary materials are available online https://www.mdpi.com/article/10.3390/ijms222413610/s1.
