# Supplementary material for: Synthesis, Molecular Docking Analysis, and Biological Evaluations of Saccharide-Modified Sulfonamides as Carbonic Anhydrase IX Inhibitors
Source: Int J Mol Sci. 2021 Dec 19;22(24):13610. doi: 10.3390/ijms222413610 (PMC8704611; doi:10.3390/ijms222413610)
Supplement: Supplementary file 1 [file ijms-22-13610-s001.zip › ijms-1473793- supplementary revised.pdf]

Supporting Information

Synthesis, Molecular Docking Analysis and Biological Evaluations of Saccharide-modified Sulfonamides as Carbonic Anhydrase IX Inhibitors

Zuo-Peng Zhang,[§] Hua-Li Yang, [§,*] Ye Zhong, Yue-Qing Wang,

Jian Wang, Mao-Sheng Cheng, Yang Liu*

Table of Contents

The modes of binding for co-crystal structure 5FL4 in CA IX..................2

Cell viability test results 3

Extracellular pH measurement results..3

In silico pharmacokinetic study of the synthesized compounds ....4~10

^{1}H-NMR and ^{13}C-NMR Spectra .... 11~51

Table S1. Binding energy, hydrogen bonding interacting residues, H-binding distance, hydrophobic interacting residues of 16a, 16g and 9FK.

Compound	binding energy	hydrogen bonding interacting residues	H-binding distance (Å)	hydrophobic interacting residues
16a	-8.37	Gln92<br>Arg129<br>Asp131<br>Thr200	1.9108<br>1.9173<br>1.9870<br>1.9720	His96<br>Val130<br>Leu134<br>Leu199
16g	-6.49	Val20<br>Thr200	2.1741<br>1.6311	His94<br>Val121<br>Val130<br>Leu199
9FK	-7.83	Gln92<br>His119<br>His119<br>Thr200	2.8656<br>2.8418<br>2.7080<br>2.0466	His94<br>His96<br>Val130<br>Leu134<br>Leu199<br>Trp210

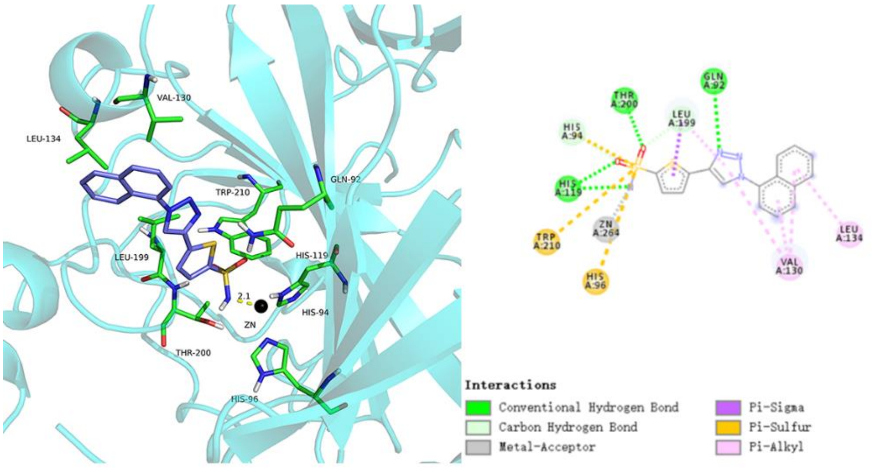

Figure S1. The modes of binding for co-crystal structure 5FL4 in CA IX.

Receptor grids were generated before docking with activity site determined by literature. The prepared protein-ligand complex was imported into Glide 9.7, which defined it as the receptor structure with size box (20 Å × 20 Å × 20 Å), the coordinates of the grid were X: 15.65 Å, Y:-27.63 Å, Z:59.53 Å in 5fl4. The grid of the CA IX crystal structure was generated based on the OPLS_2005 force field. The standard precision (SP) mode was set for docking studies.

Table S2. Compounds **16a**, **16b**, **16e** and AZM on the cell viability of HT-29 at the concentration of 400 μM.

Compounds	Normoxic condition (%)	Hypoxic condition (%)
16a	83.2967±2.0924	82.6309±1.9157
16b	89.6703±0.5539	81.0983±2.4266
16e	91.4547±2.6427	87.7241±1.7931
AZM	91.6947±2.5866	89.5262±0.1613

Table S3. Compounds **16a**, **16b**, **16e** and AZM on the cell viability of MDA-MB-231 at the concentration of 400 μM..

Compounds	Normoxic condition (%)	Hypoxic condition (%)
16a	88.8532±1.7149	85.1319±1.6787
16b	90.4609±0.9646	86.4508±1.9185
16e	87.2156±2.2752	90.6250±2.7644
AZM	96.1539±2.2837	85.1897±1.9584

Table S4. Compounds **16b** and AZM on the cell viability of MG-63 at the concentration of 800 μM..

Compounds	Normoxic condition (%)	Hypoxic condition (%)
16b	81.6287±3.3416	76.6021±3.5111
AZM	87.0634±1.9405	81.6880±1.1688

Table S5. Extracellular pH measurement of MDA-MB-231 under normoxic and hypoxic conditions.

Compounds	Normoxic condition	Hypoxic condition
16a (0.1 mM)	7.04	6.9
16a (1.0 mM)	7.21	7.31
16b (0.1 mM)	7.02	6.88
16b (1.0 mM)	7.20	7.20
AZM (0.1 mM)	7.05	6.97
AZM (1.0 mM)	7.23	7.42
Control	7.06	6.84

Table S6. Extracellular pH measurement of HT-29 under normoxic and hypoxic conditions.

Compounds	Normoxic condition	Hypoxic condition
16a (0.1 mM)	6.97	6.88
16a (1.0 mM)	7.13	7.26
16b (0.1 mM)	6.96	6.82
16b (1.0 mM)	7.15	7.27
AZM (0.1 mM)	6.97	6.90
AZM (1.0 mM)	7.26	7.28
Control	7.02	6.81

In silico pharmacokinetic study of the synthesized compounds

This section comprises clean molecular and physicochemical characteristics like molecular formula, molecular weight, number of heavy atoms, number of aromatic heavy atoms, fraction csp3, number of rotatable bonds, number of H-bond acceptors, number of H-bond donors, molar refractivity as shown in **Table S6** respectively.

Table S7. Physicochemical properties prediction of the compounds by SwissADME

Molecule	Formula	MW	Fraction Csp3	#HBA	#HBD	MR
16a	C24H28N6O9S	576.58	0.33	12	7	137.18
16b	C24H28N6O9S	576.58	0.33	12	7	137.18
16c	C24H27FN6O9S	594.57	0.33	13	7	137.14
16d	C24H27ClN6O9S	611.02	0.33	12	7	142.19
16e	C25H30N6O9S	590.61	0.36	12	7	142.14
16f	C25H30N6O10S	606.60	0.36	13	7	143.67
16g	C24H26F2N6O9S	612.56	0.33	14	7	137.09
16h	C25H30N6O9S	590.61	0.36	12	7	141.98
16i	C25H30N6O9S	590.61	0.36	12	7	141.98
16j	C25H29FN6O9S	608.60	0.36	13	7	141.94
16k	C25H29ClN6O9S	625.05	0.36	12	7	146.99
16l	C26H32N6O9S	604.63	0.38	12	7	146.95
16m	C26H32N6O10S	620.63	0.38	13	7	148.48
16n	C25H28F2N6O9S	626.59	0.36	14	7	141.90
16o	C25H29N7O11S	635.60	0.36	14	7	150.81

Lipophilicity is a paramount parameter in drug discovery and design on grounds that it complements the single most informational and successful physicochemical property in medicinal chemistry. It is experimentally demonstrated as partition coefficients (log P) or as distribution coefficients (log D). Log P portrays partition equilibrium of an un-ionized solute amidst water and an immiscible organic solvent. The larger the log P values correspond the greater the lipophilicity. To evaluate the lipophilicity character in a compound, Swiss ADME provides five freely available models i.e., XLOGP3, WLOGP, MLOGP, SILICOS-IT, and iLOGP respectively. XLOGP3, an atomistic accost including corrective factors and knowledge-based library; WLOGP, application of purely atomistic method stationed on the fragmental system; MLOGP, an archetype of the topological method suggested on a linear relationship with implemented 7 topological descriptors; iLOGP, a physics-based method lean on free energies of solvation in n-octanol and water

calculated by the generalized-born and solvent accessible surface area (GB/SA) model; Consensus log P o/w is an arithmetic mean of the values predicted by the five proposed methods as shown in **Table S7**. The consensus Log P of all synthetic compounds was less than 0, showing poor lipophilicity.

Table S8. Lipophilicity prediction of the compounds by SwissADME

Molecule	iLOGP	XLOGP3	WLOGP	MLOGP	Silicos-IT Log P	Consensus Log P
16a	1.70	-2.20	0.89	-1.95	-2.51	-1.17
16b	1.33	-2.20	0.89	-1.95	-2.51	-1.25
16c	-0.52	-2.10	-0.33	-1.58	-2.08	-1.32
16d	1.27	-1.57	-0.24	-1.47	-1.86	-0.77
16e	1.98	-1.83	-0.59	-1.74	-1.98	-0.83
16f	0.90	-2.23	-0.88	-2.21	-2.42	-1.37
16g	1.74	-2.00	0.23	-1.20	-1.64	-0.58
16h	2.46	-1.84	-0.50	-1.74	-2.11	-0.75
16i	1.46	-1.84	-0.50	-1.74	-2.11	-0.95
16j	2.54	-1.74	0.06	-1.37	-1.67	-0.44
16k	1.79	-1.21	0.15	-1.27	-1.45	-0.40
16l	1.96	-1.48	-0.20	-1.54	-1.57	-0.56
16m	1.82	-1.87	-0.49	-2.00	-2.02	-0.91
16n	0.32	-1.64	0.62	-1.00	-1.23	-0.59
16o	1.30	-1.46	-0.60	-2.44	-4.22	-1.48

The breadth of solubility is measured as the saturation concentration whereupon adding more solute does not increase its concentration in the solution. A drug is considered highly soluble when the highest dose strength is soluble in 250 mL or less of aqueous media over the pH range of 1 to 7.5. Two topological approaches included in Swiss ADME to predict water solubility, the first one is the application of ESOL model (Solubility class: Log S Scale: Insoluble<-10 poorly<-6, moderately<-4 soluble<-2 very<0<highly) and the second one is adapted from Ali et al, 2012 (Solubility class: Log S Scale: Insoluble<-10 poorly<-6, moderately<-4 soluble<-2very<0<highly). Both differ from the fundamental general solubility equation since they avoid the melting point parameter but the linear correlation between predicted and experimental values were strong as shown in **Table S8**. The third predictor of Swiss ADME was developed by SILICOS-IT (Solubility class: Log S Scale: Insoluble<-10 poorly<-6, moderately<-4 soluble<-2 very<0<highly). Compared with lipophilicity, this series of saccharide-modified compounds showed excellent water solubility.

Table S9. Solubility prediction of the compounds by SwissADME

Molecule	ESOL Log S	ESOL Class	Ali Log S	Ali Class
16a	-1.62	Very soluble	-2.47	Soluble
16b	-1.62	Very soluble	-2.47	Soluble
16c	-1.78	Very soluble	-2.57	Soluble
16d	-2.22	Soluble	-3.12	Soluble
16e	-1.93	Very soluble	-2.85	Soluble
16f	-1.70	Very soluble	-2.63	Soluble
16g	-1.95	Very soluble	-2.68	Soluble
16h	-1.86	Very soluble	-2.84	Soluble
16i	-1.86	Very soluble	-2.84	Soluble
16j	-2.02	Soluble	-2.95	Soluble
16k	-2.46	Soluble	-3.50	Soluble
16l	-2.16	Soluble	-3.22	Soluble
16m	-1.94	Very soluble	-3.00	Soluble
16n	-2.19	Soluble	-3.05	Soluble
16o	-2.29	Soluble	-4.20	Moderately soluble

The delineation exists in a region of agreeable properties for GI absorption on a plot of two computed descriptors; ALOGP versus PSA respectively. The region most populated by well-absorbed molecules is elliptical, it was called Egan egg, which is used to assess the predictive power of the model for GI passive absorption and prediction for brain access by passive diffusion to finally lay the BOILED-Egg (Brain or Intestinal L Estimate D permeation predictive model). The BOILED-Egg model produces a rapid, spontaneous, efficiently imitate yet boisterous method to forecast the passive GI absorption helpful for drug discovery and development. The GI absorption and BBB permeant of this series of compounds are shown in **Table S9**. Cytochrome p450 (CYP) isoenzymes biotransform more than 50-90% of therapeutic molecules from its five major isoforms (CYP1A2, CYP3A4, CYP2C9, CYP2C19, CYP2D6). P-gp is broadly dispersed in intestinal epithelium which pumps xenobiotics back into the intestinal lumen and from the capillary endothelial cells of the brain back into the capillaries. SwissADME adopts a support vector machine algorithm (SVM) for the datasets of known substrates/non- substrates or inhibitors/non-inhibitors for binary classification. The resultant molecule will return “Yes” or “No” if the molecule under investigation is expected to be a substrate for both P-gp and CYP as shown in **Table S10** respectively. The prediction shows that this series of compounds have little risk of

CYP enzyme inhibition.

Table S10. Pharmacokinetics prediction of the compounds by SwissADME

Molecule	Silicos-IT LogSw	Silicos-IT class	GI absorption	BBB permeant	Pgp substrate	log Kp (cm/s)
16a	-3.26	Soluble	Low	No	No	-11.38
16b	-3.26	Soluble	Low	No	No	-11.38
16c	-3.51	Soluble	Low	No	No	-11.42
16d	-3.83	Soluble	Low	No	No	-11.14
16e	-3.63	Soluble	Low	No	No	-11.20
16f	-3.35	Soluble	Low	No	No	-11.58
16g	-3.77	Soluble	Low	No	No	-11.46
16h	-3.65	Soluble	Low	No	No	-11.21
16i	-3.65	Soluble	Low	No	No	-11.21
16j	-3.90	Soluble	Low	No	Yse	-11.25
16k	-4.21	Moderately soluble	Low	No	No	-10.97
16l	-4.01	Moderately soluble	Low	No	No	-11.04
16m	-3.73	Soluble	Low	No	No	-11.41
16n	-4.16	Moderately soluble	Low	No	No	-11.29
16o	-2.96	Sluble	Low	No	Yes	-11.21

Table S11. CYP inhibition prediction of the compounds by SwissADME

Molecule	CYP1A2 inhibitor	CYP2C19 inhibitor	CYP2C9 inhibitor	CYP2D6 inhibitor	CYP3A4 inhibitor
16a	No	No	No	No	No
16b	No	No	No	No	No
16c	No	No	No	No	No

16d	No	No	No	No	No
16e	No	No	No	No	No
16f	No	No	No	No	No
16g	No	No	No	No	No
16h	No	No	No	No	No
16i	No	No	No	No	No
16j	No	No	No	No	No
16k	No	No	No	No	No
16l	No	No	No	No	No
16m	No	No	No	No	No
16n	No	No	No	No	No
16o	No	No	No	No	No

SwissADME performs filtering of chemical libraries to exclude molecules with peculiarities incompatible with an acceptable pharmacokinetic profile with five disparate ruled-based filters elemental from considerable Pharma companies intended to improve the condition of proprietary chemical collections. The Lipinski filter (Pfizer) is the pioneer rule of five that characterizes small molecules based on physicochemical property profiles which include Molecular Weight (MW) less than 500, MLOGP ≤ 4.15, N or O ≤ 10, NH or OH ≤ 5. Lipinski considers stringently that all nitrogen and oxygen as H-bond acceptors and all nitrogen and oxygens with at least one hydrogen as H-bond donors. Besides, aliphatic fluorine's are acceptors and alanine nitrogen are neither donors nor acceptors (Lipinski et al., 2001). The Ghose filter (Amgen) describes small molecules stationed on the physicochemical property, existence of functional groups, and substructures. The qualifying range including molecular weight is between 160 and 480 Da, WlogP is between -0.4 to 5.6, molar refractivity (MR) is between (40 to 130) for the total number of atoms. The qualifying range is between 20 and 70 atoms in a small molecule. Veber filter (GSK filter) model symbolizes molecules as drug-like if they have ≤ 10 rotatable bonds and a TPSA equal to or less than 140 Å2 with 12 or fewer H-bond donors and acceptors. Compounds with these properties will have good oral bioavailability, reduced TPSA correlates increased permeation rate, increased rotatable bond counts has a negative effect on the permeation rate. Egan filter (Pharmacia filter) anticipates drug absorption depends on processes involved in the membrane permeability of a small molecule. This model symbolizes molecule as a drug like if they have WLOGP ≤ 5.88 and TPSA ≤ 131.6 respectively. The Egan computational model for human passive intestinal absorption (HIA) of small molecule accounts for active transport and efflux mechanisms and is therefore robust in predicting absorption of drugs. Muegge filter (Bayer filter) is a self-reliant Pharmacophore point filter that segregates drug-like and nondrug-like molecules. This model symbolizes molecule as a drug like if they have a molecular weight between 200 to 600 Da, XLOGP between -2 and 5, TPSA ≤ 150, Number of rings ≤ 7, Number of carbon atoms > 4, number of heteroatoms > 1, number of rotatable bonds ≤ 15, H-bond acceptor ≤ 10, H-bond donor ≤ 5 respectively. Abbott

bioavailability score seeks to predicts the probability of a compound to have at least 10% oral bioavailability in rat or measurable Caco-2 permeability which predicts the probability of a compound to have F>10% based on the predominant charge at biological pH in a rat model. It focuses on the fast screening of chemical libraries to select the best molecules to be synthesized as shown in **Table S11**.

Table S12. Drug likeness prediction of the compounds by SwissADME

Molecule	Lipinski #violations	Ghose #violations	Veber #violations	Egan #violations	Muegge #violations	Bioavailability Score
16a	3	3	2	1	4	0.17
16b	3	3	2	1	4	0.17
16c	3	2	2	1	4	0.17
16d	3	2	2	1	4	0.17
16e	3	4	2	1	3	0.17
16f	3	4	2	1	5	0.17
16g	3	2	2	1	4	0.17
16h	3	4	2	1	3	0.17
16i	3	4	2	1	3	0.17
16j	3	3	2	1	4	0.17
16k	3	3	2	1	4	0.17
16l	3	3	2	1	4	0.17
16m	3	4	2	1	4	0.17
16n	3	3	2	1	4	0.17
16o	3	4	2	1	4	0.17

These section aims to bolster medicinal chemists in their daily drug discovery endeavors. PAINS (Pan Assay Interference Compounds or frequent hitters or promiscuous compounds) are the molecules that show potent response in assays irrespective of the protein targets, notably such compounds are reported to be active in many different assays, which can be considered as potential starting points for further exploration. SwissADME returns warnings if such moieties are found in the molecule under evaluation. In other models, Brenk considers compounds that are smaller and less hydrophobic and not those defined by "Lipinski's rule of 5" to widen opportunities for lead optimization. This was after the exclusion of compounds with potentially mutagenic, reactive, and unfavorable groups such as nitro groups, sulfates, phosphates, 2-halopyridines, and thiols. Brenk model restricts the ClogP/ClogD to between 0 and 4, the number of hydrogen-bond donors and acceptors to fewer than 4 and 7, respectively, and the number of heavy atoms to between 10 and 27 respectively. Additionally, only compounds with

limited complexity defined as fewer than 8 rotatable bonds, fewer than 5 ring systems, and no ring systems with more than 2 fused rings are considered medicinal. The concept of lead likeness is designed to provide leads with tremendous affinity in high throughput screening (HTS) that allows for the exploitation of additional interactions in the lead optimization phase. Leads are exposed to chemical modifications that will most likely decrease the size and increase lipophilicity which is less hydrophobic than drug-like molecules. Lead optimization has been done by a rule-based method consisting of molecules with molecular weight in between 100 and 350 Da, ClogP between 1 and 3.0, and are greatly considered as superior to those of drug-like compounds and therefore lead-like as shown in **Table S12.** The MW of this series of compounds is greater than 350 and the number of rotatable bonds is more than 7, which is contrary to leadlikeness.

Table S13. Medicinal chemistry prediction of the compounds by SwissADME

Molecule	PAINS #alerts	Brenk #alerts	Leadlikeness #violations	Synthetic Accessibility
16a	0	0	2	5.30
16b	0	0	2	5.32
16c	0	0	2	5.33
16d	0	0	2	5.34
16e	0	0	2	5.48
16f	0	0	2	5.49
16g	0	0	2	5.34
16h	0	0	2	5.41
16i	0	0	2	5.43
16j	0	0	2	5.44
16k	0	0	2	5.45
16l	0	0	2	5.58
16m	0	0	2	5.60
16n	0	0	2	5.45
16o	0	2	2	5.63

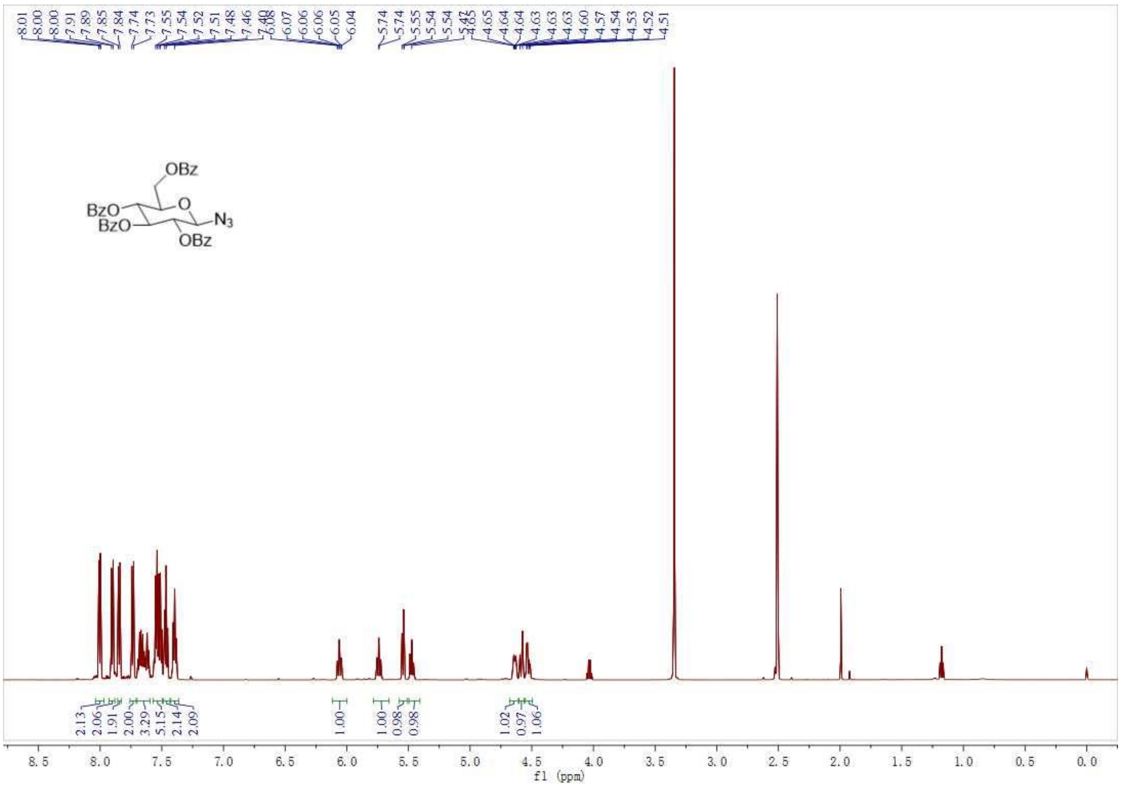

Figure S2. The ^{1}H-NMR of intermediate 4

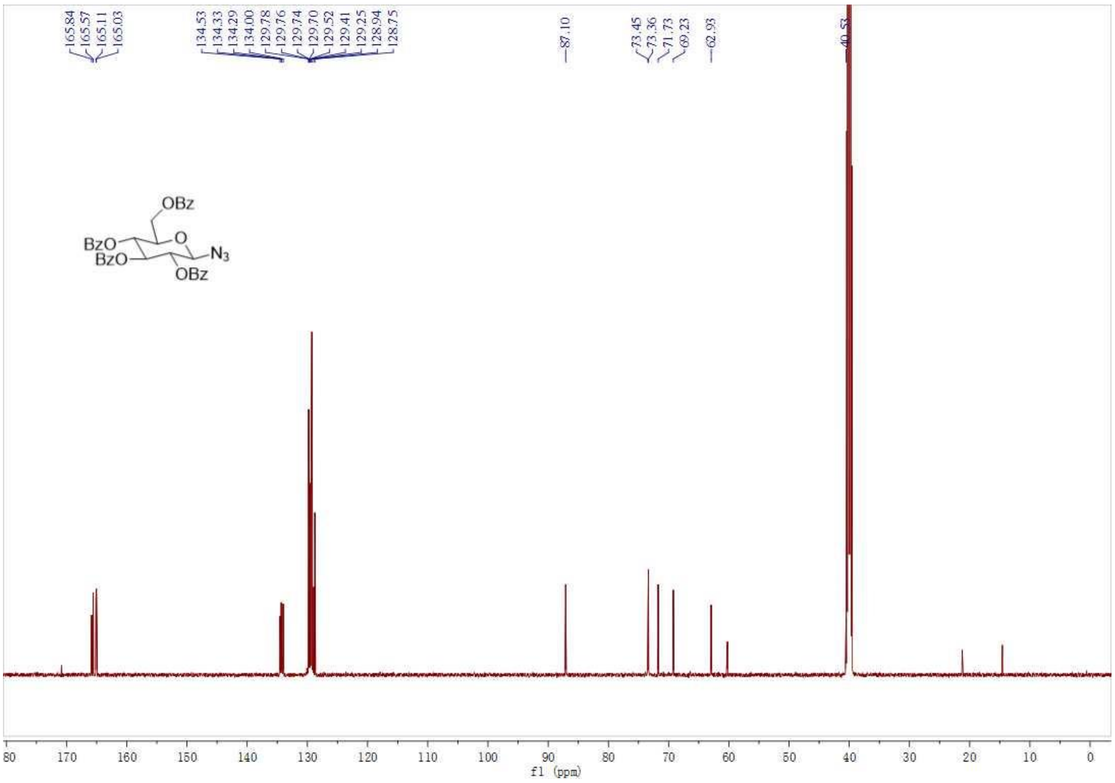

Figure S3. The ^{13}C-NMR of intermediate 4

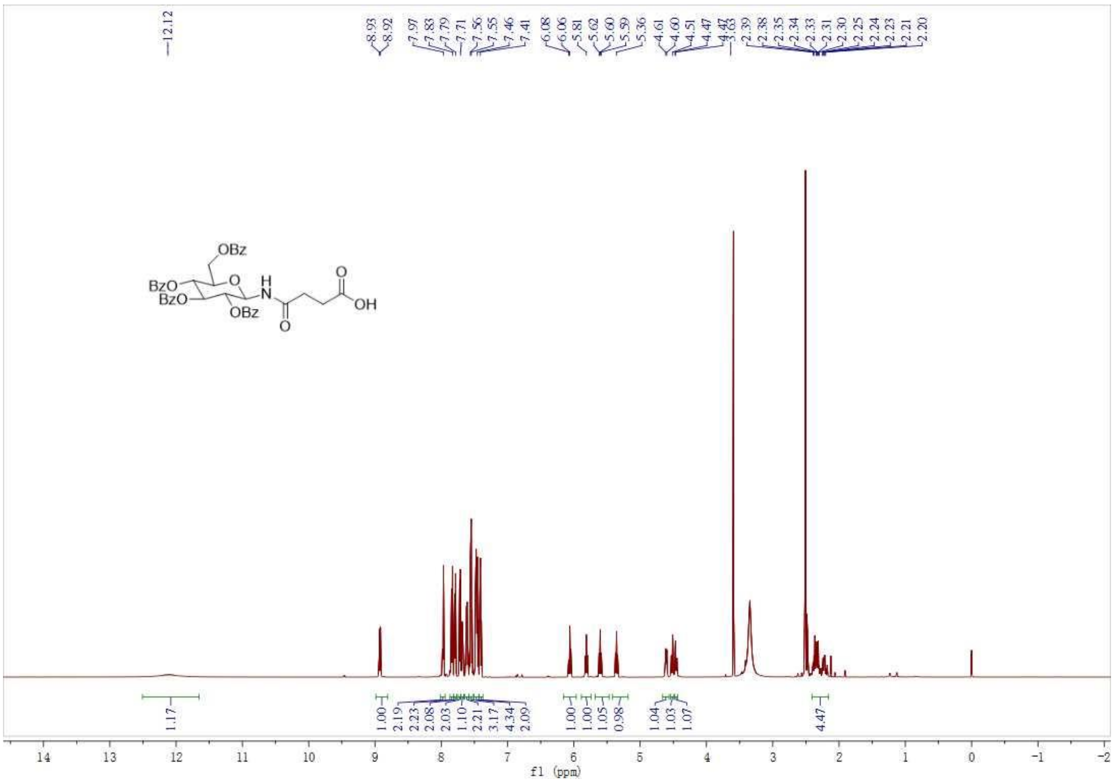

Figure S4. The ^{1}H-NMR of intermediate 5

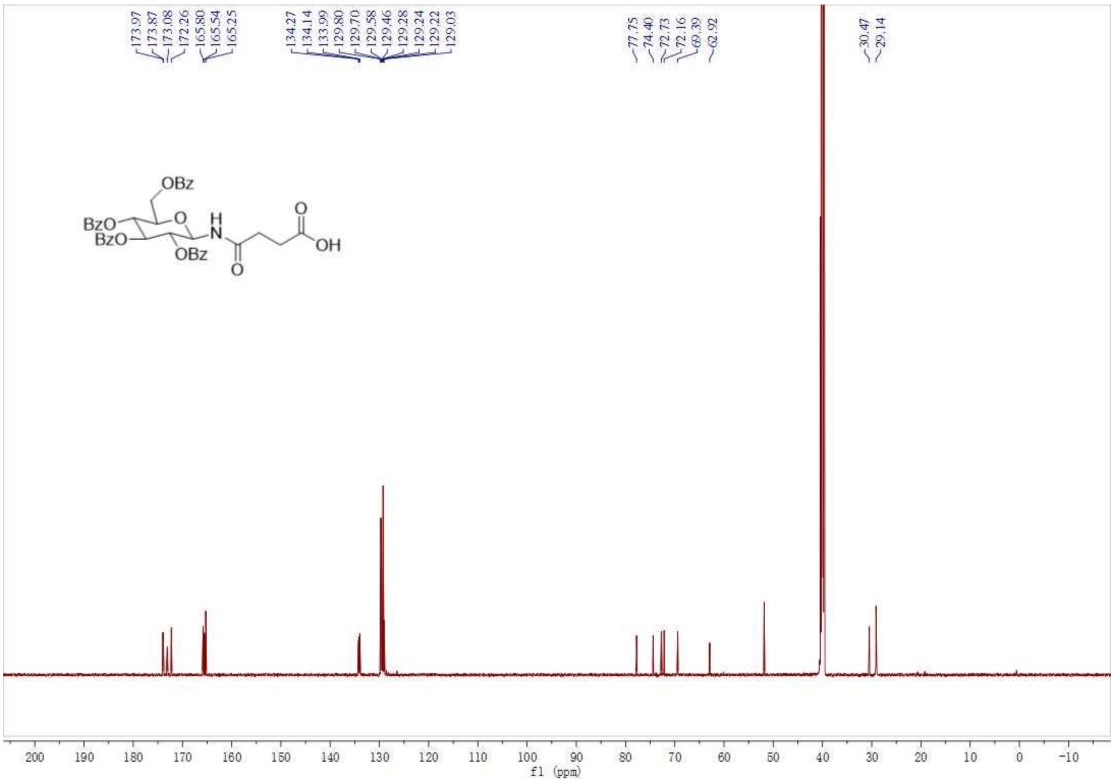

Figure S5. The ^{13}C-NMR of intermediate 5

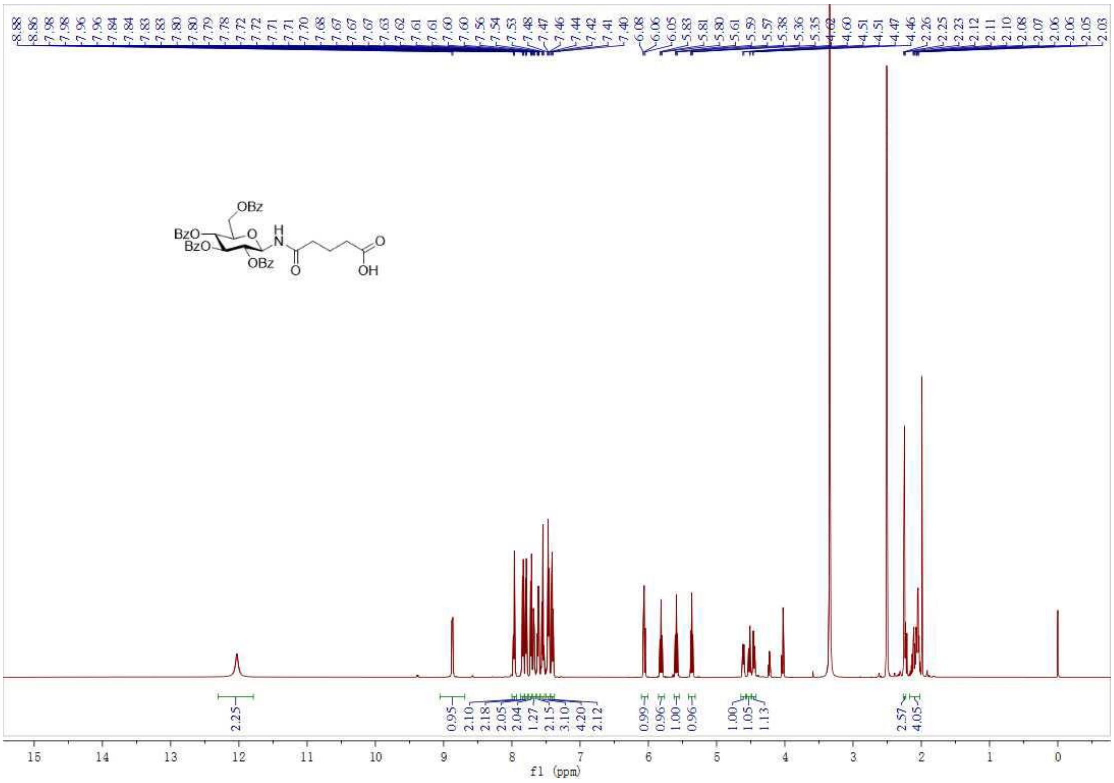

Figure S6. The ^{1}H-NMR of intermediate 6

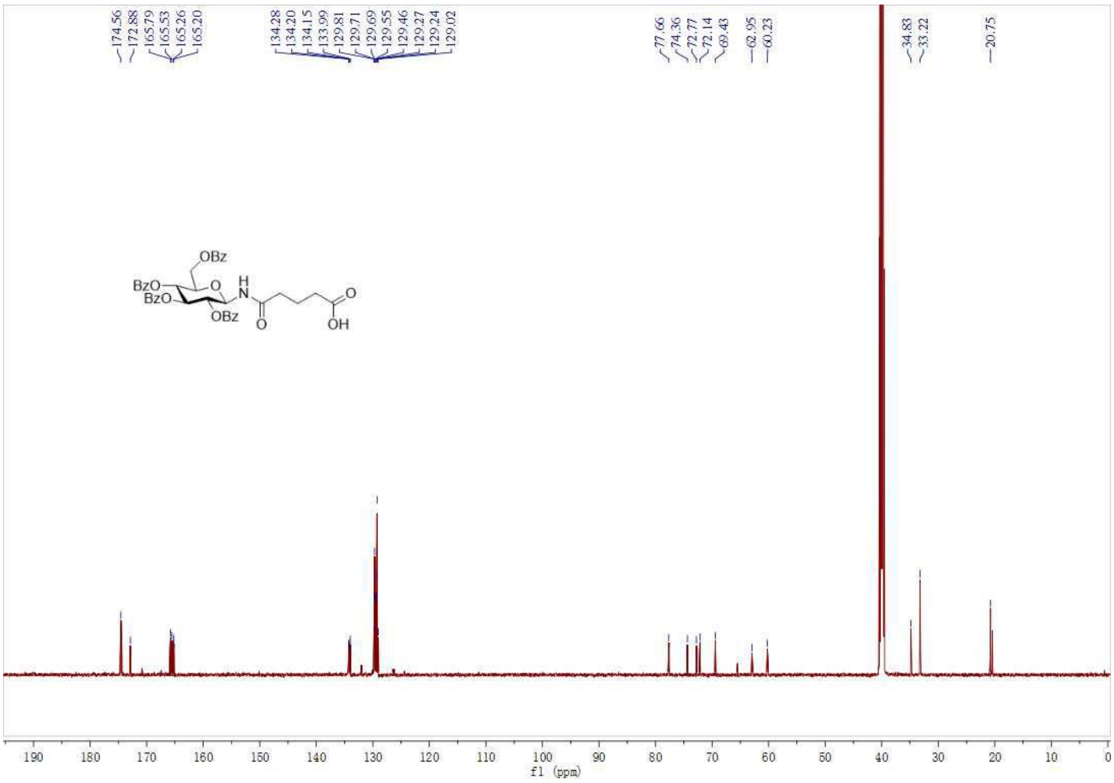

Figure S7. The ^{13}C-NMR of intermediate 6

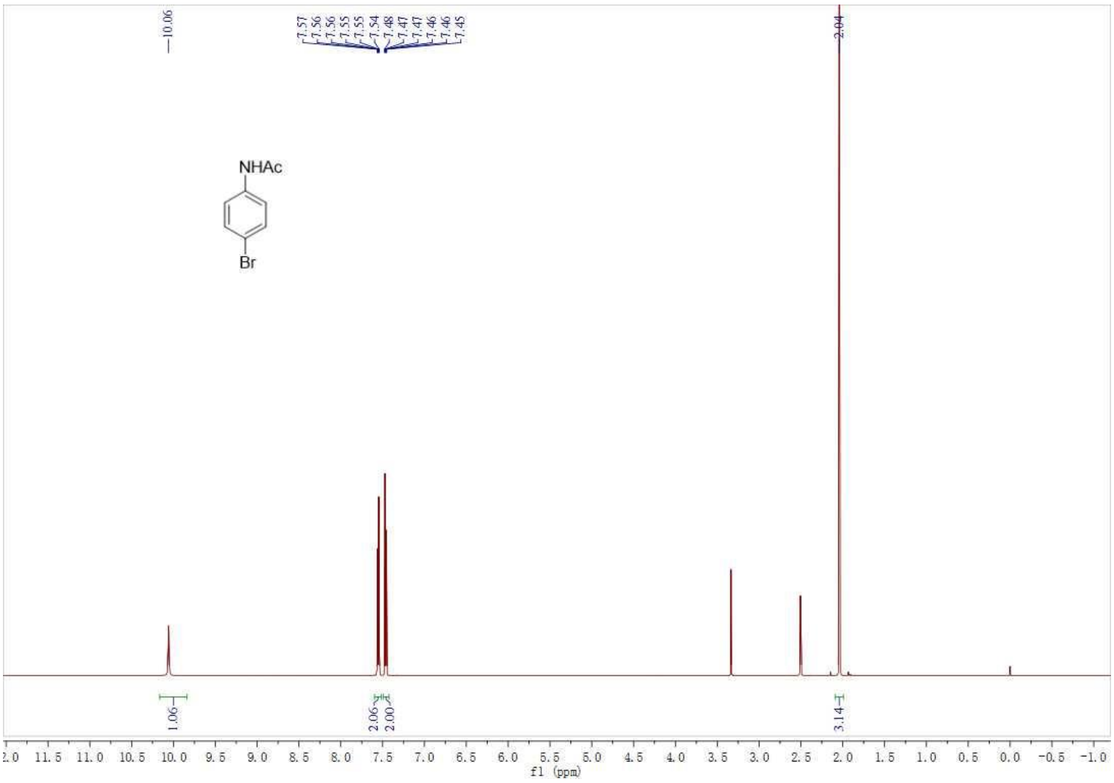

Figure S8. The ^{1}H-NMR of intermediate 10a

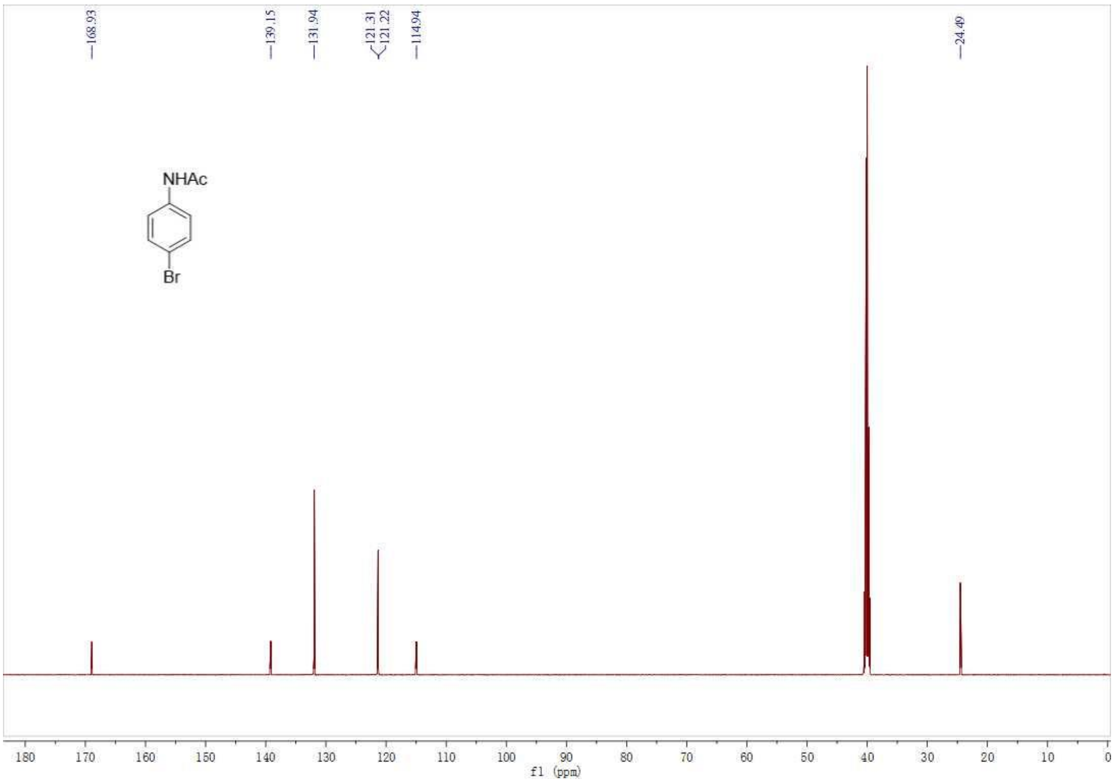

Figure S9. The ^{13}C-NMR of intermediate 10a

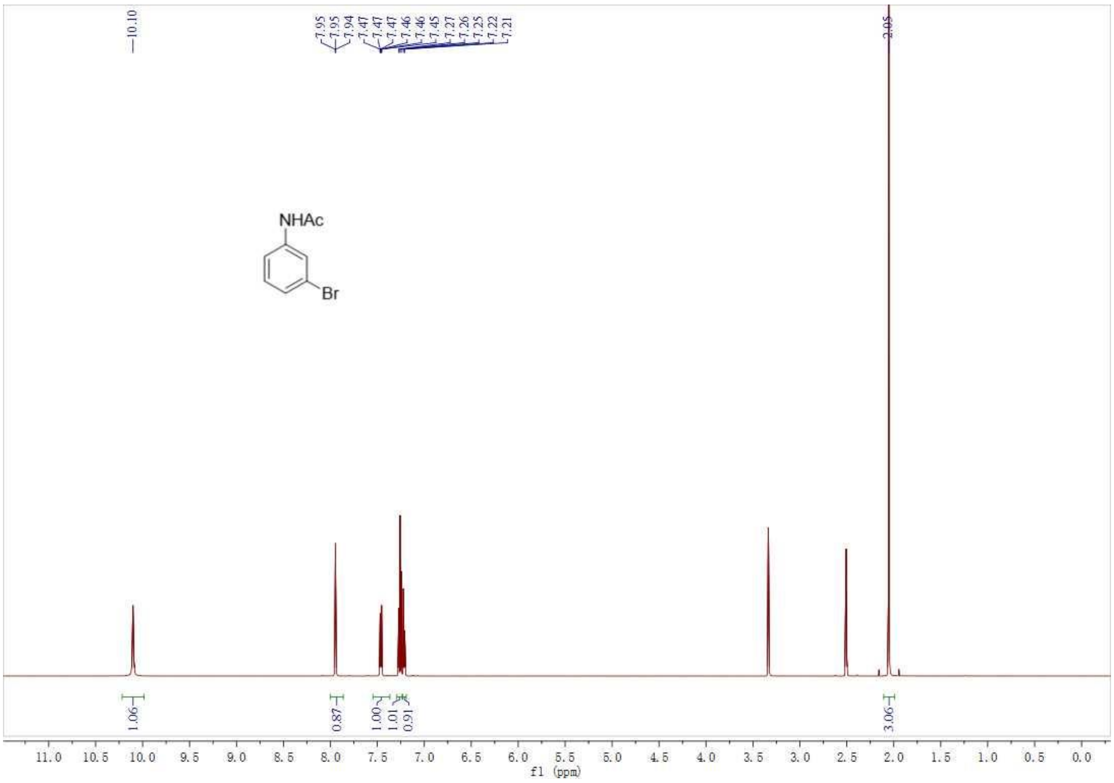

Figure S10. The ^{1}H-NMR of intermediate 10b

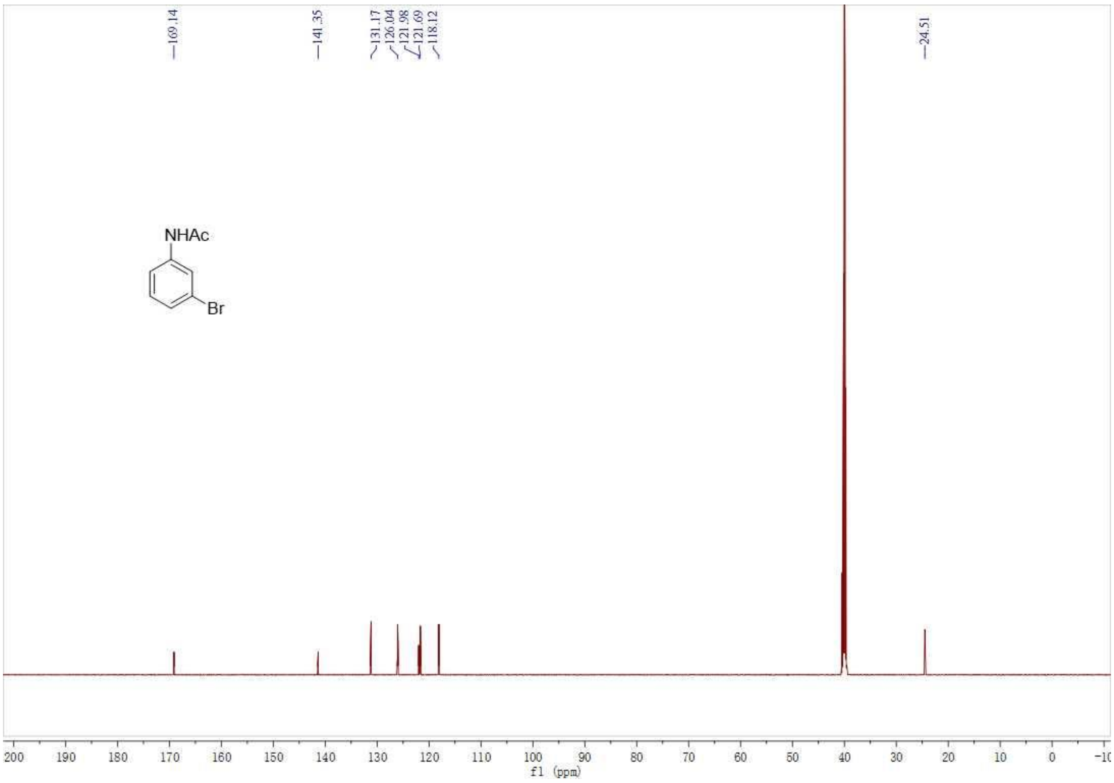

Figure S11. The ^{13}C-NMR of intermediate 10b

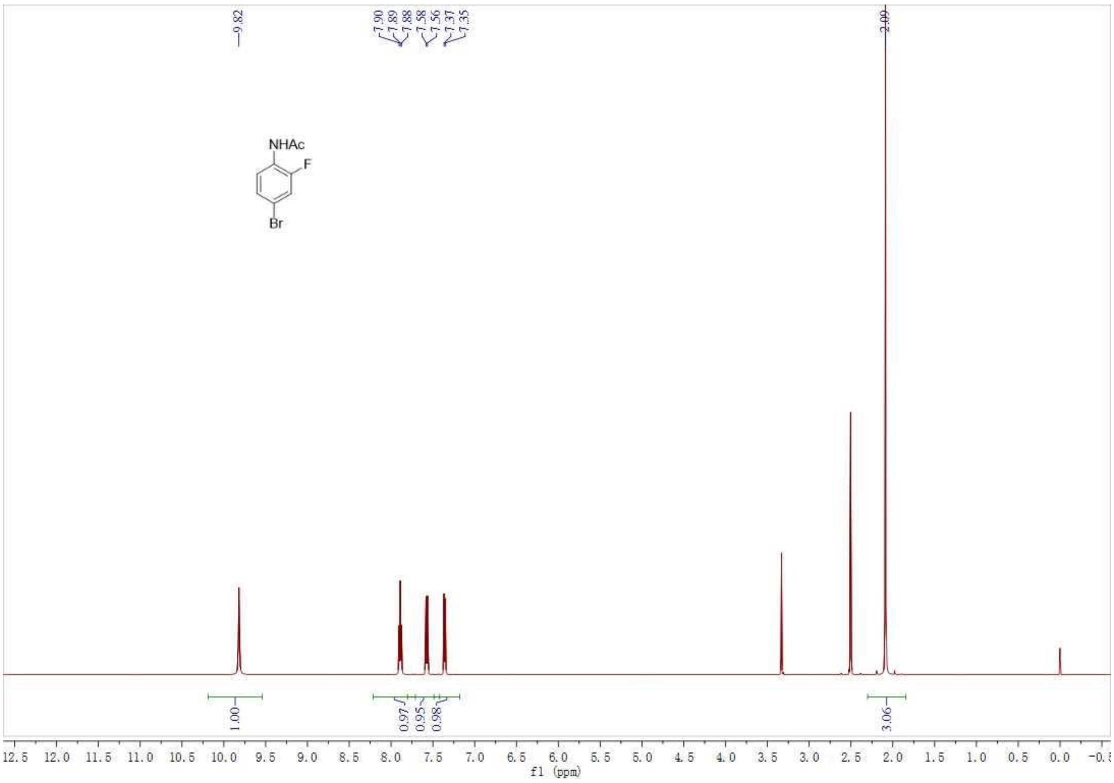

Figure S12. The ^{1}H-NMR of intermediate 10c

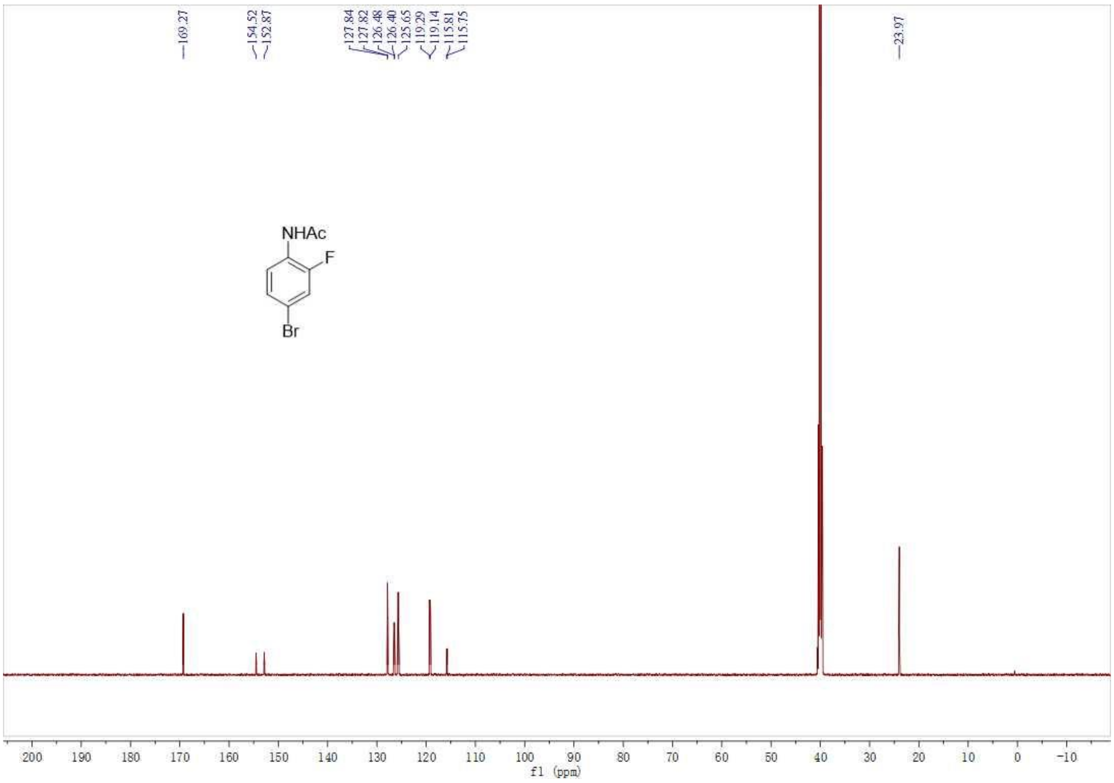

Figure S13. The ^{13}C-NMR of intermediate 10c

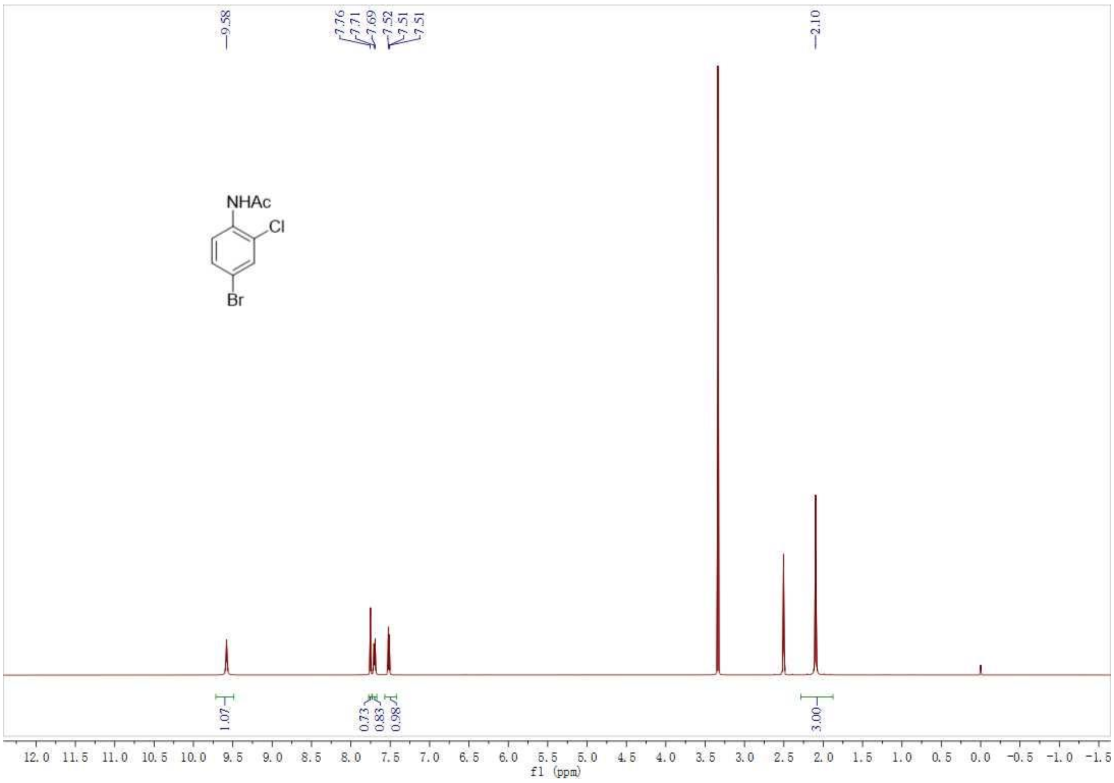

Figure S14. The ^{1}H-NMR of intermediate 10d

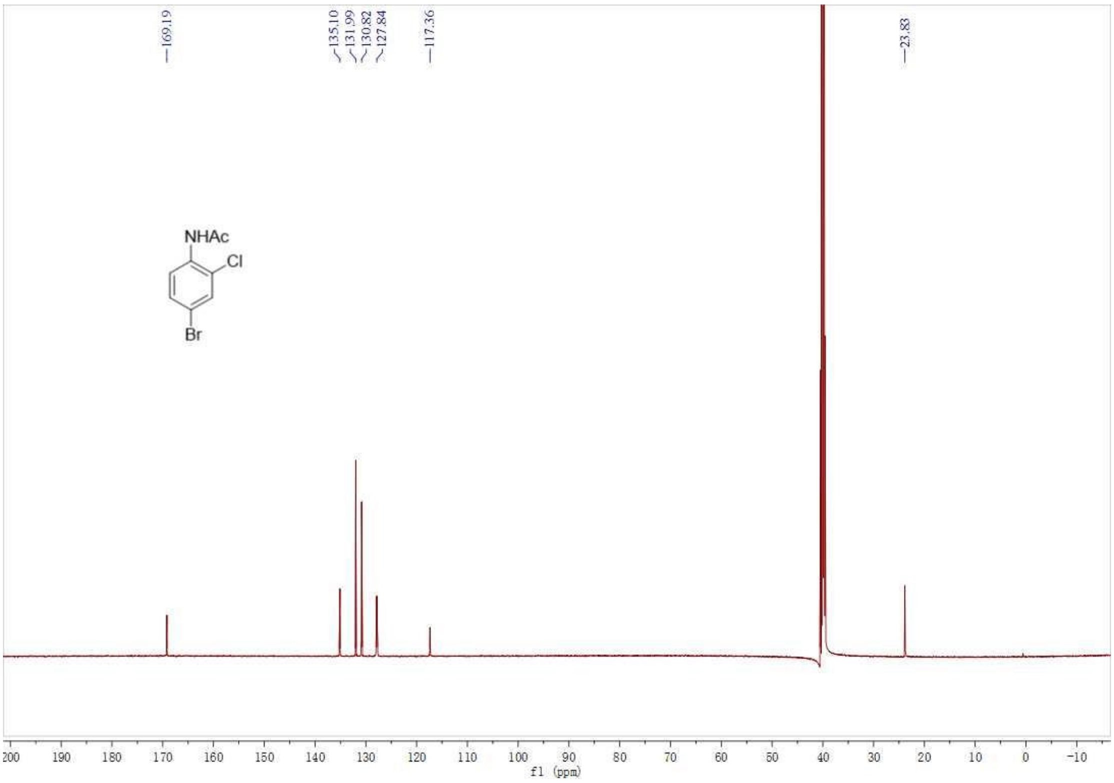

Figure S15. The ^{13}C-NMR of intermediate 10d

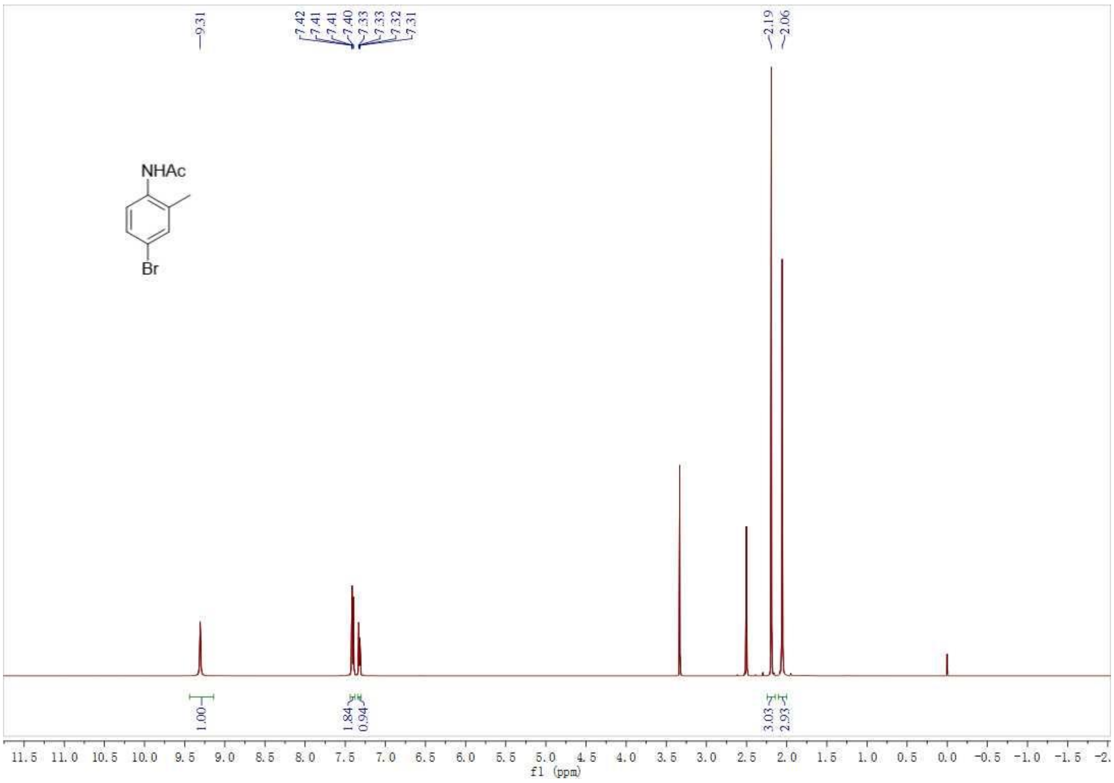

Figure S16. The ^{1}H-NMR of intermediate 10e

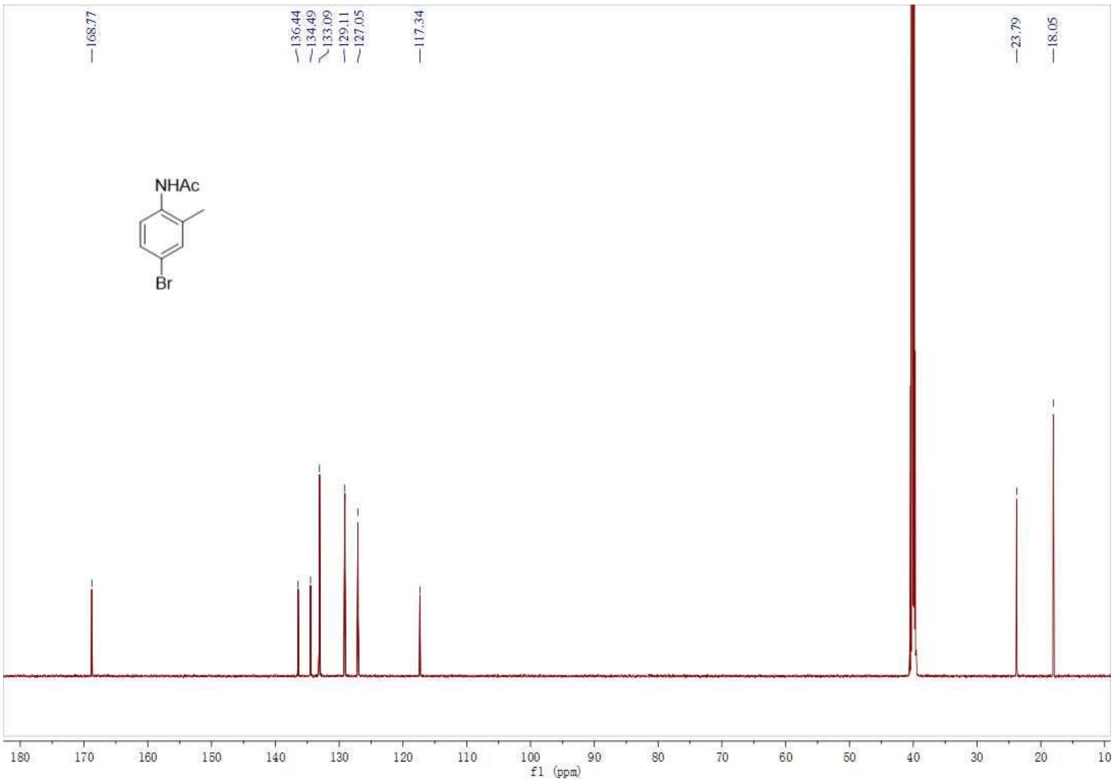

Figure S17. The ^{13}C-NMR of intermediate 10e

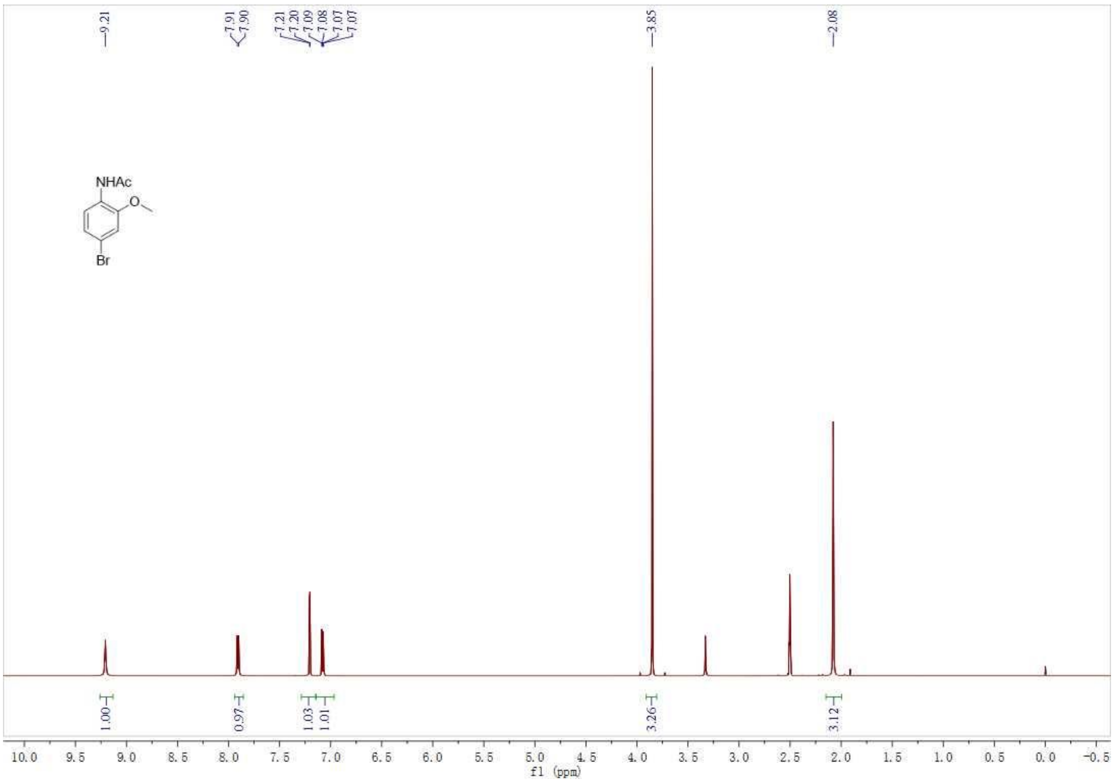

Figure S18. The ^{1}H-NMR of intermediate 10f

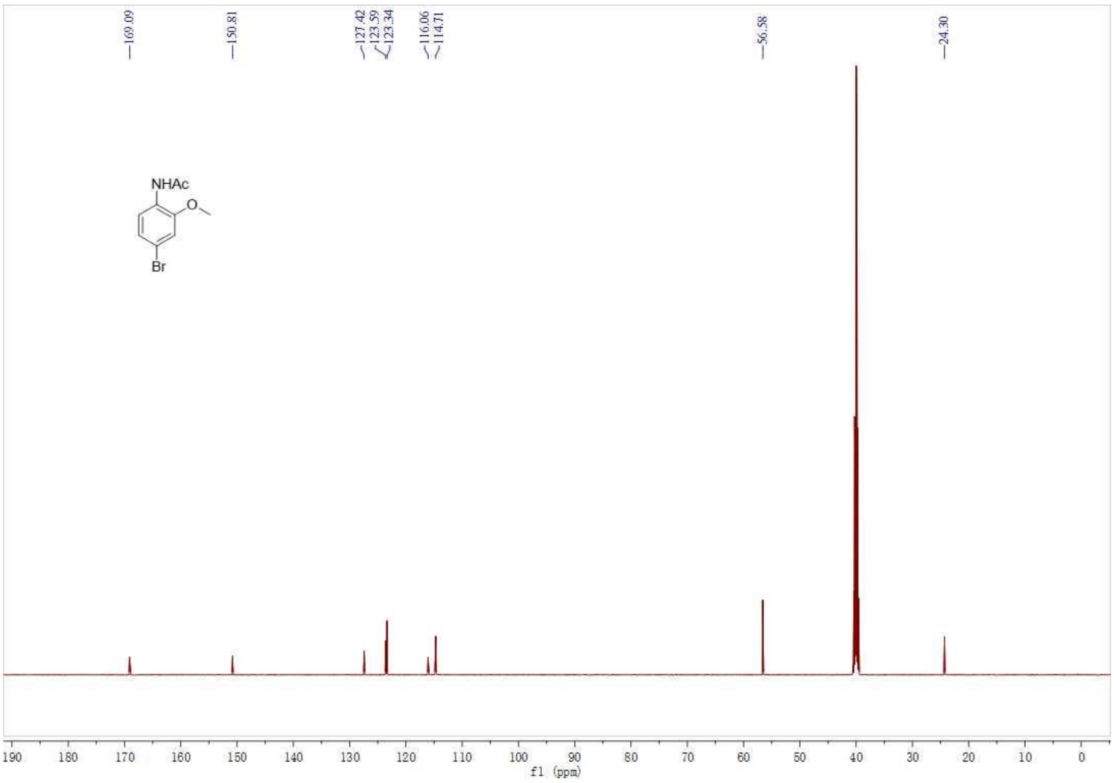

Figure S19. The ^{13}C-NMR of intermediate 10f

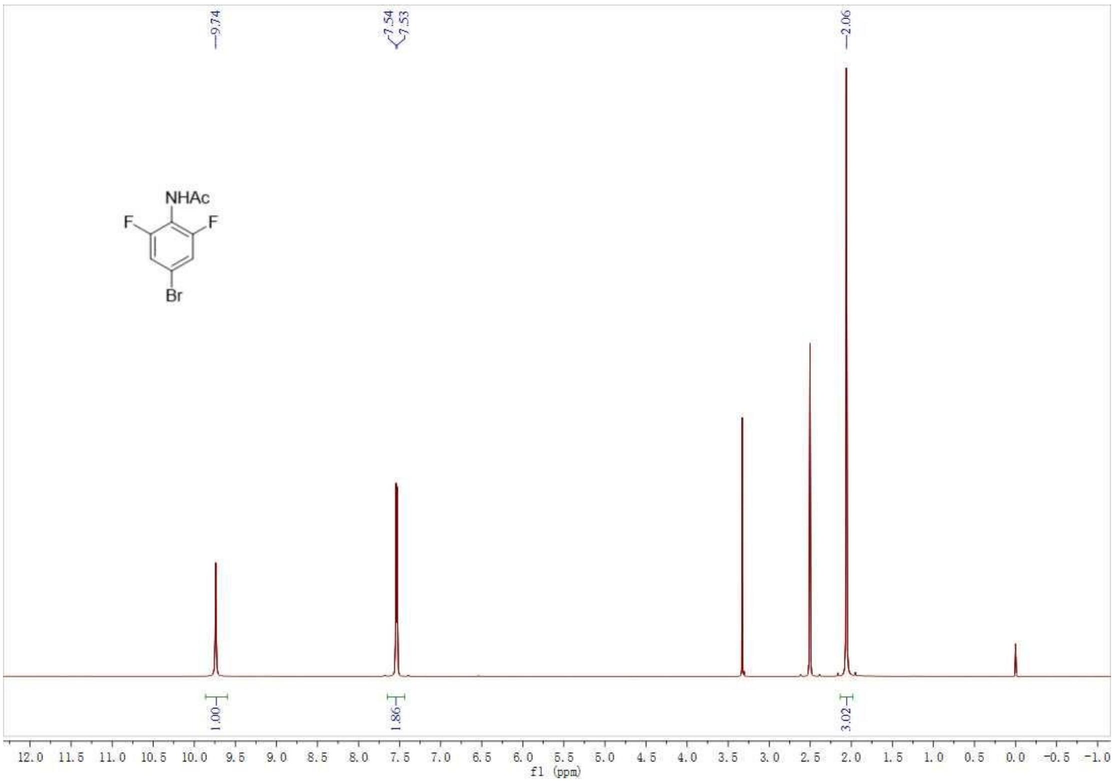

Figure S20. The ^{1}H-NMR of intermediate 10g

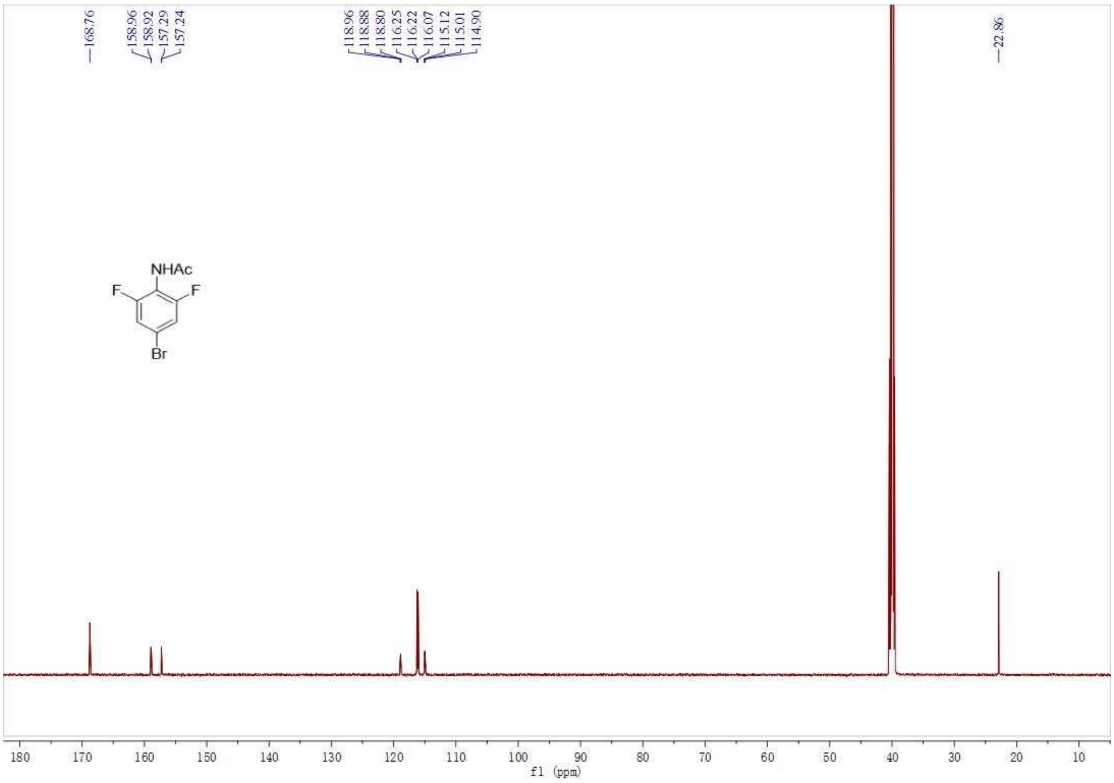

Figure S21. The ^{13}C-NMR of intermediate 10g

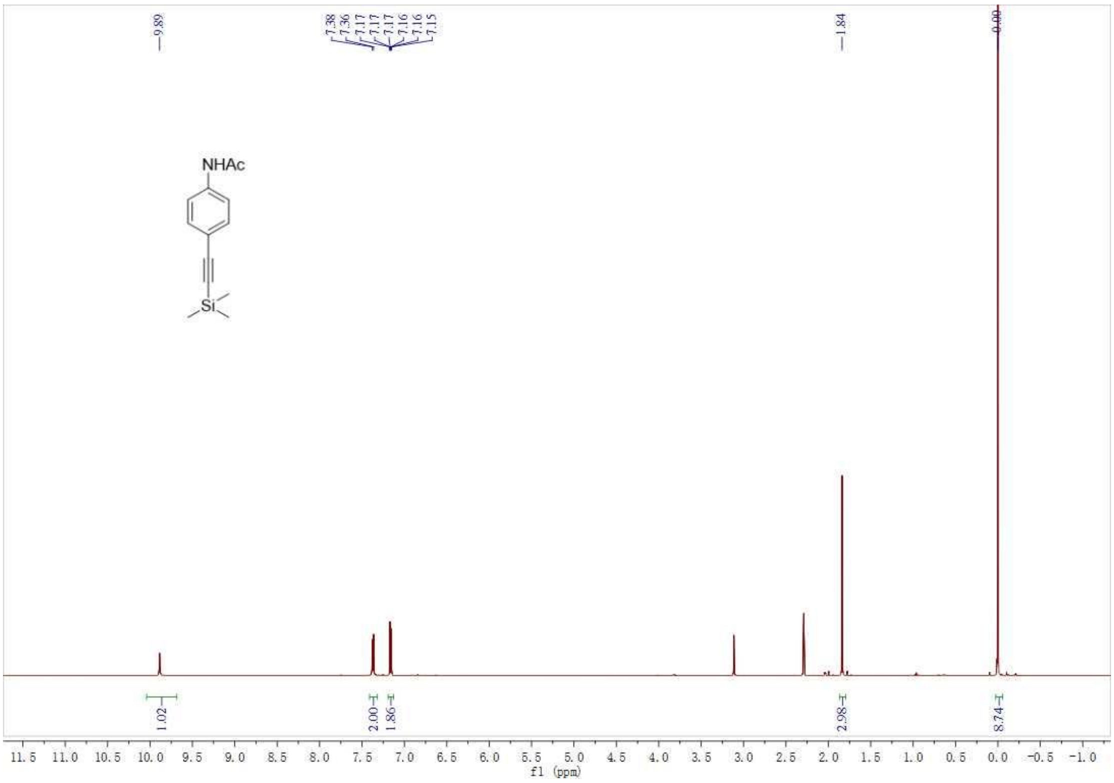

Figure S22. The ^{1}H-NMR of intermediate 11a

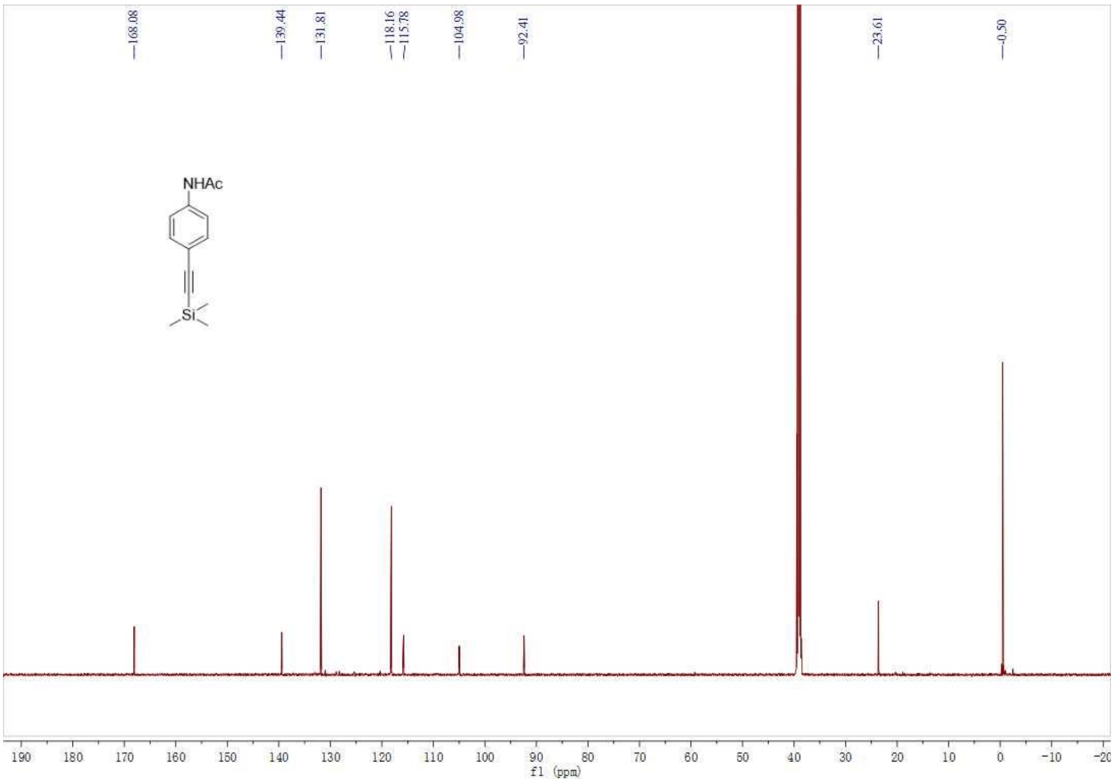

Figure S23. The ^{13}C-NMR of intermediate 11a

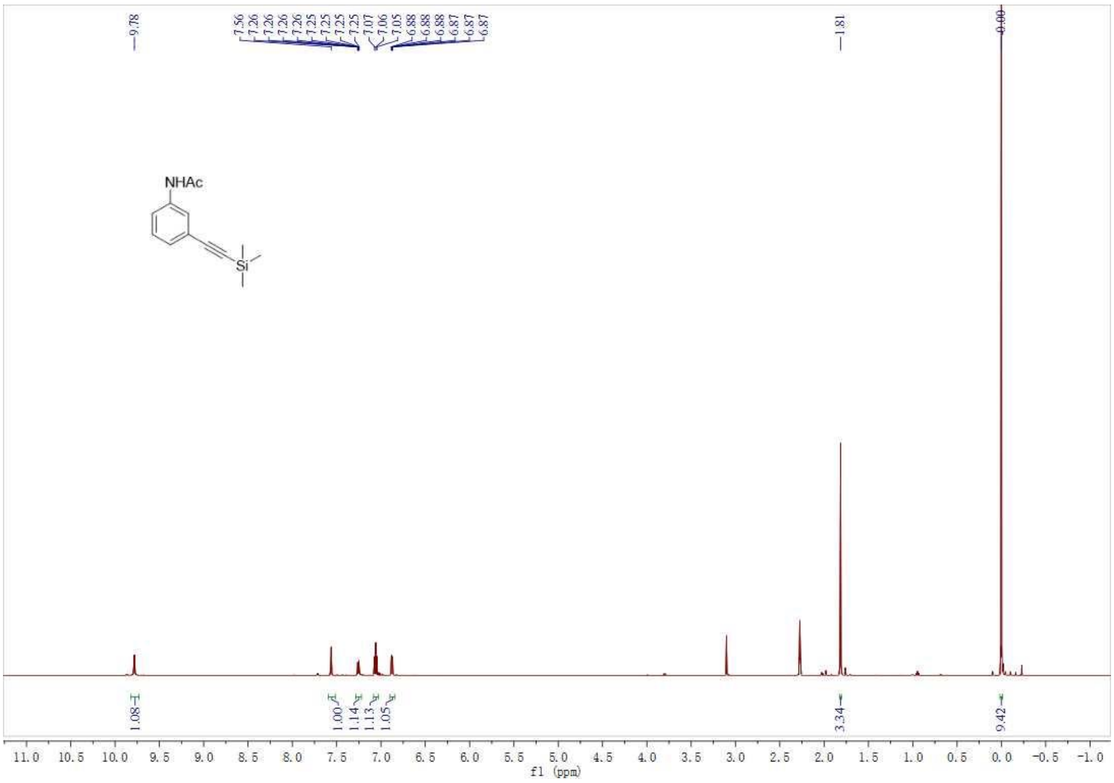

Figure S24. The ^{1}H-NMR of intermediate 11b

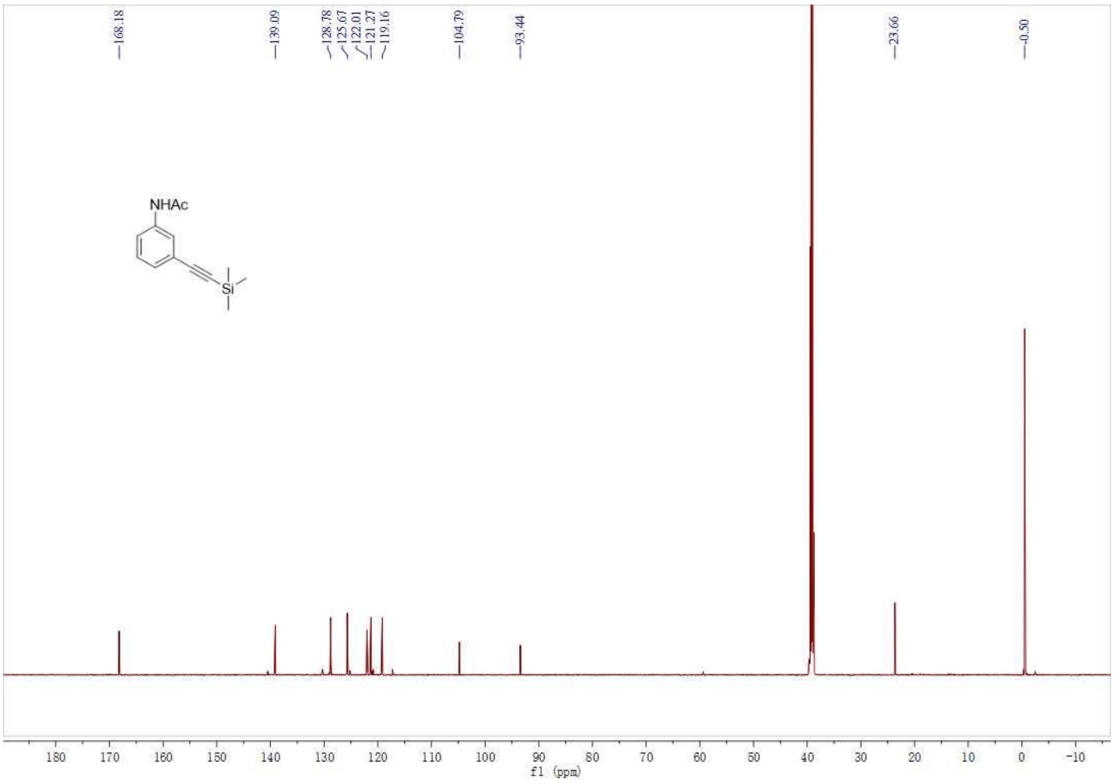

Figure S25. The ^{13}C-NMR of intermediate 11b

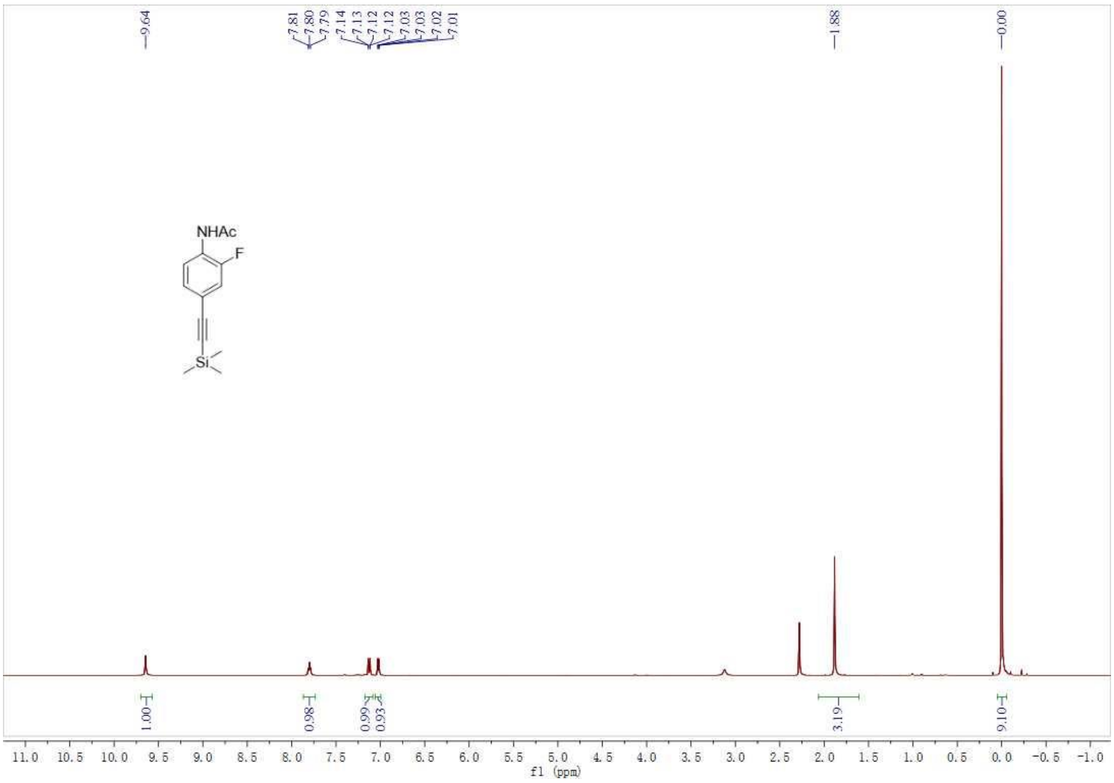

Figure S26. The ^{1}H-NMR of intermediate 11c

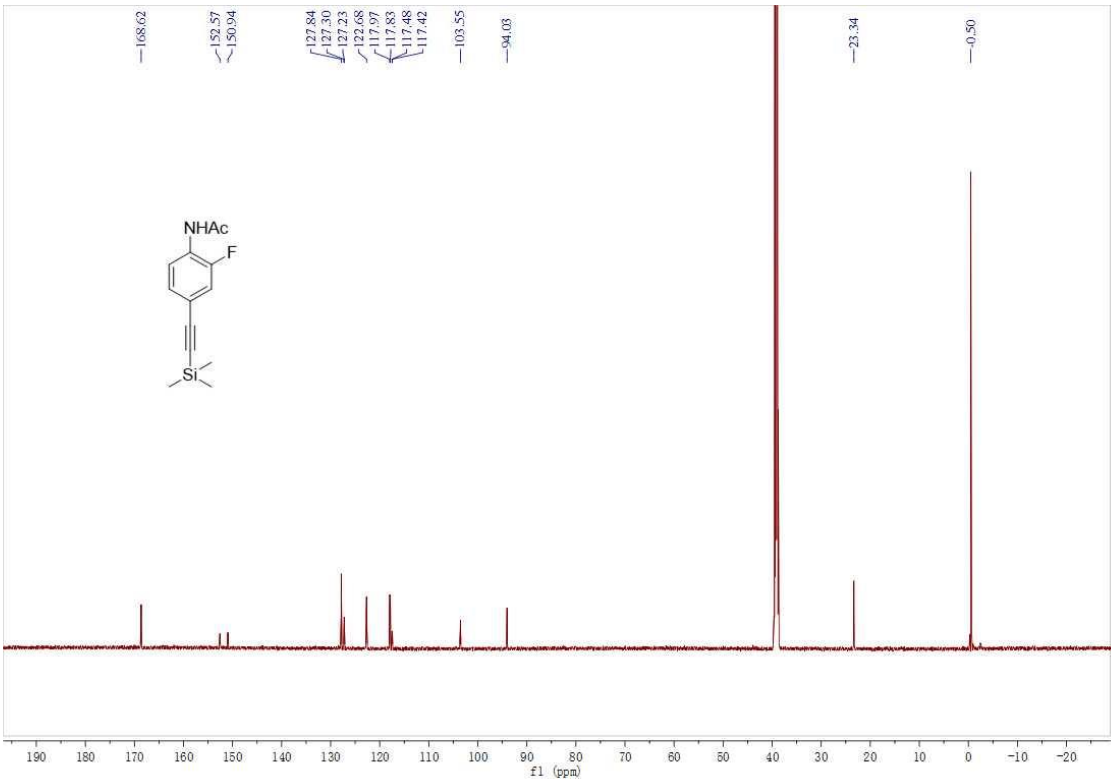

Figure S27. The ^{13}C-NMR of intermediate 11c

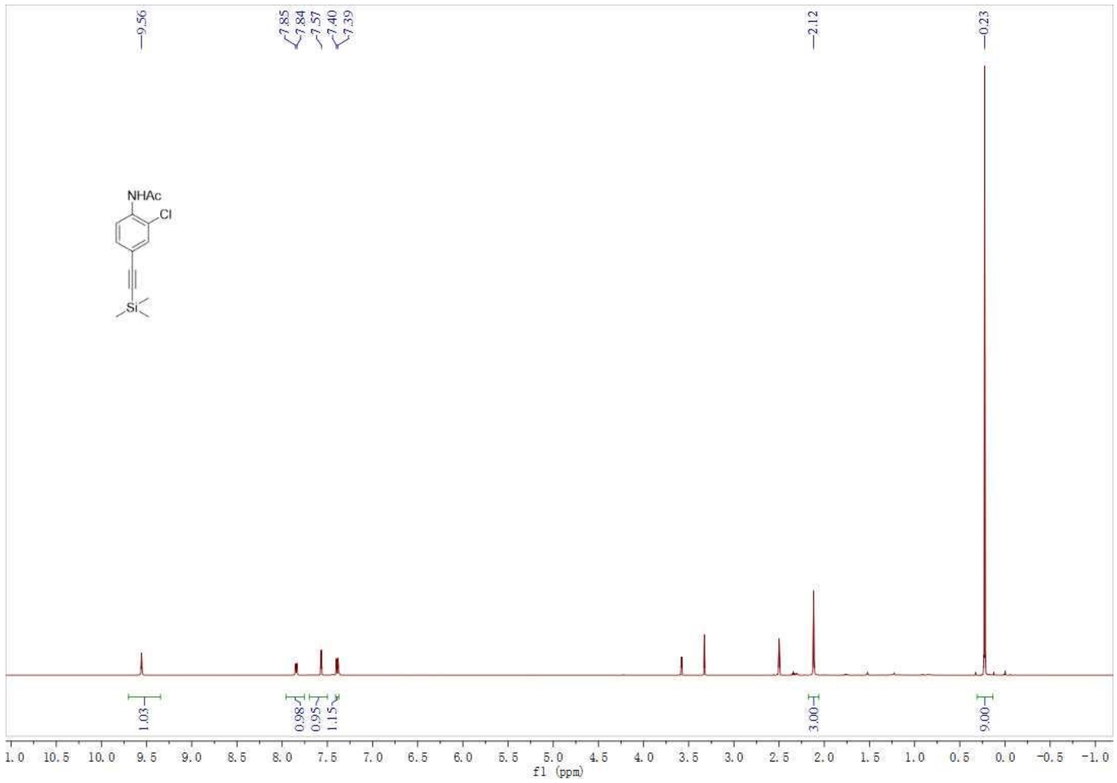

Figure S28. The ^{1}H-NMR of intermediate 11d

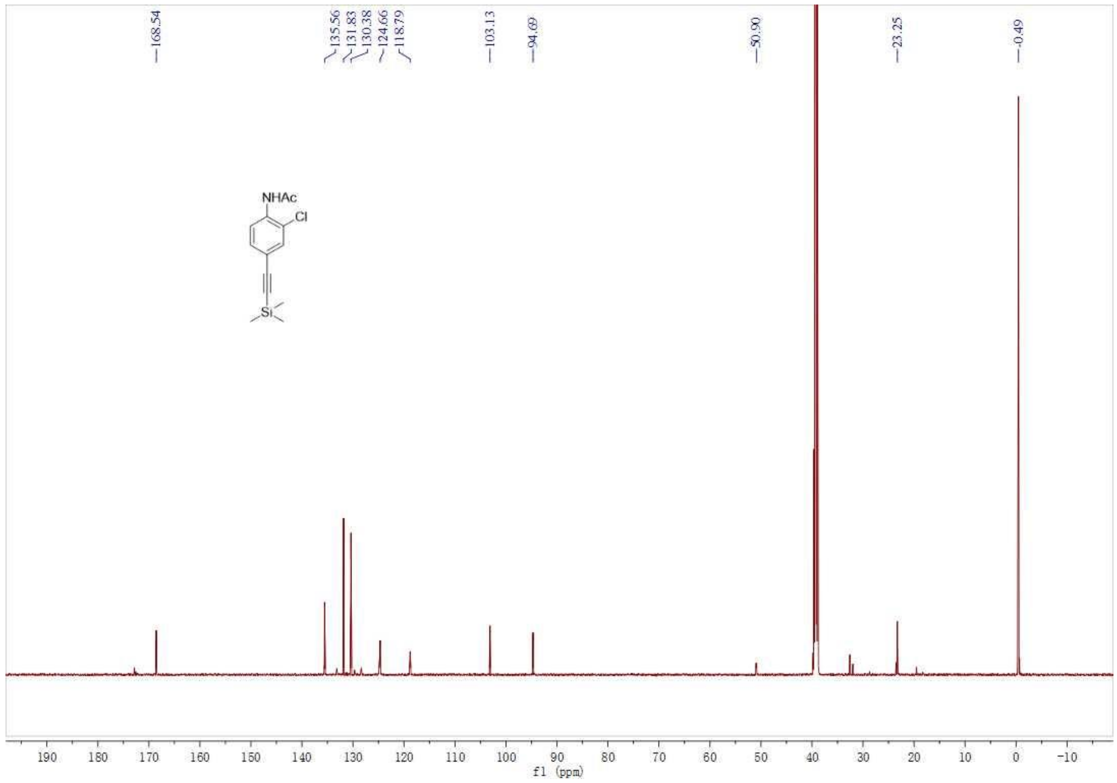

Figure S29. The ^{13}C-NMR of intermediate 11d

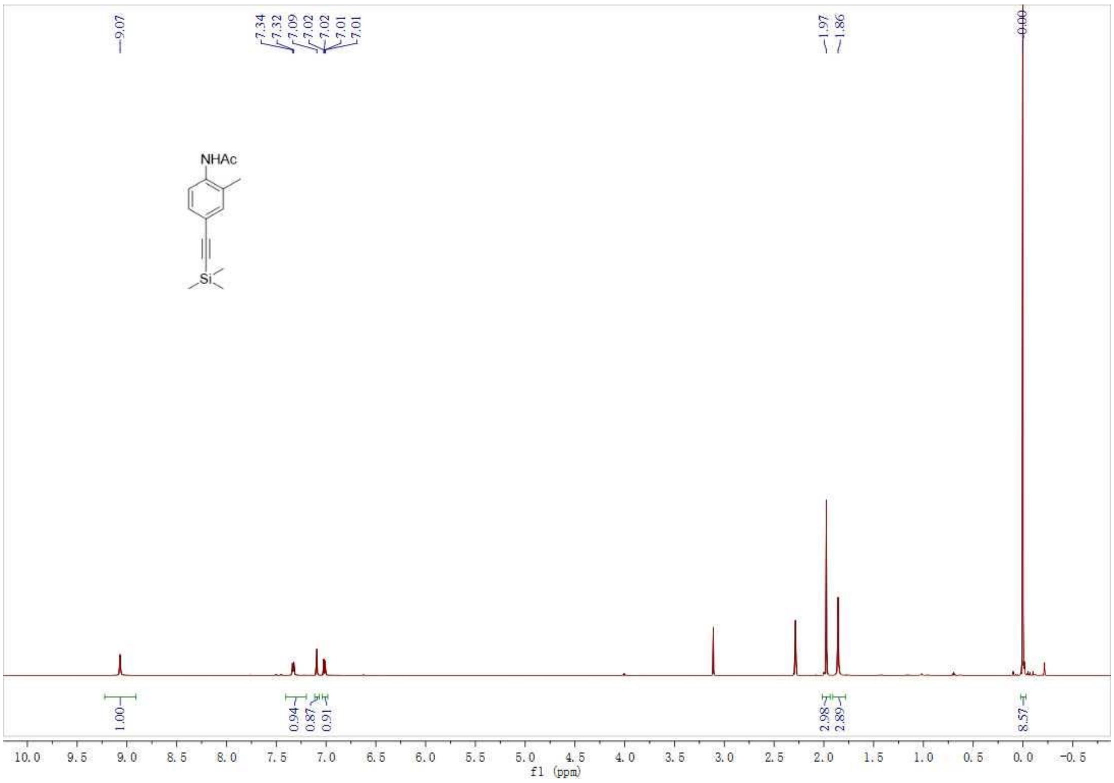

Figure S30. The ^{1}H-NMR of intermediate 11e

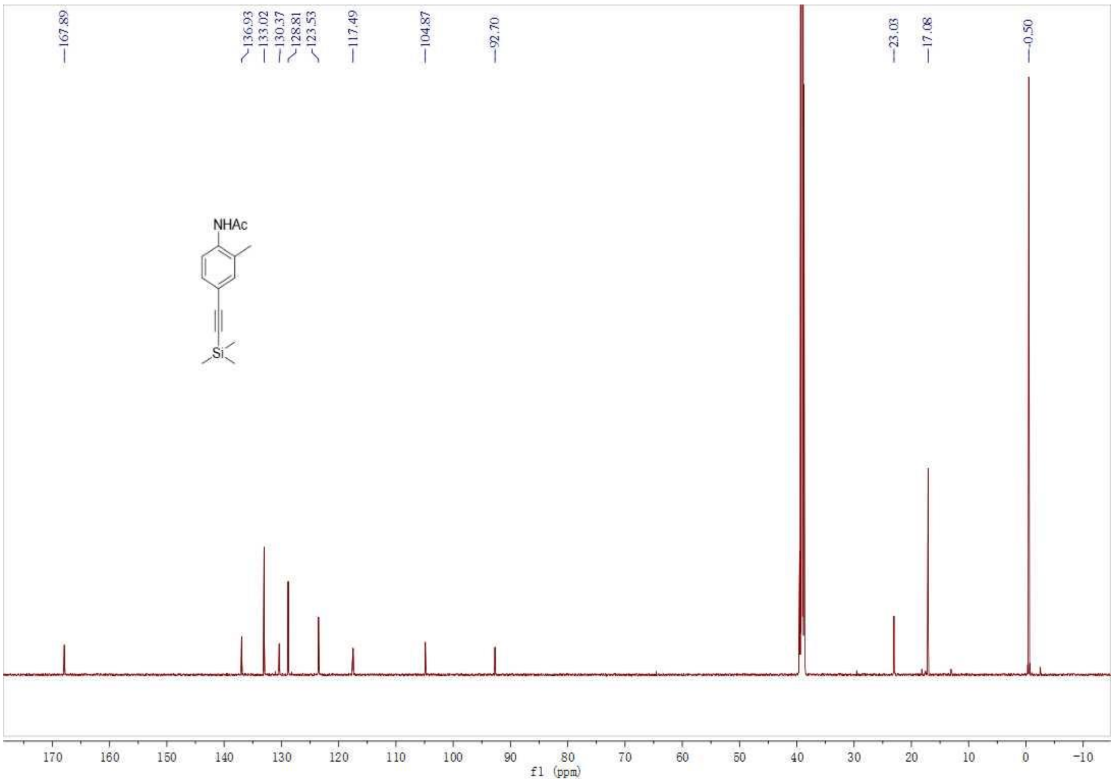

Figure S31. The ^{13}C-NMR of intermediate 11e

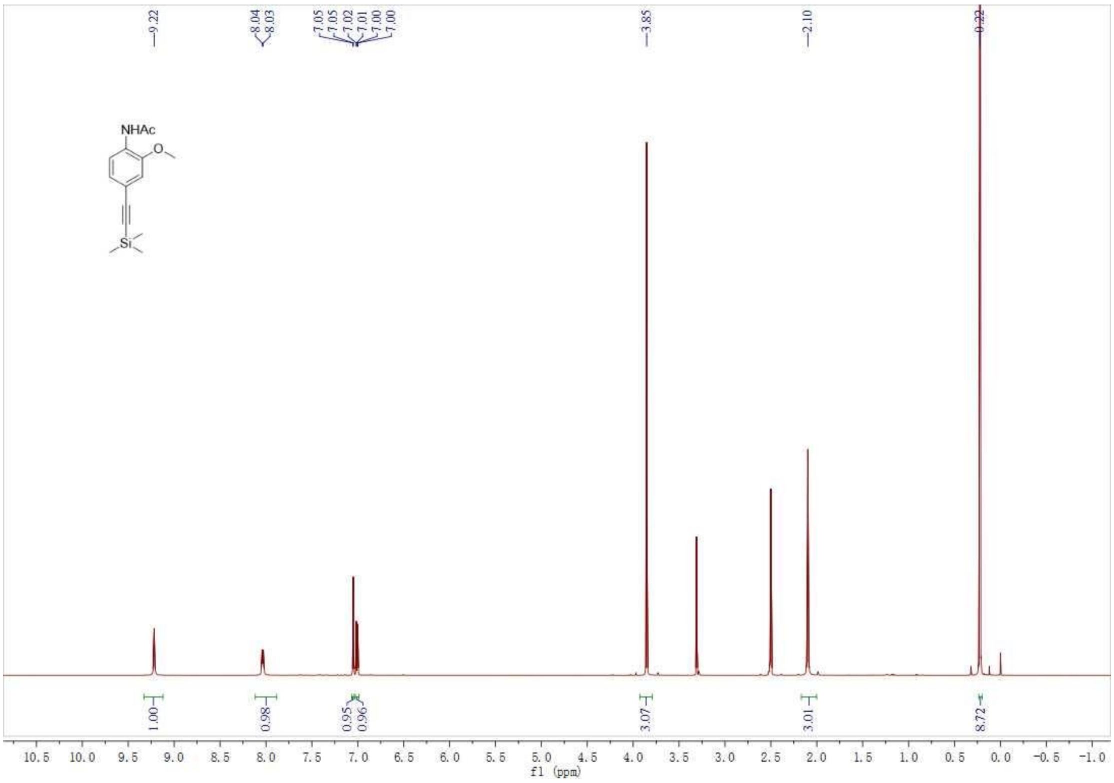

Figure S32. The ^{1}H-NMR of intermediate 11f

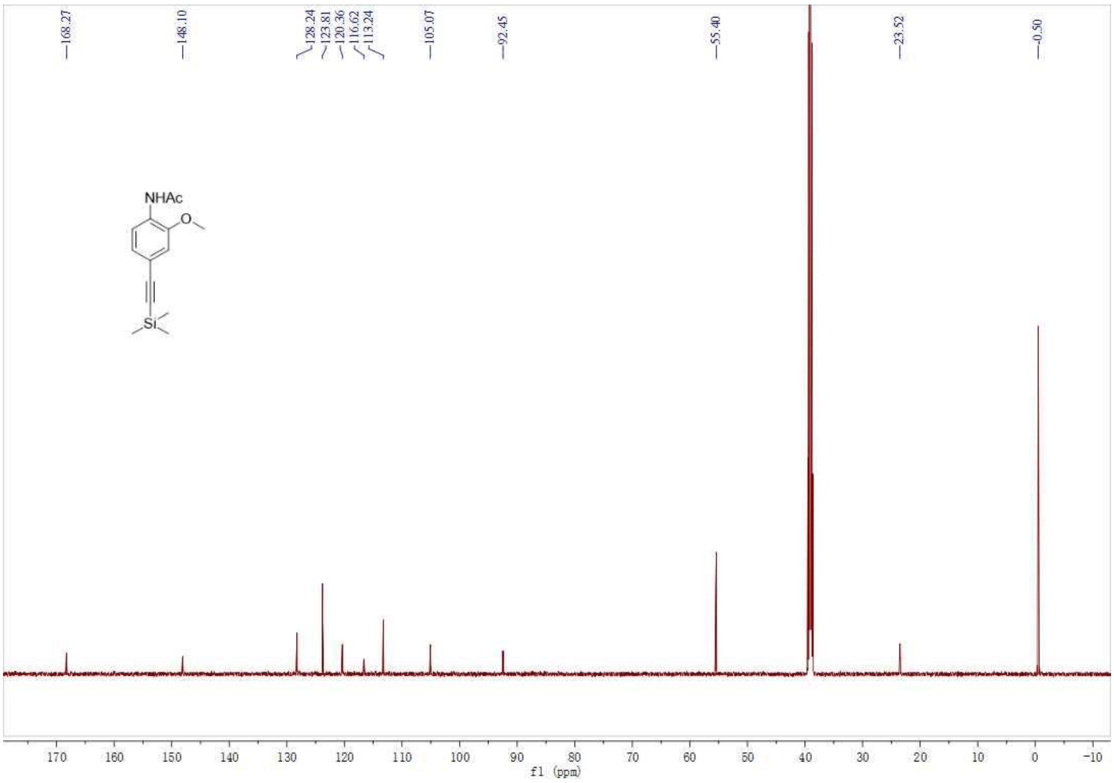

Figure S33. The ^{13}C-NMR of intermediate 11f

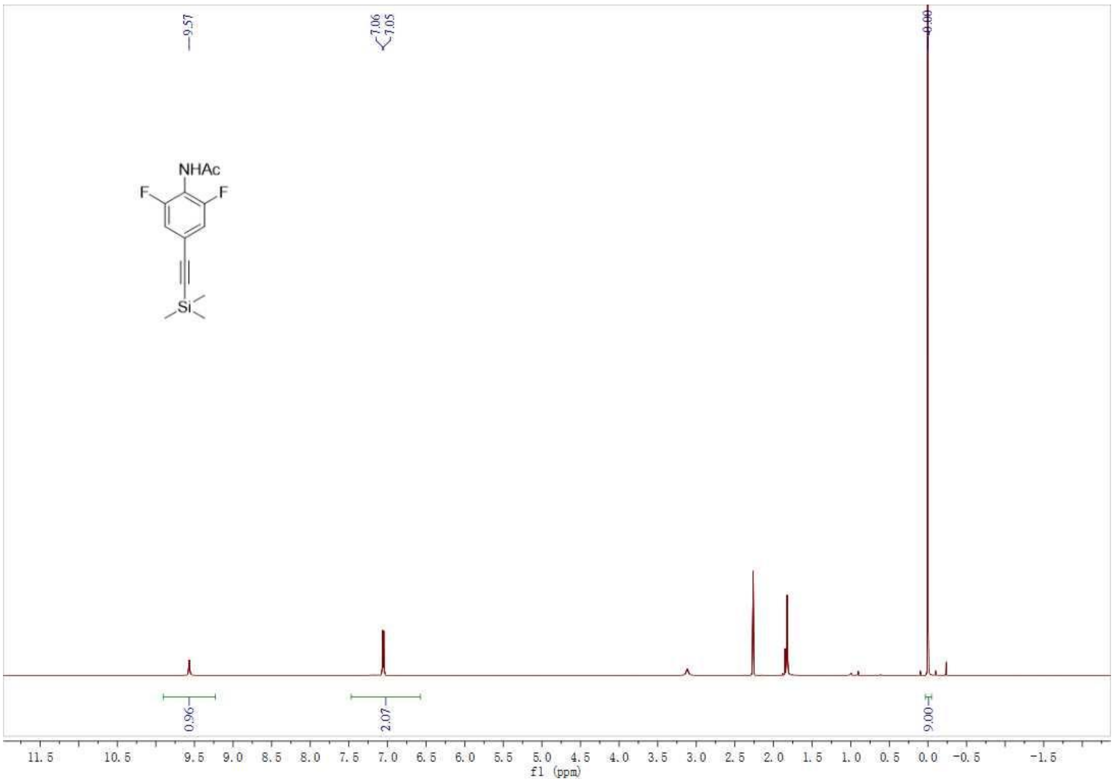

Figure S34. The ^{1}H-NMR of intermediate 11g

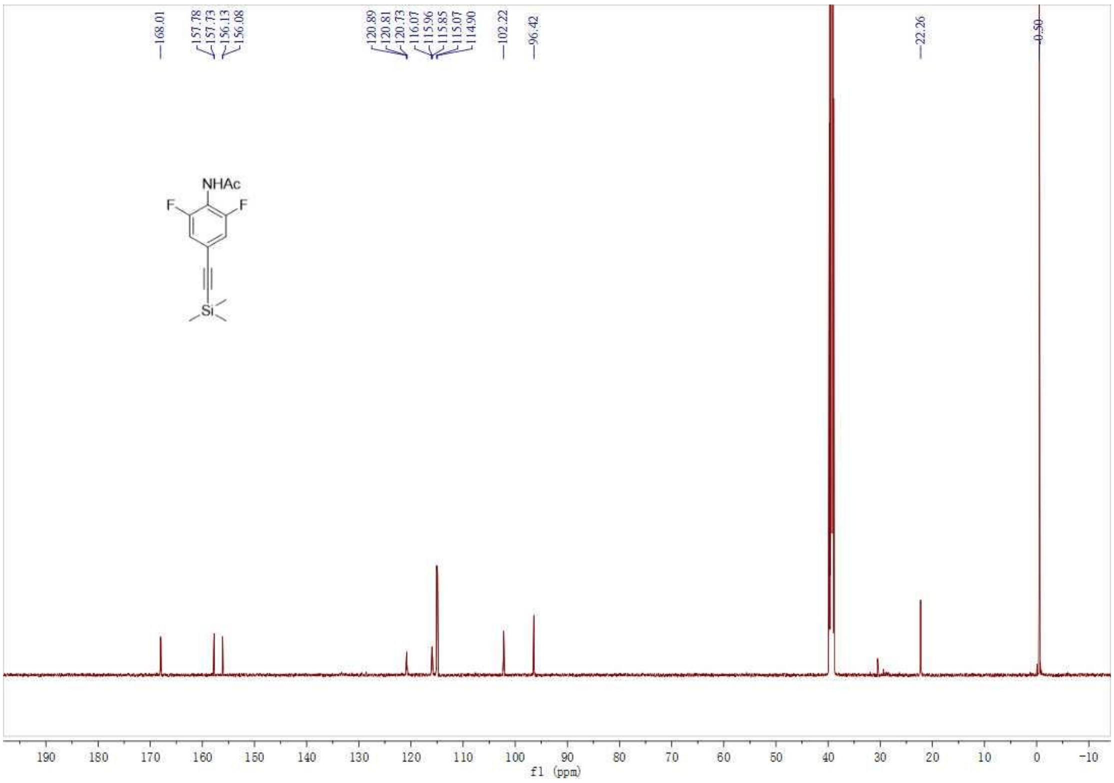

Figure S35. The ^{13}C-NMR of intermediate 11g

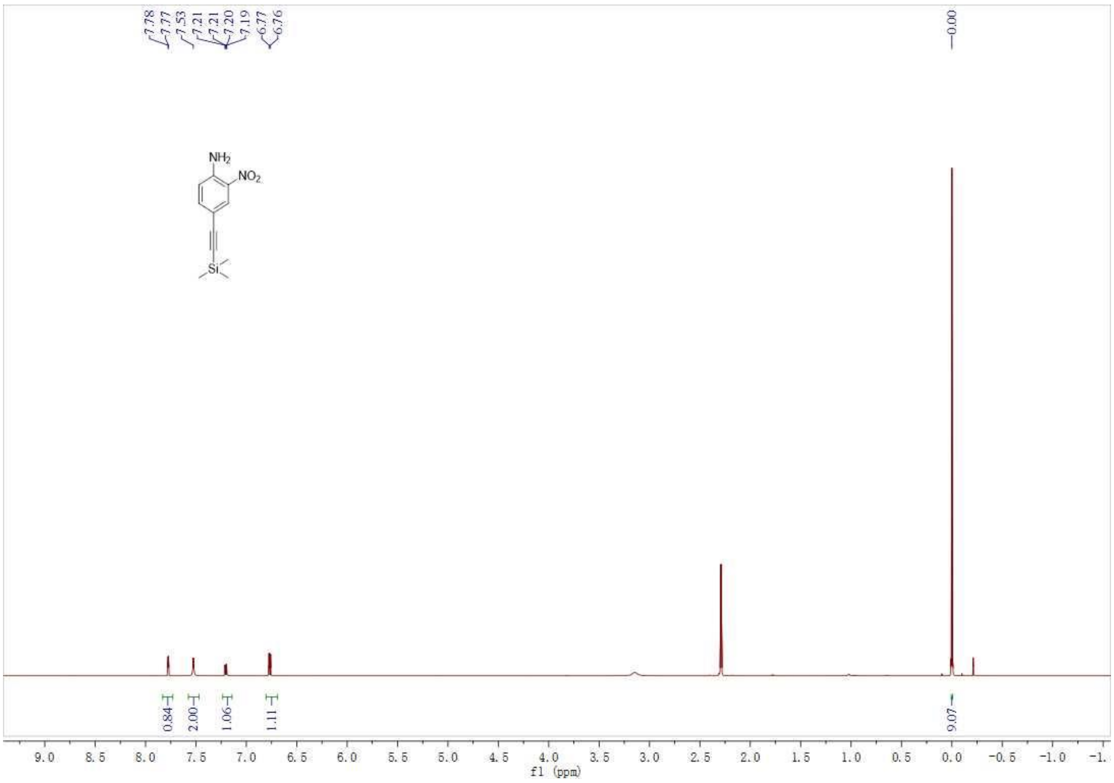

Figure S36. The ^{1}H-NMR of intermediate 11h

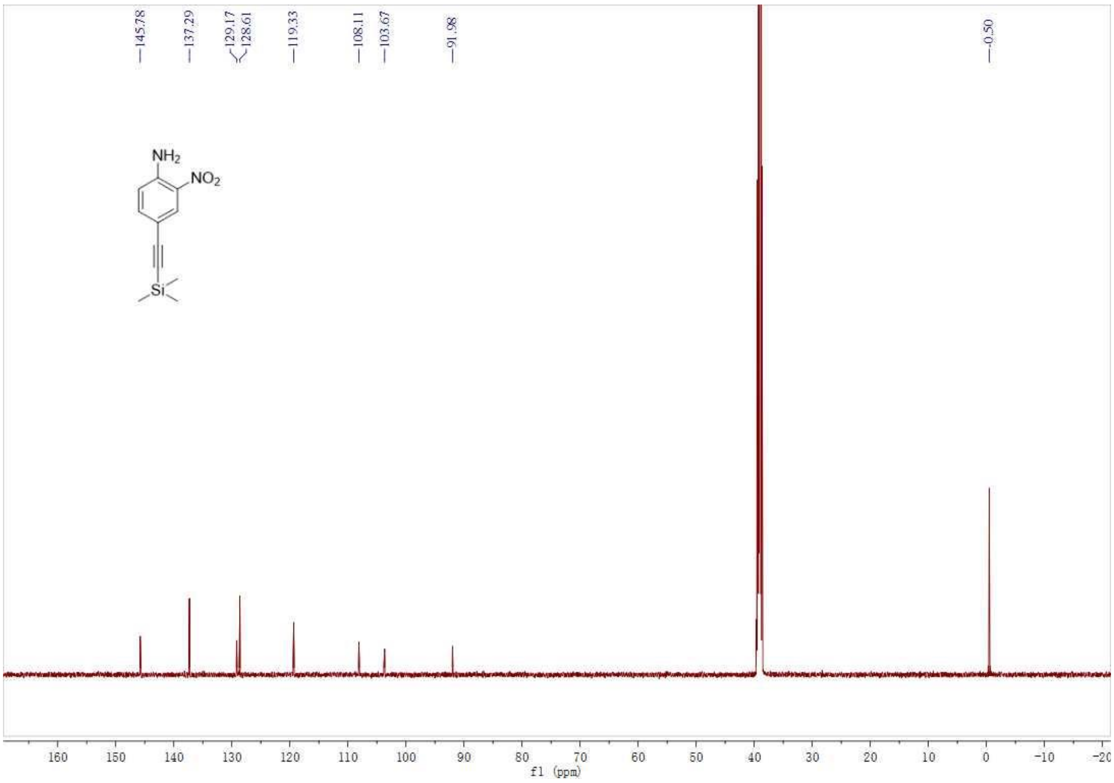

Figure S37. The ^{13}C-NMR of intermediate 11h

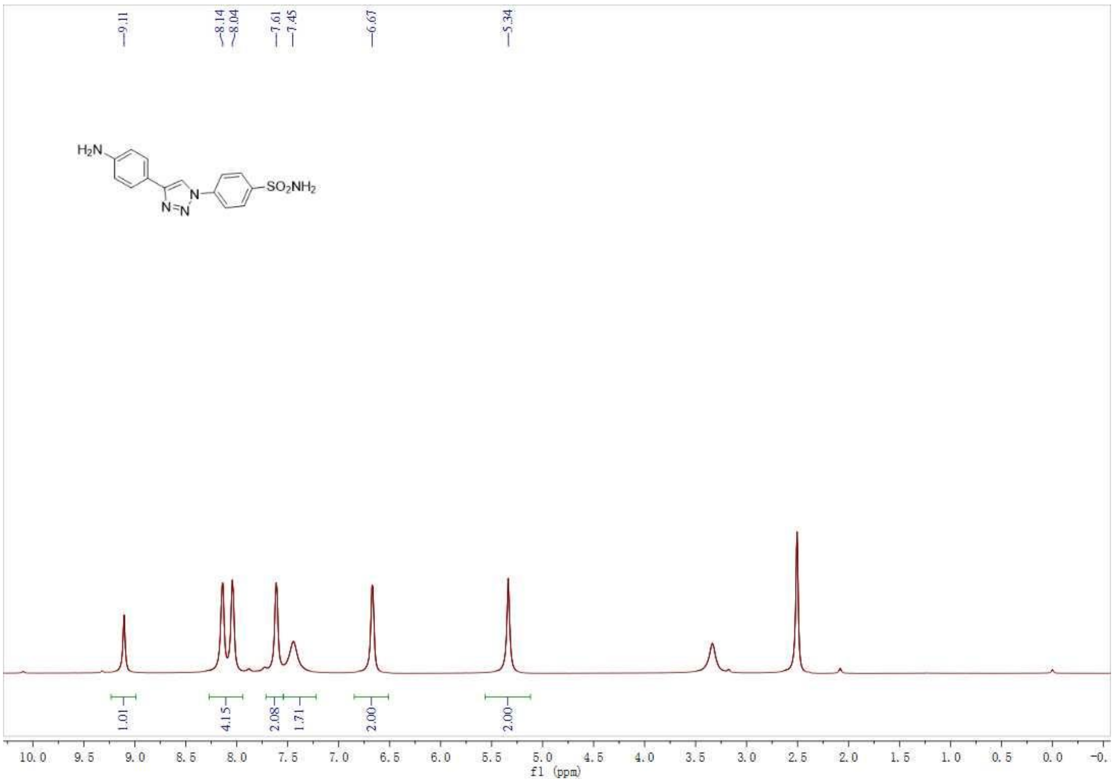

Figure S38. The ^{1}H-NMR of intermediate 14a

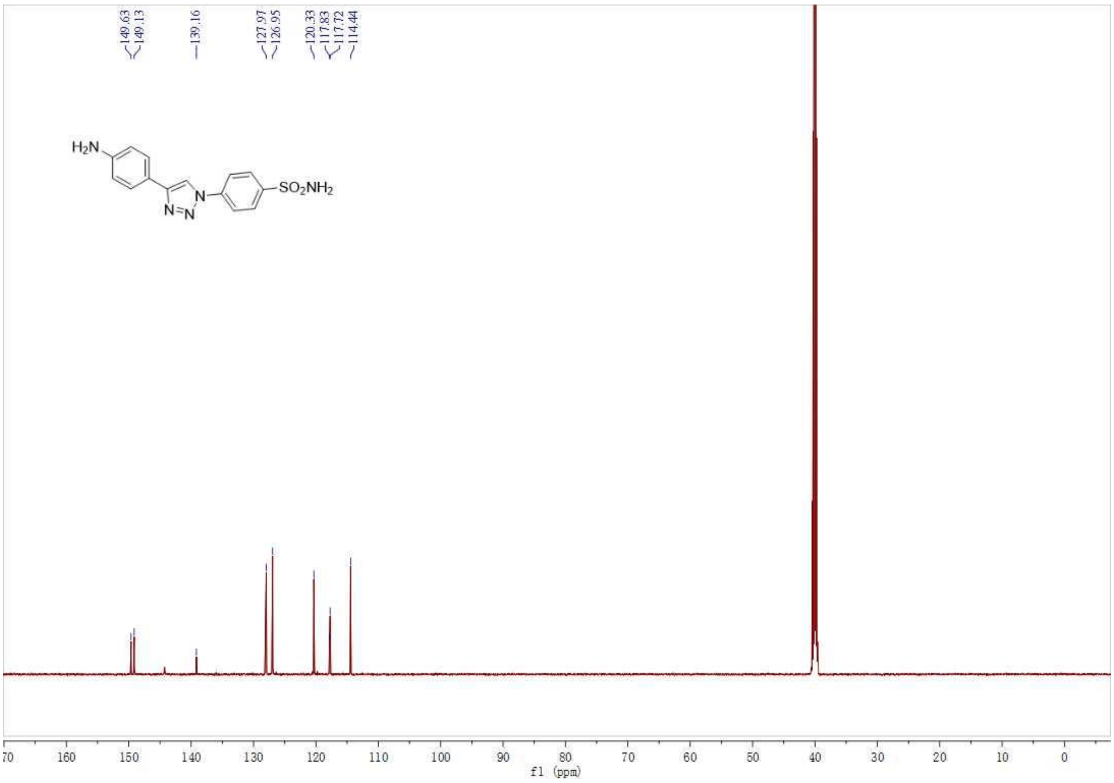

Figure S39. The ^{13}C-NMR of intermediate 14a

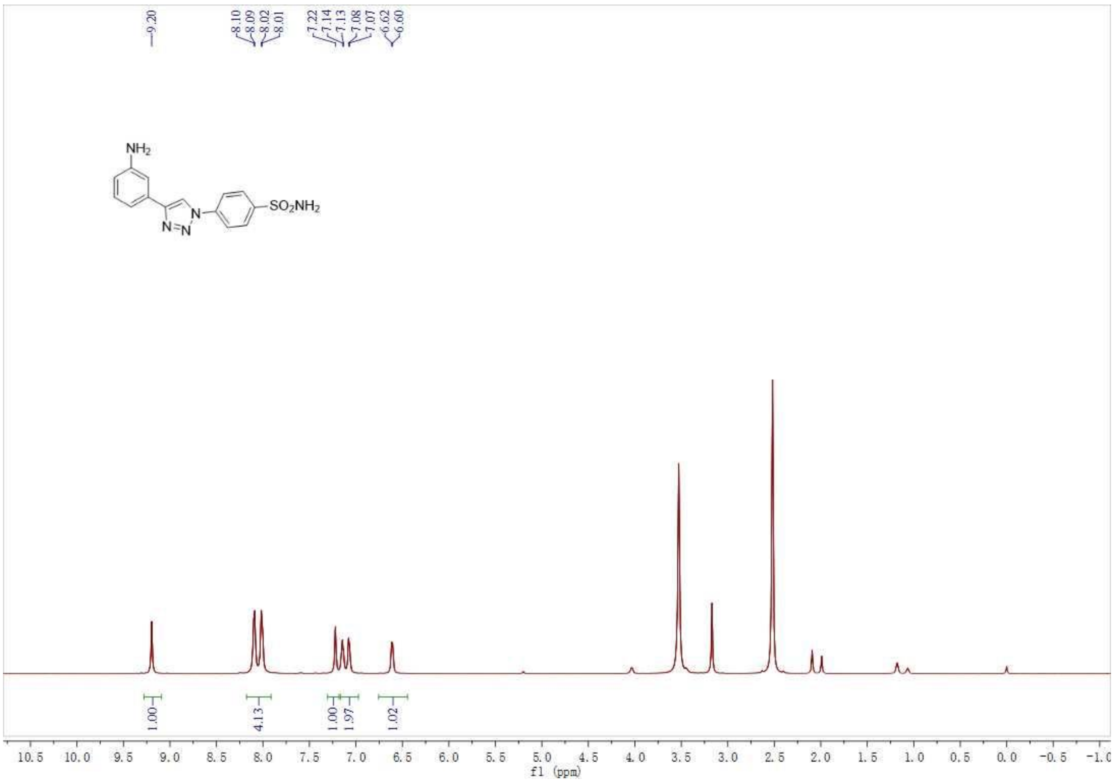

Figure S40. The ^{1}H-NMR of intermediate 14b

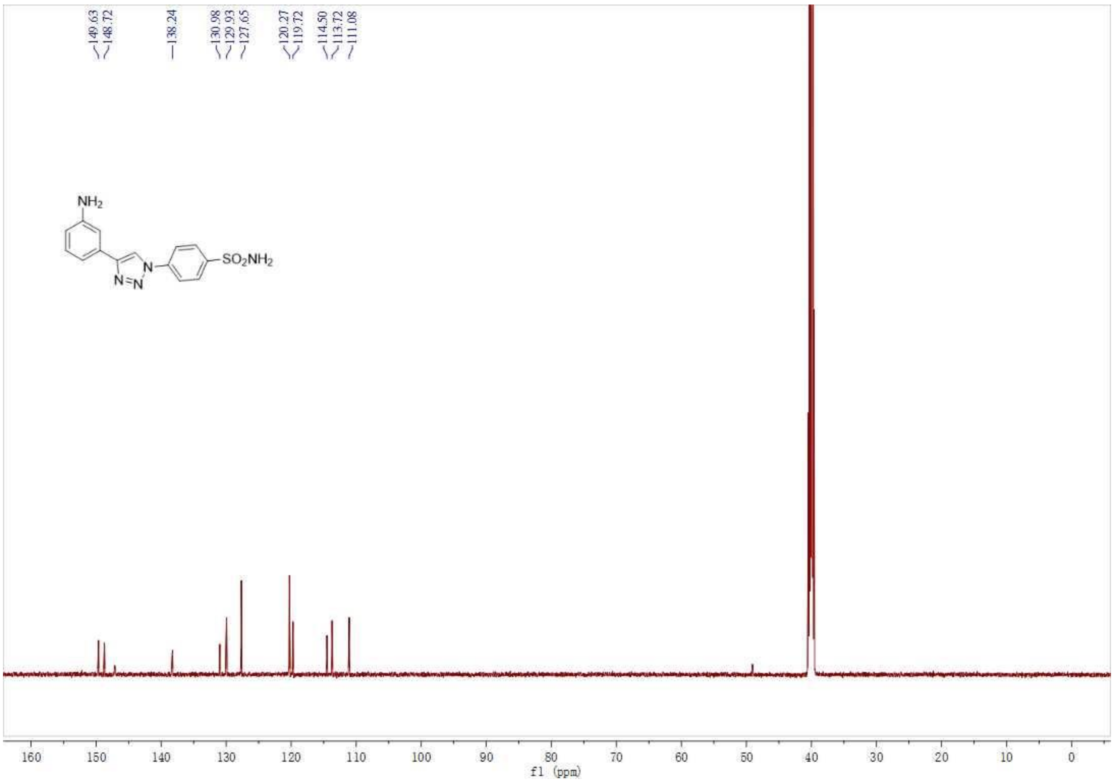

Figure S41. The ^{13}C-NMR of intermediate 14b

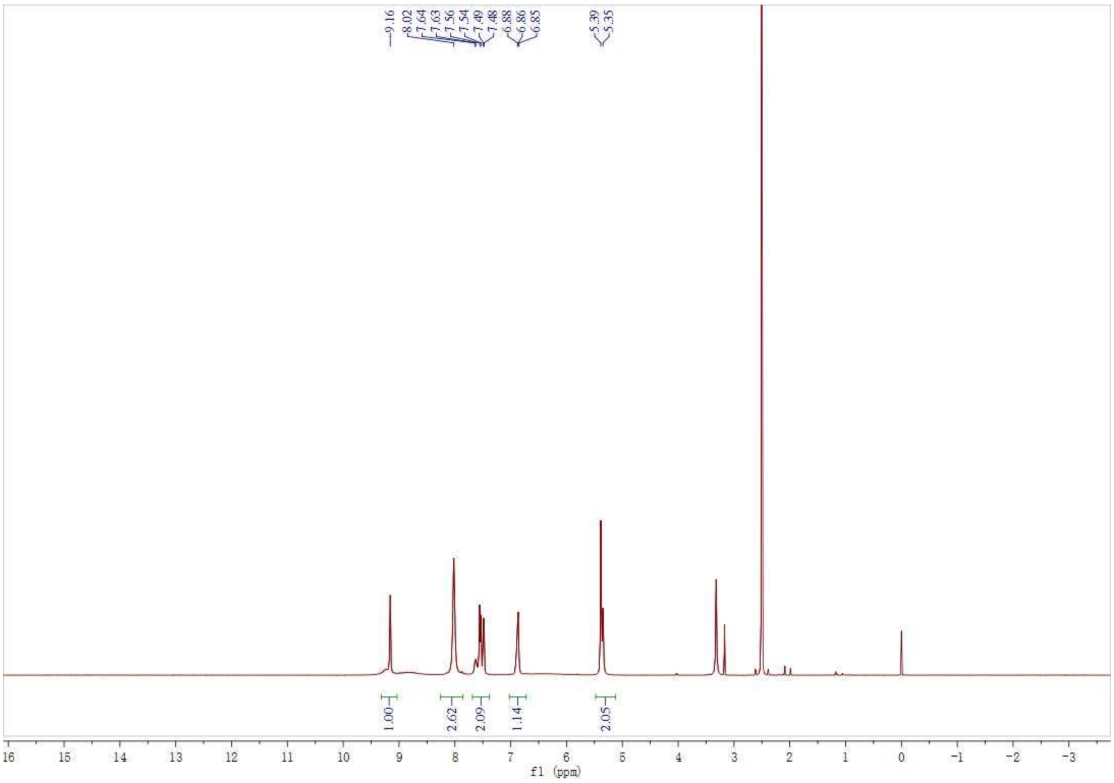

Figure S42. The ^{1}H-NMR of intermediate 14c

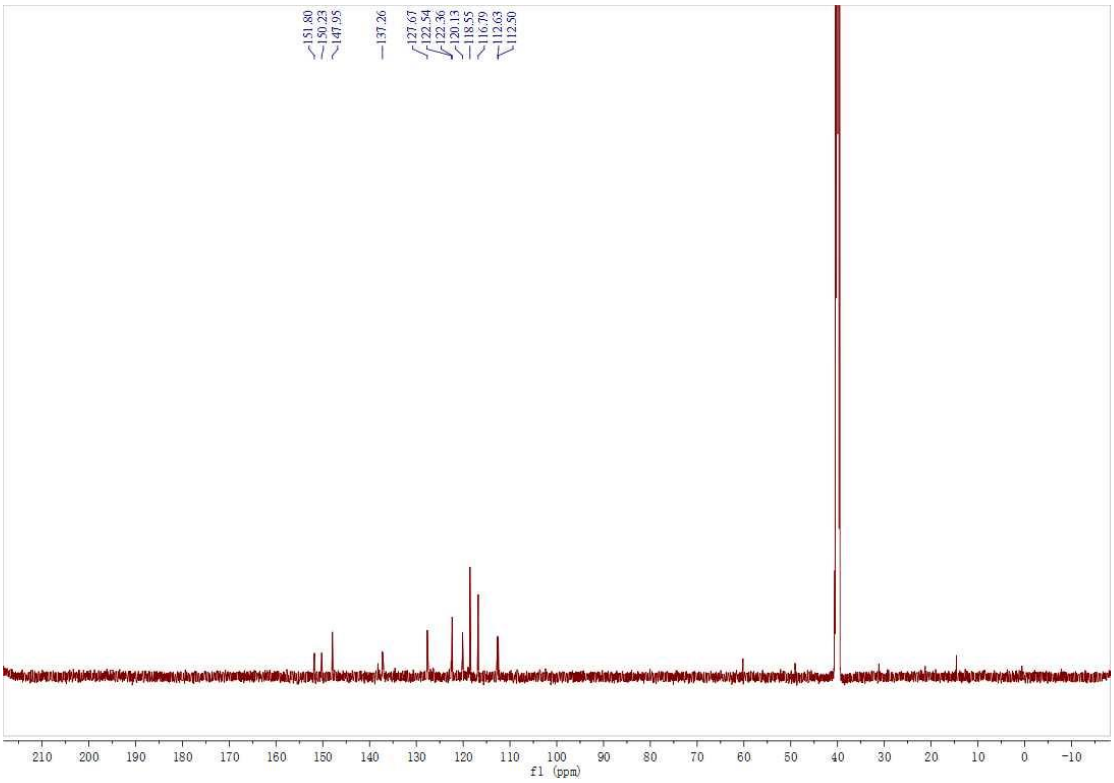

Figure S43. The ^{13}C-NMR of intermediate 14c

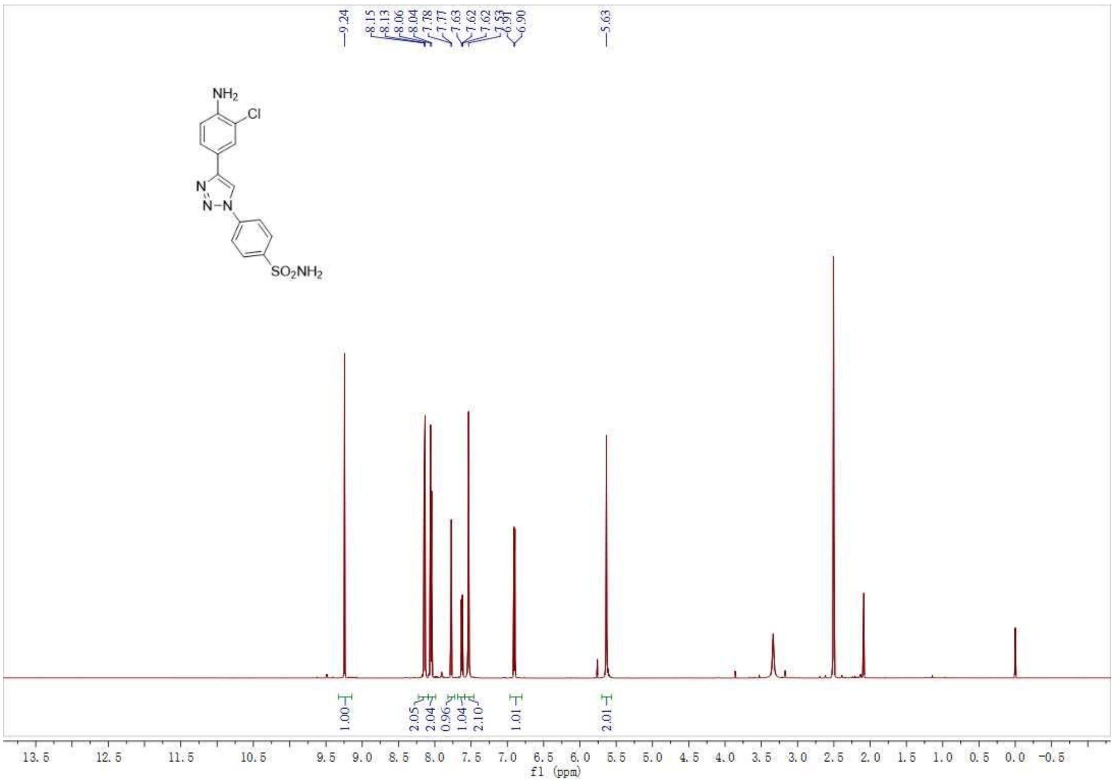

Figure S44. The ^{1}H-NMR of intermediate 14d

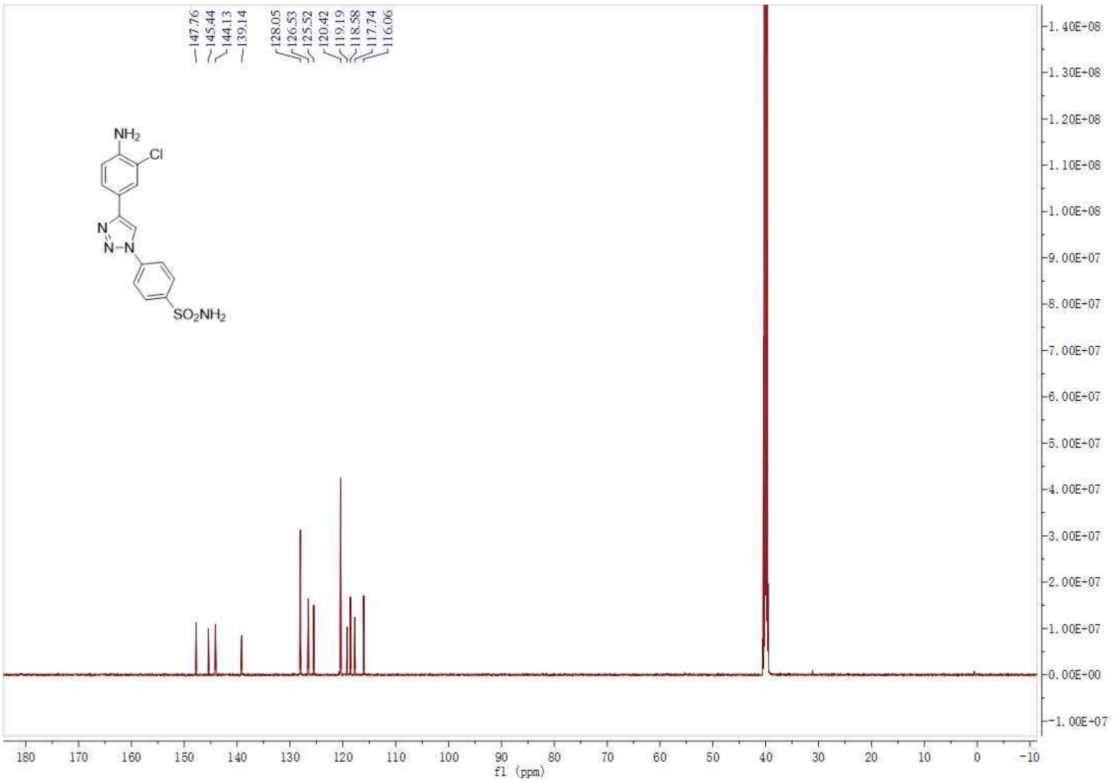

Figure S45. The ^{13}C-NMR of intermediate 14d

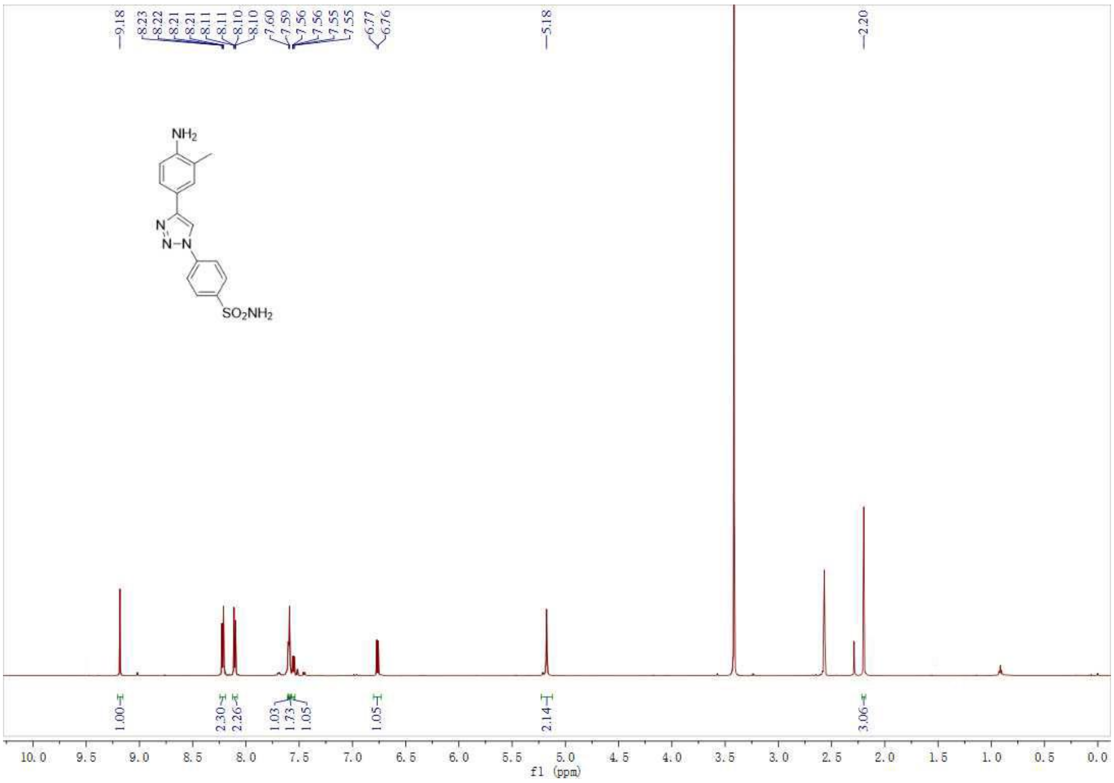

Figure S46. The ^{1}H-NMR of intermediate 14e

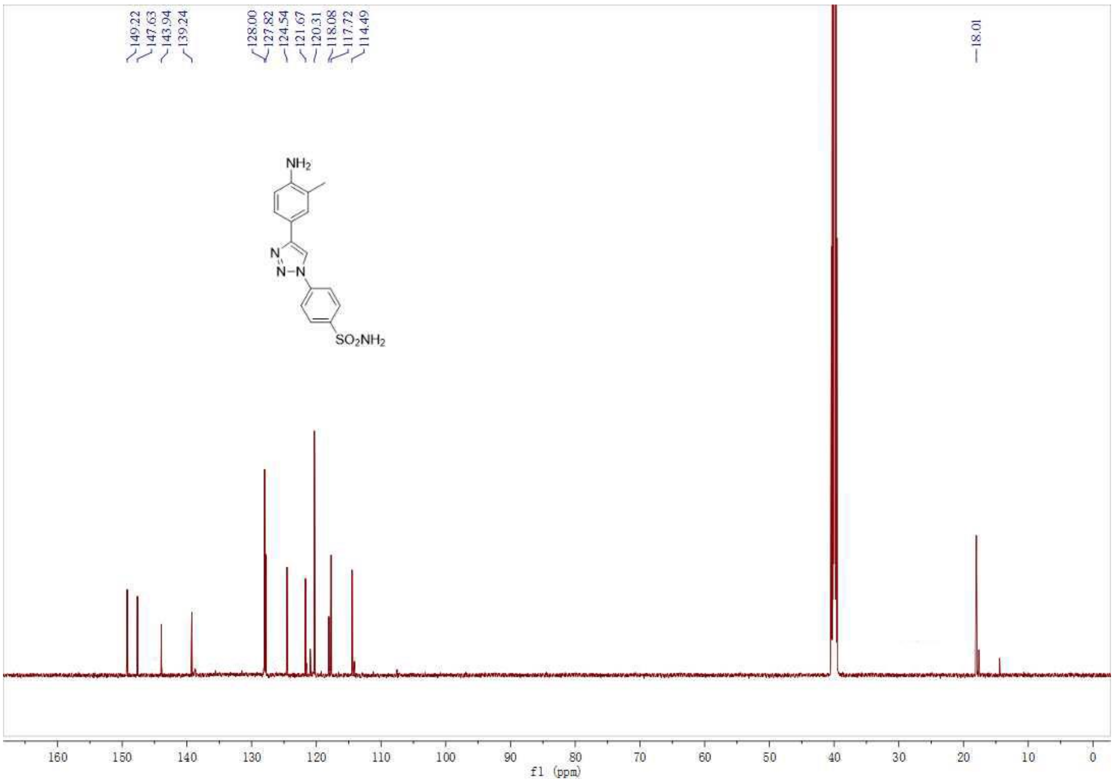

Figure S47. The ^{13}C-NMR of intermediate 14e

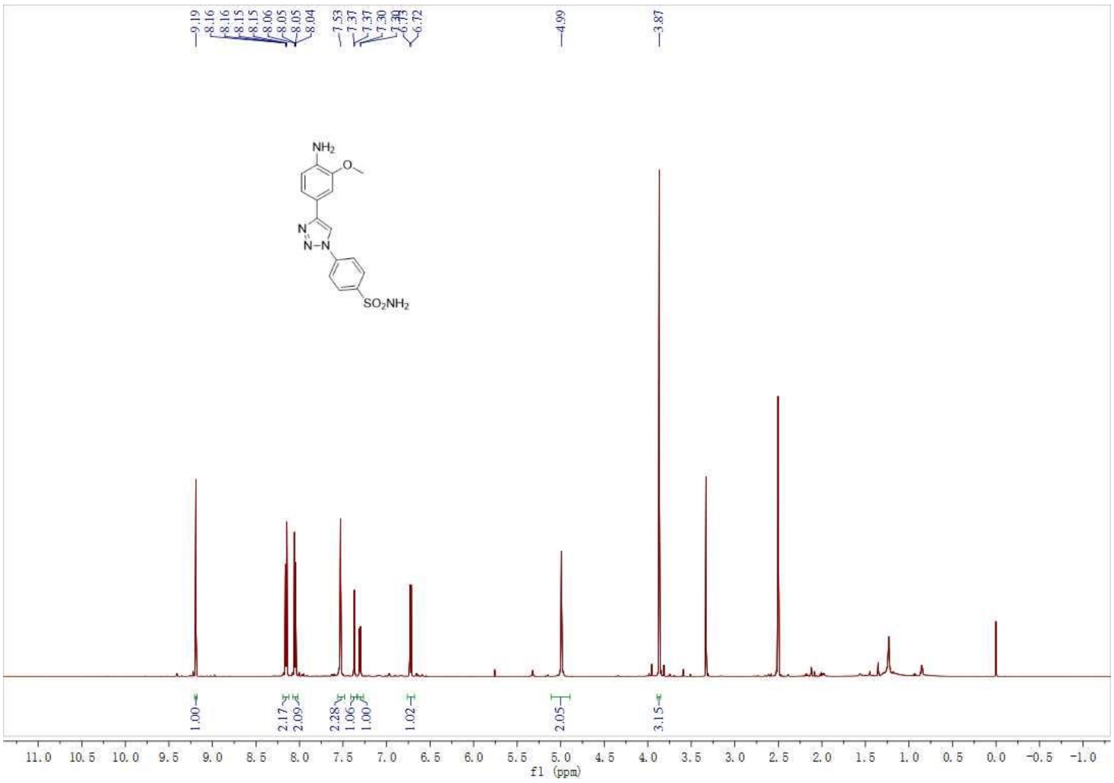

Figure S48. The ^{1}H-NMR of intermediate 14f

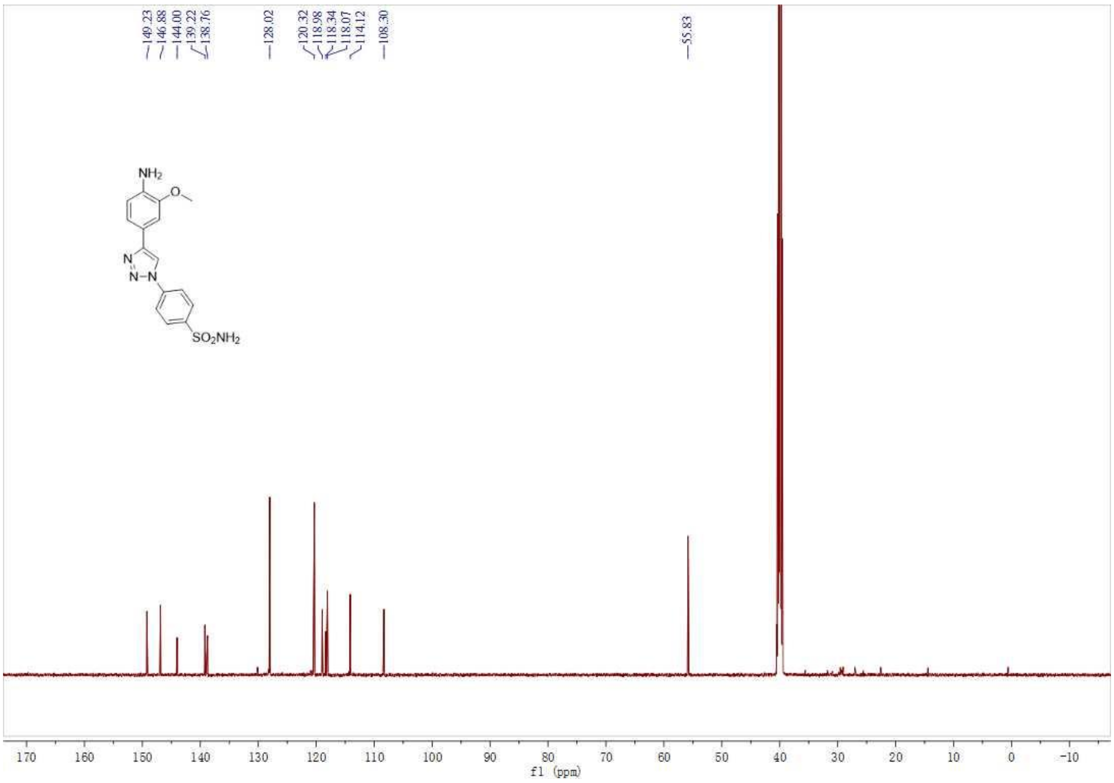

Figure S49. The ^{13}C-NMR of intermediate 14f

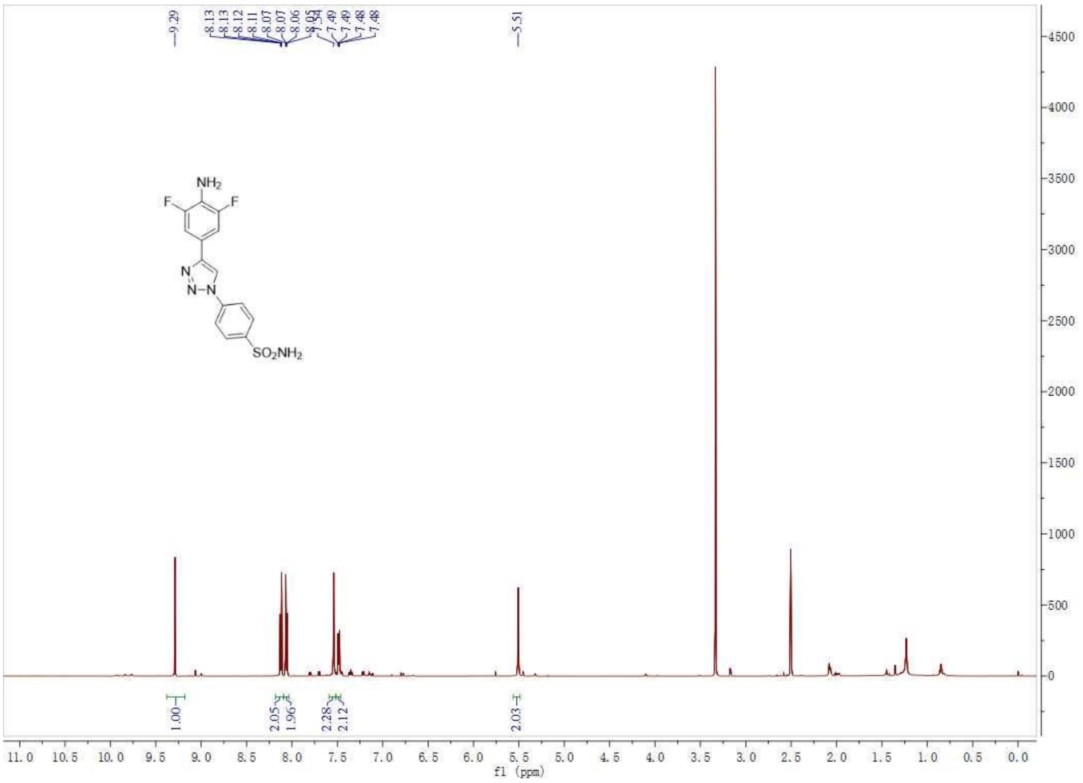

Figure S50. The ^{1}H-NMR of intermediate 14g

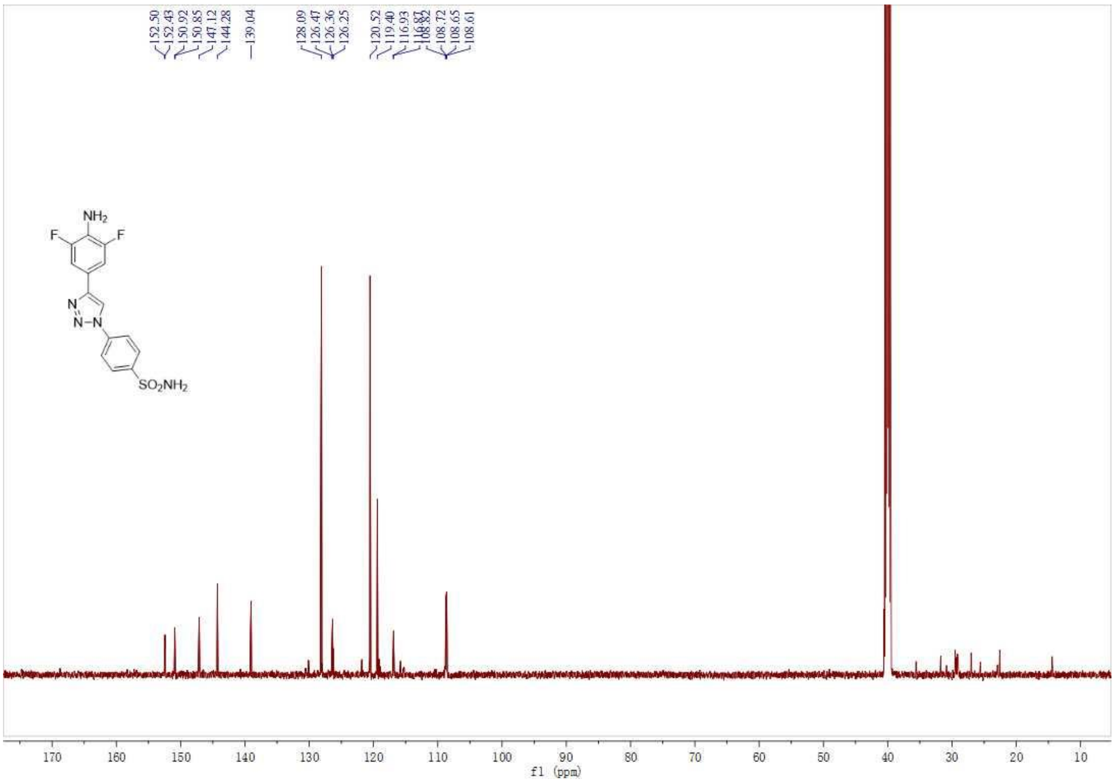

Figure S51. The ^{13}C-NMR of intermediate 14g

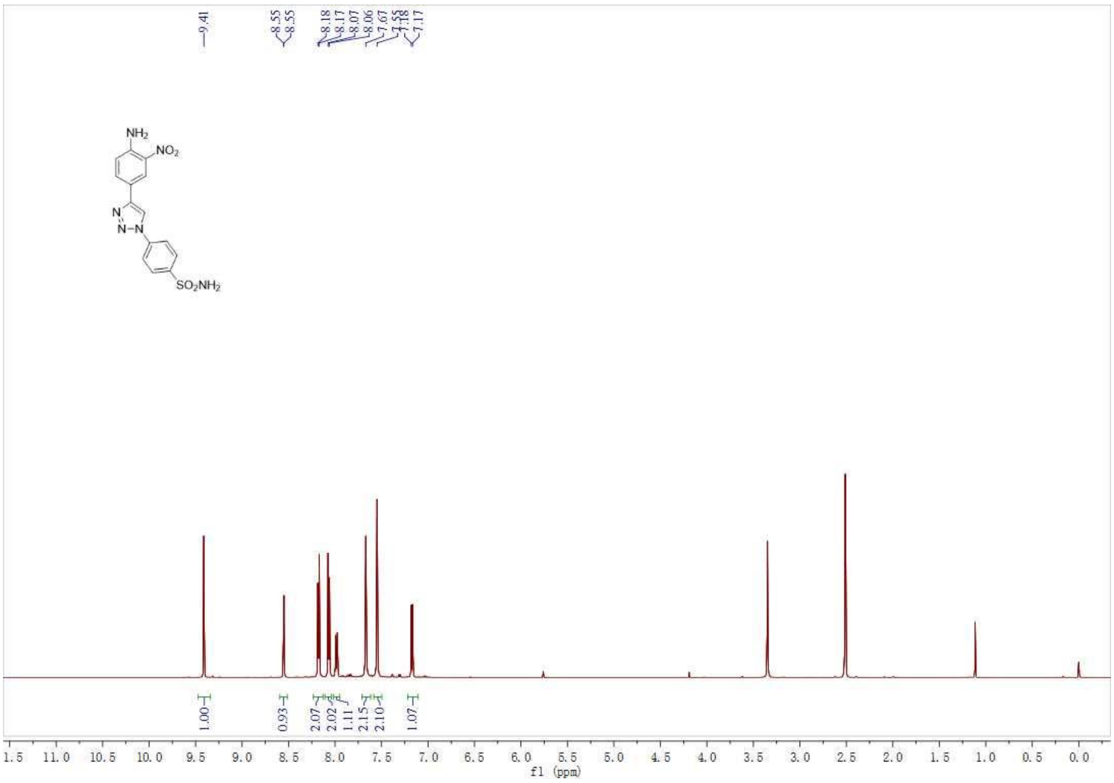

Figure S52. The ^{1}H-NMR of intermediate 14h

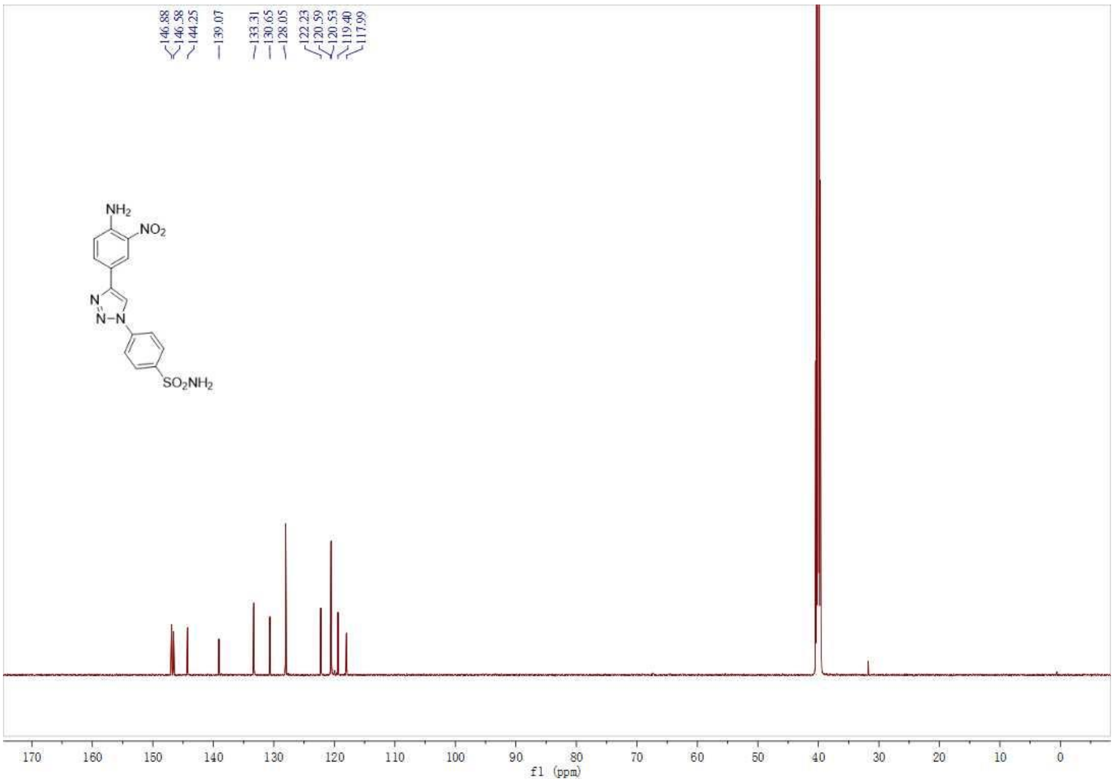

Figure S53. The ^{13}C-NMR of intermediate 14h

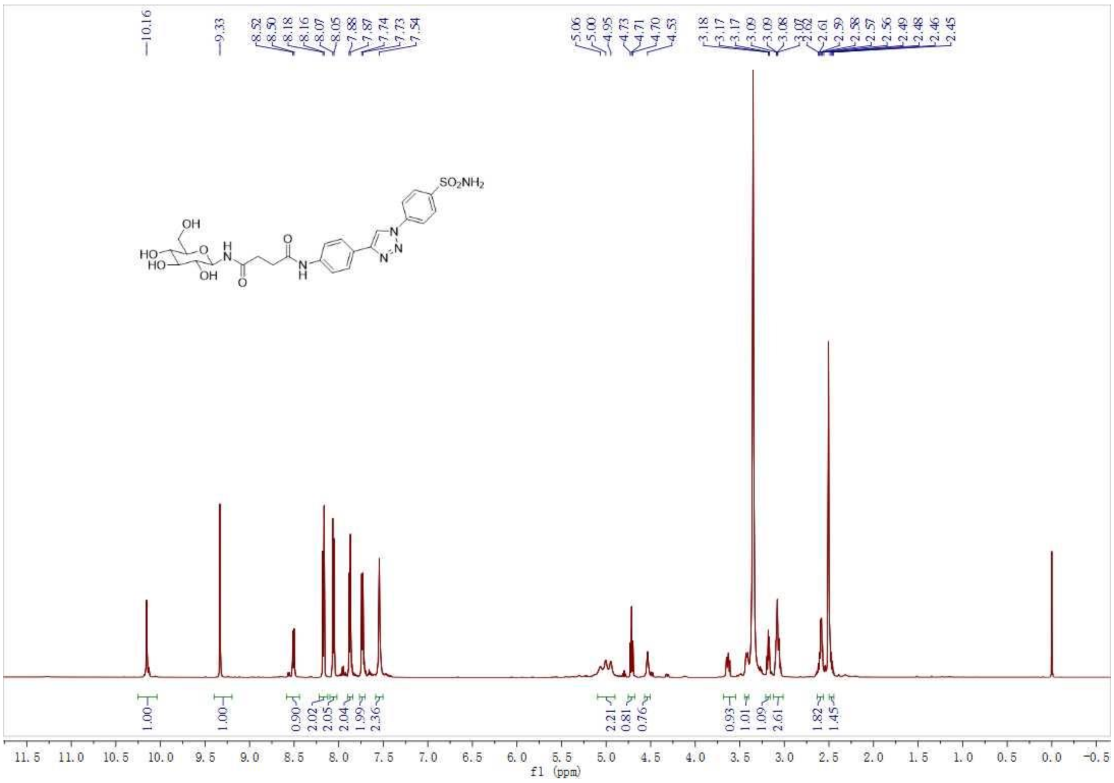

Figure S54. The ^{1}H-NMR of compound 16a

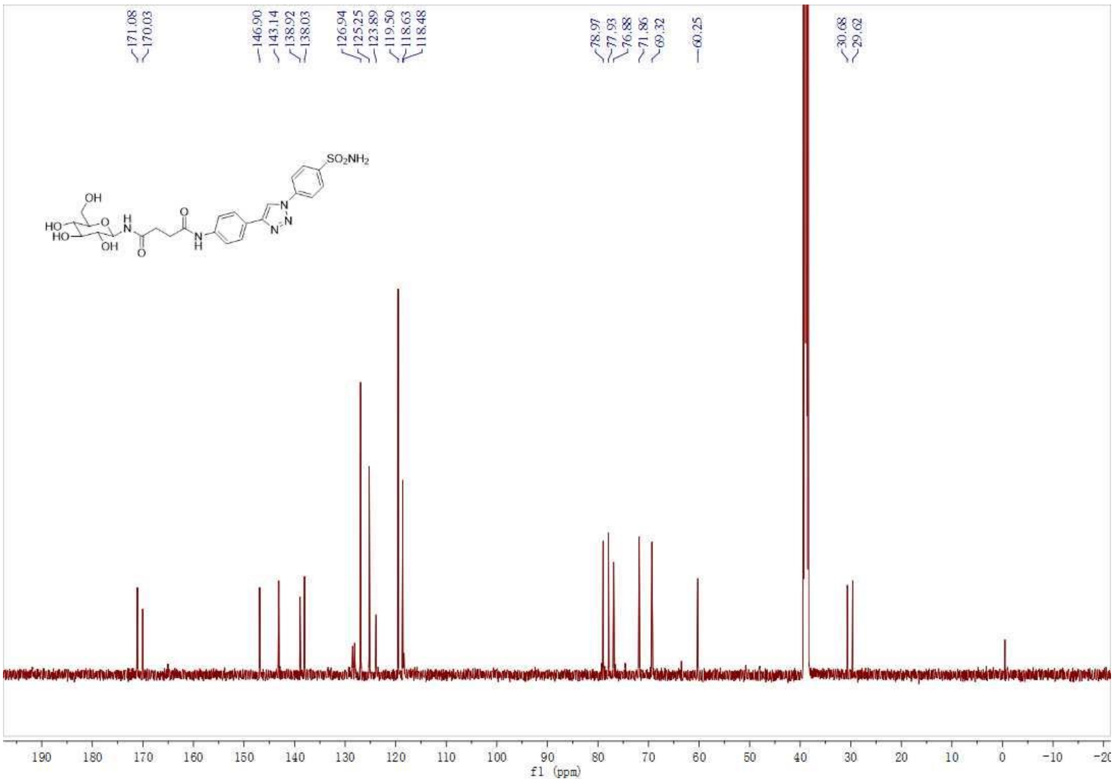

Figure S55. The ^{13}C-NMR of compound 16a

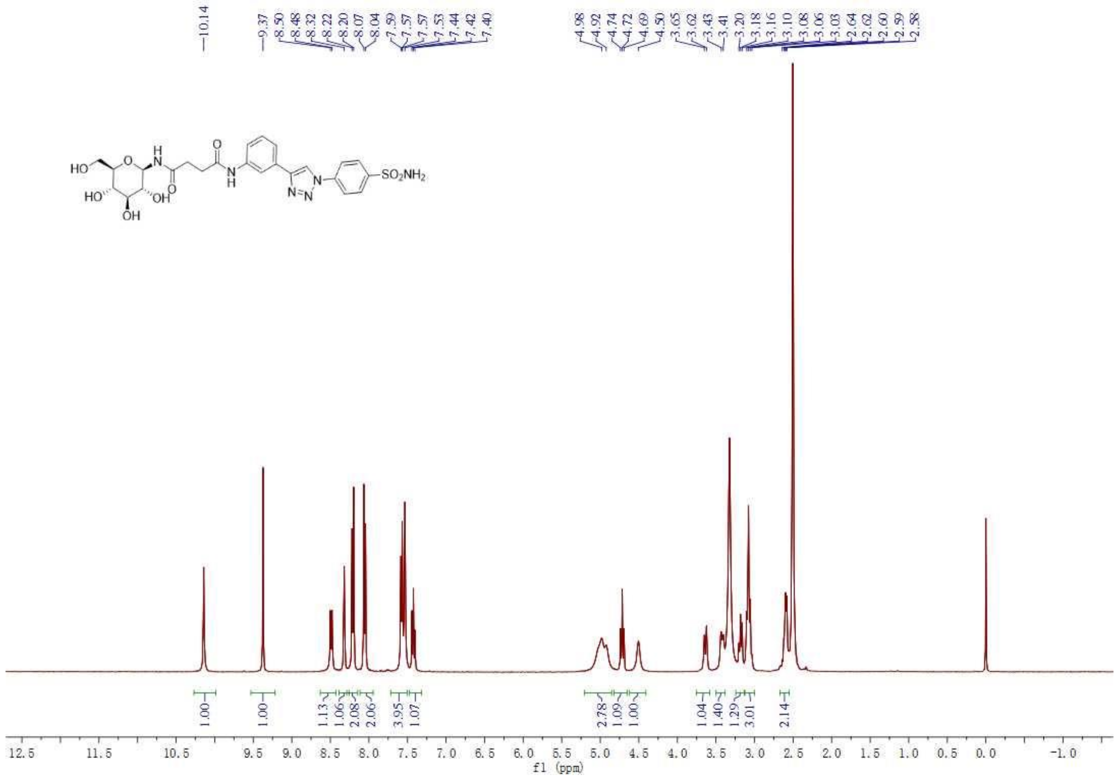

Figure S56. The ^{1}H-NMR of compound 16b

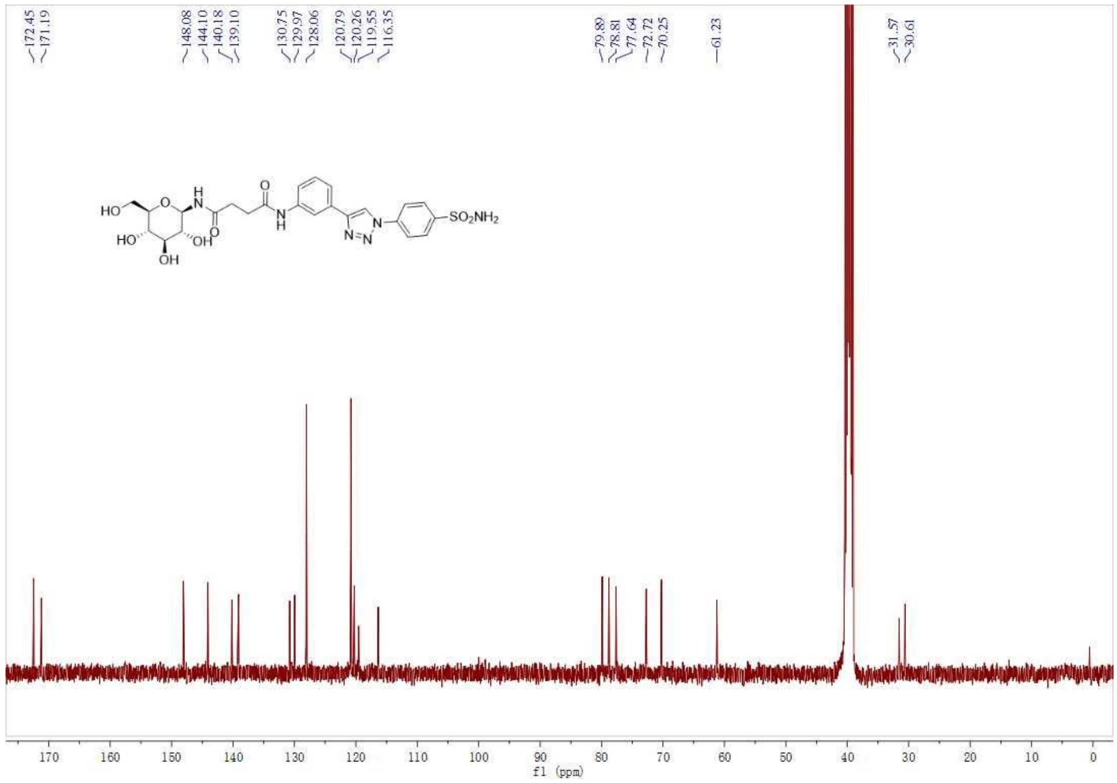

Figure S57. The ^{13}C-NMR of compound 16b

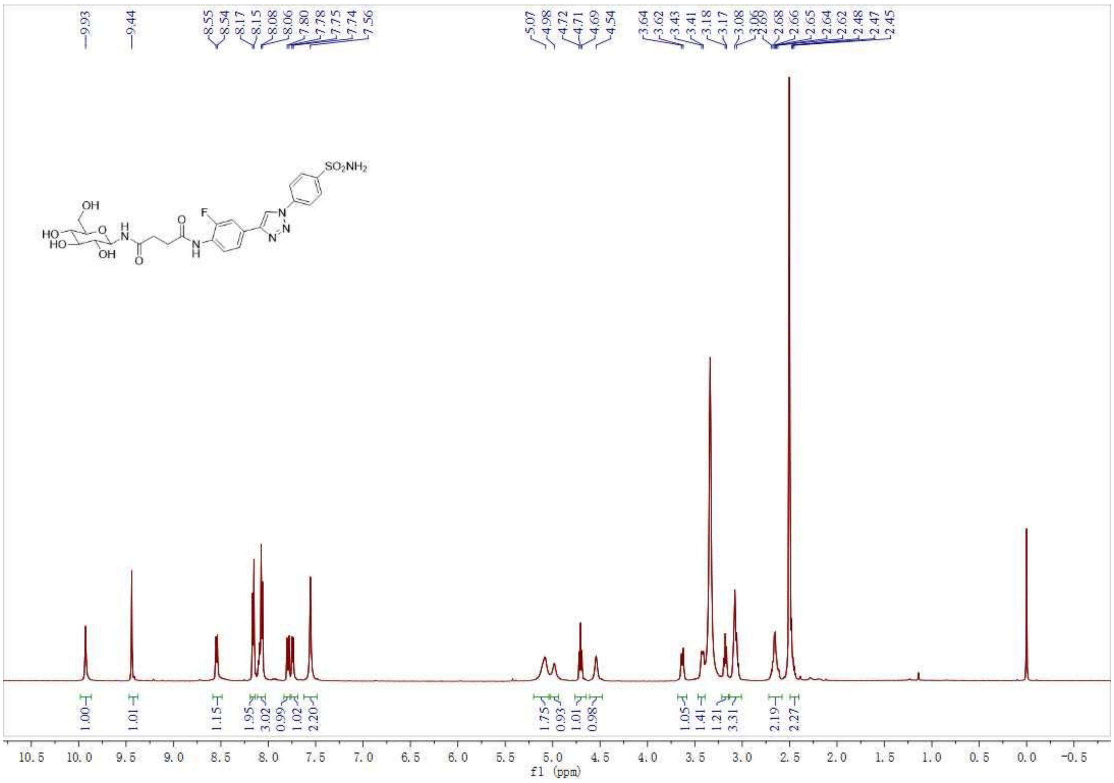

Figure S58. The ^1H-NMR of compound 16c

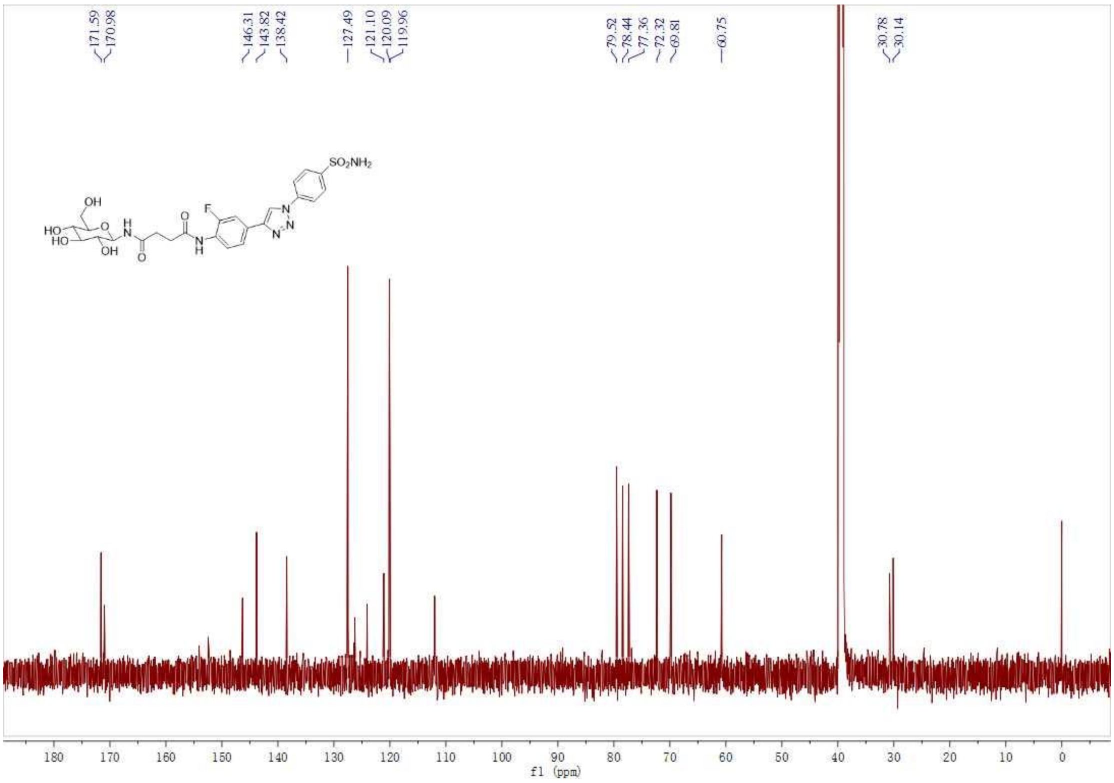

Figure S59. The ^{13}C-NMR of compound 16c

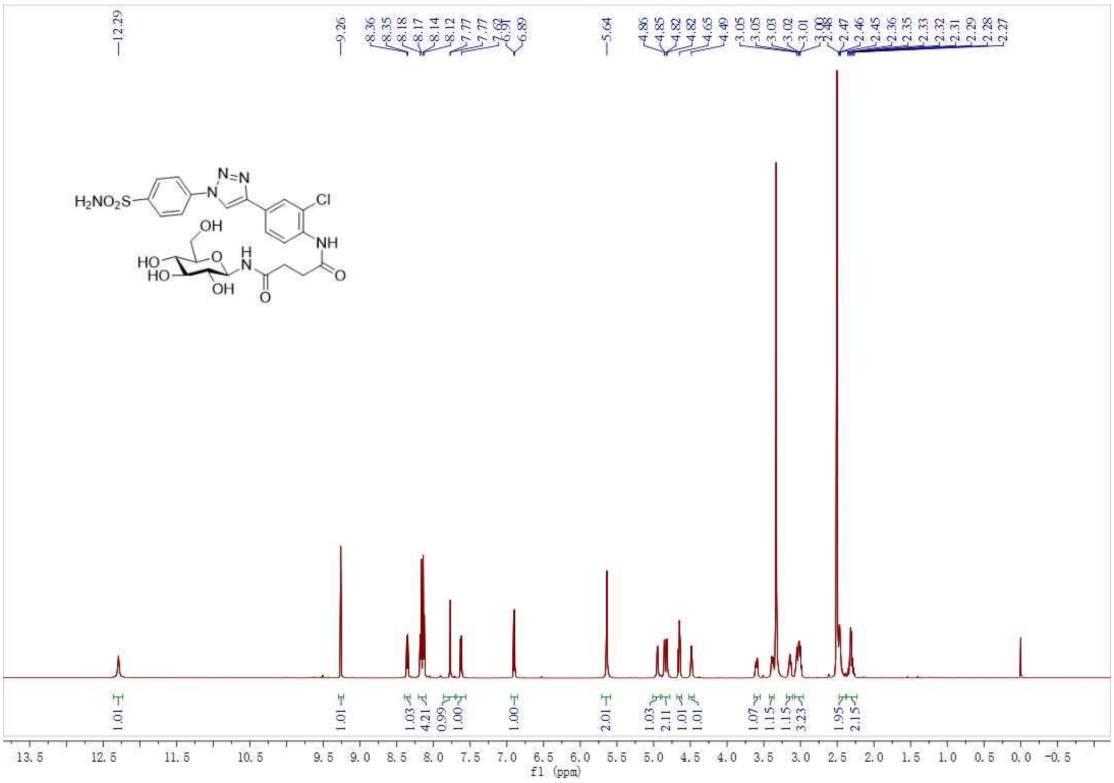

Figure S60. The ^{1}H-NMR of compound 16d

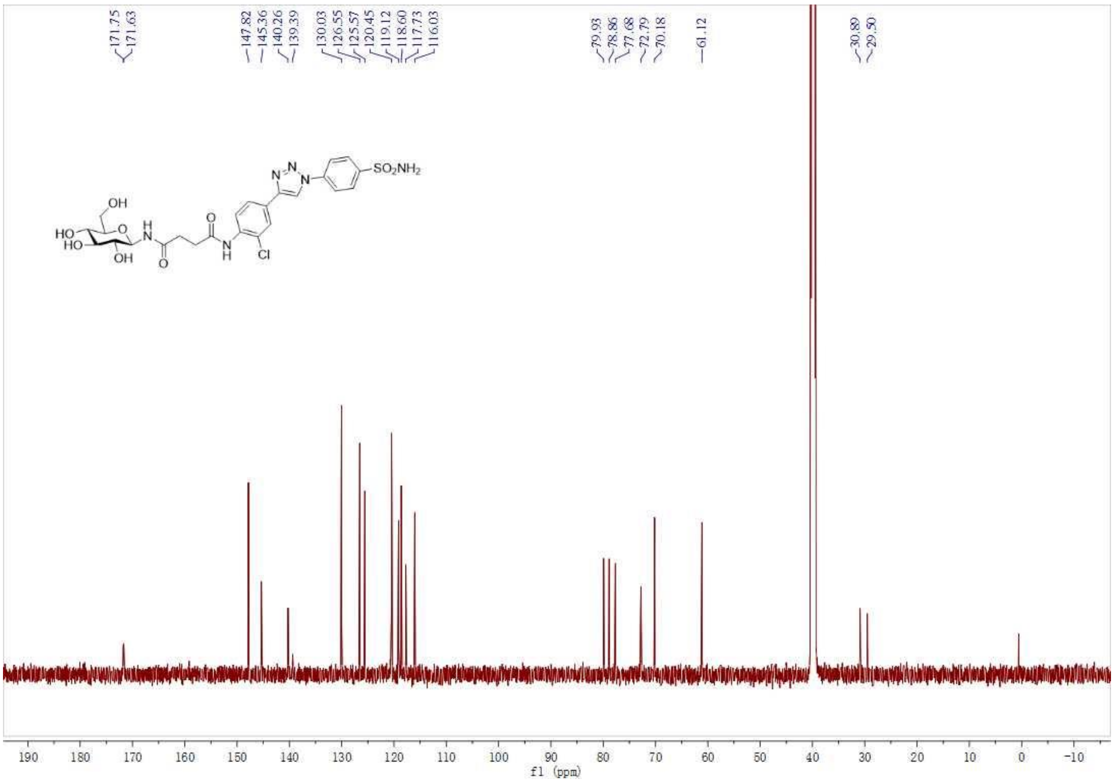

Figure S61. The ^{13}C-NMR of compound 16d

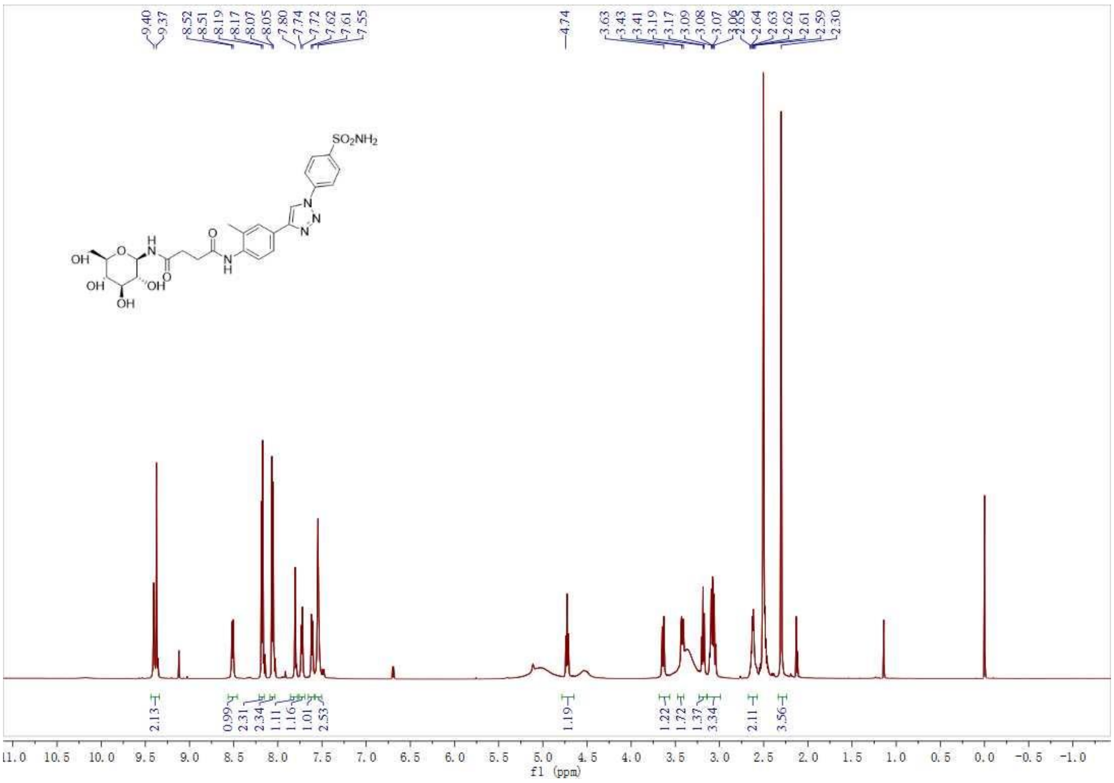

Figure S62. The ^{1}H-NMR of compound 16e

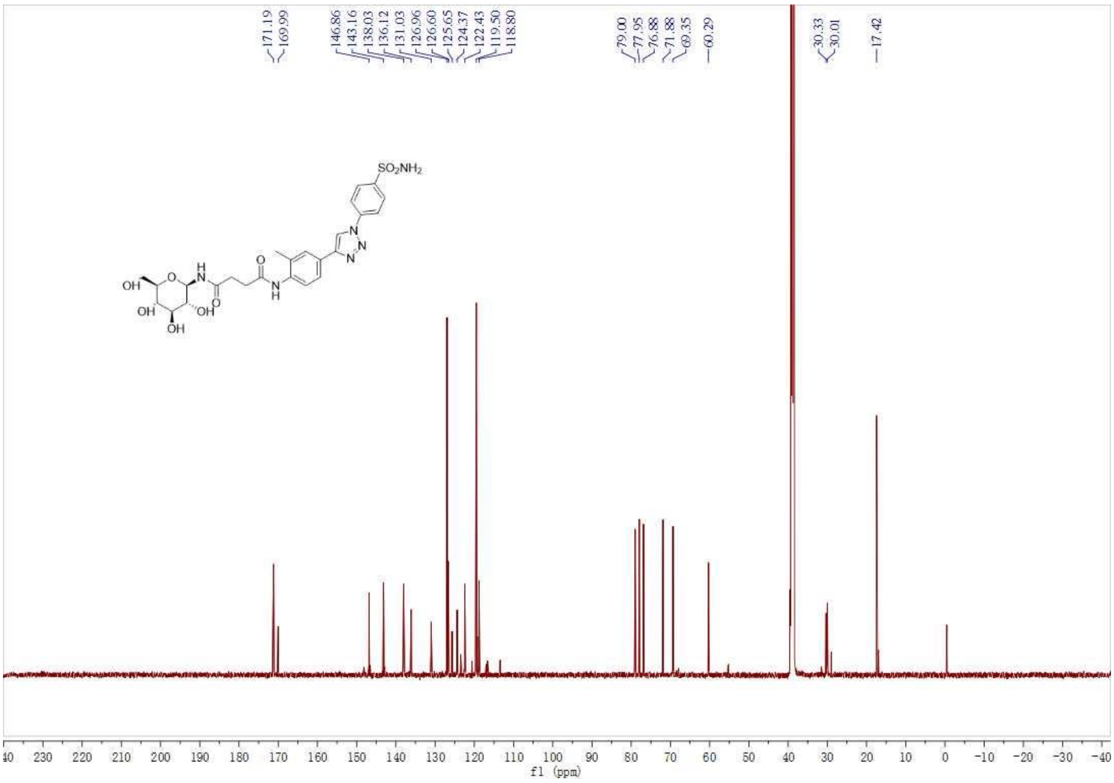

Figure S63. The ^{13}C-NMR of compound 16e

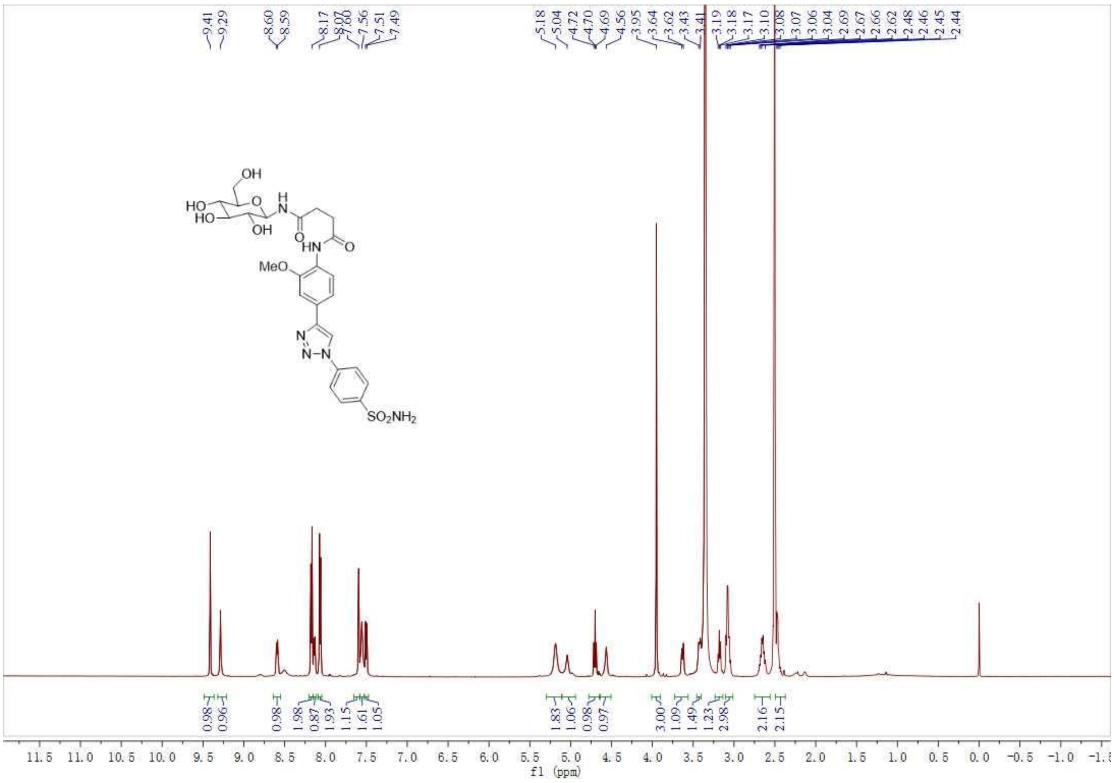

Figure S64. The ^{1}H-NMR of compound 16f

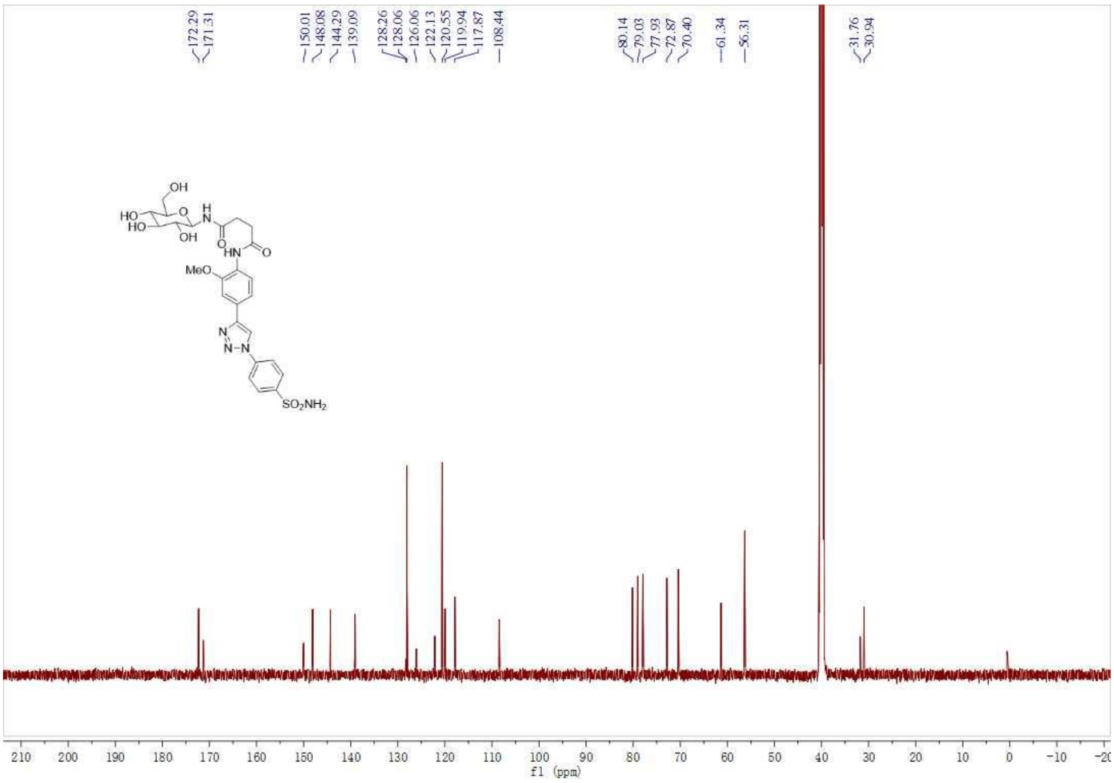

Figure S65. The ^{13}C-NMR of compound 16f

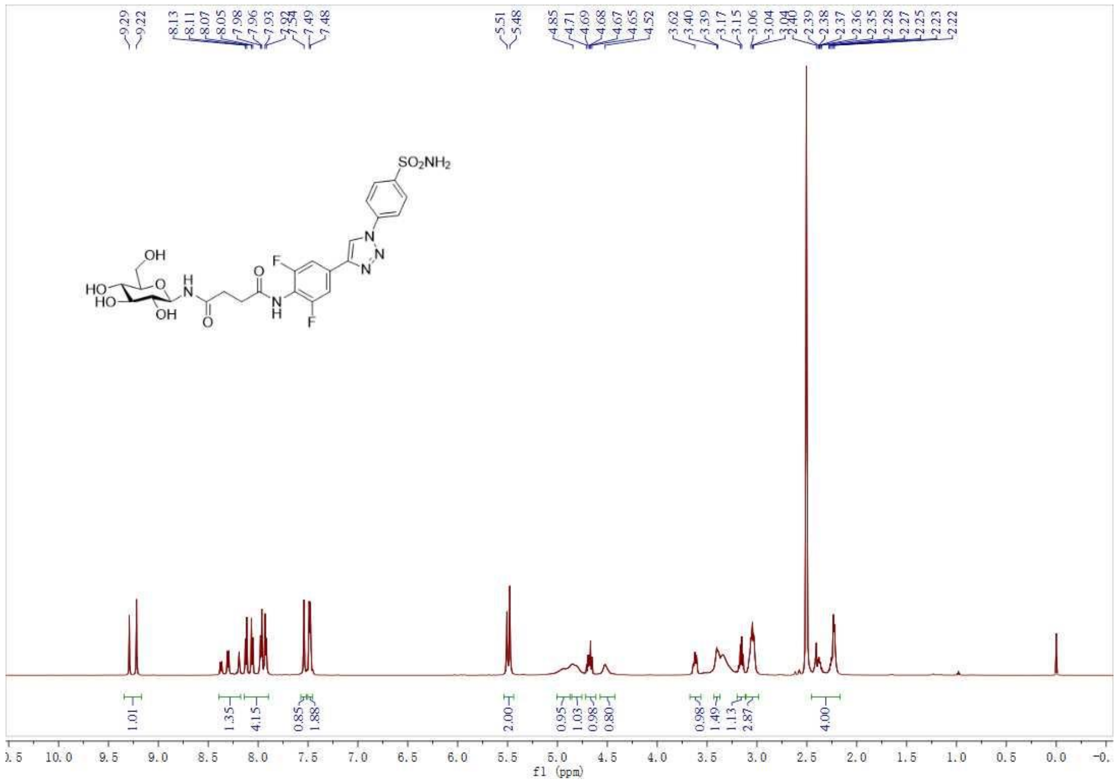

Figure S66. The ^{1}H-NMR of compound 16g

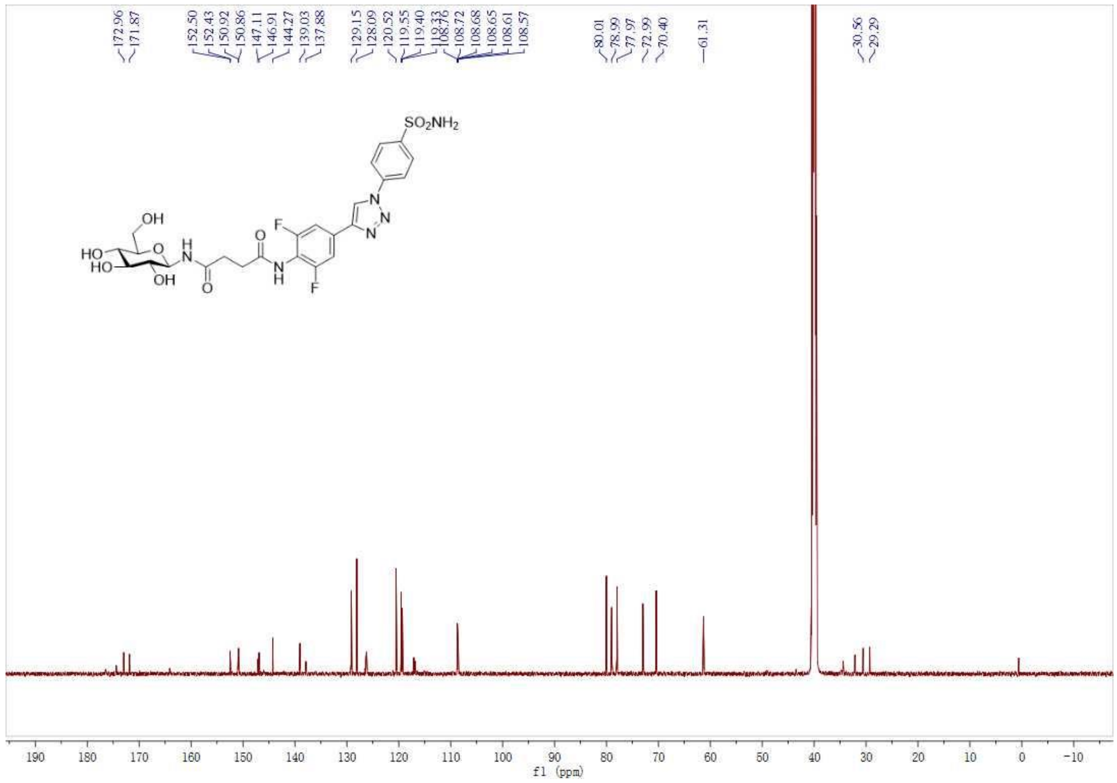

Figure S67. The ^{13}C-NMR of compound 16g

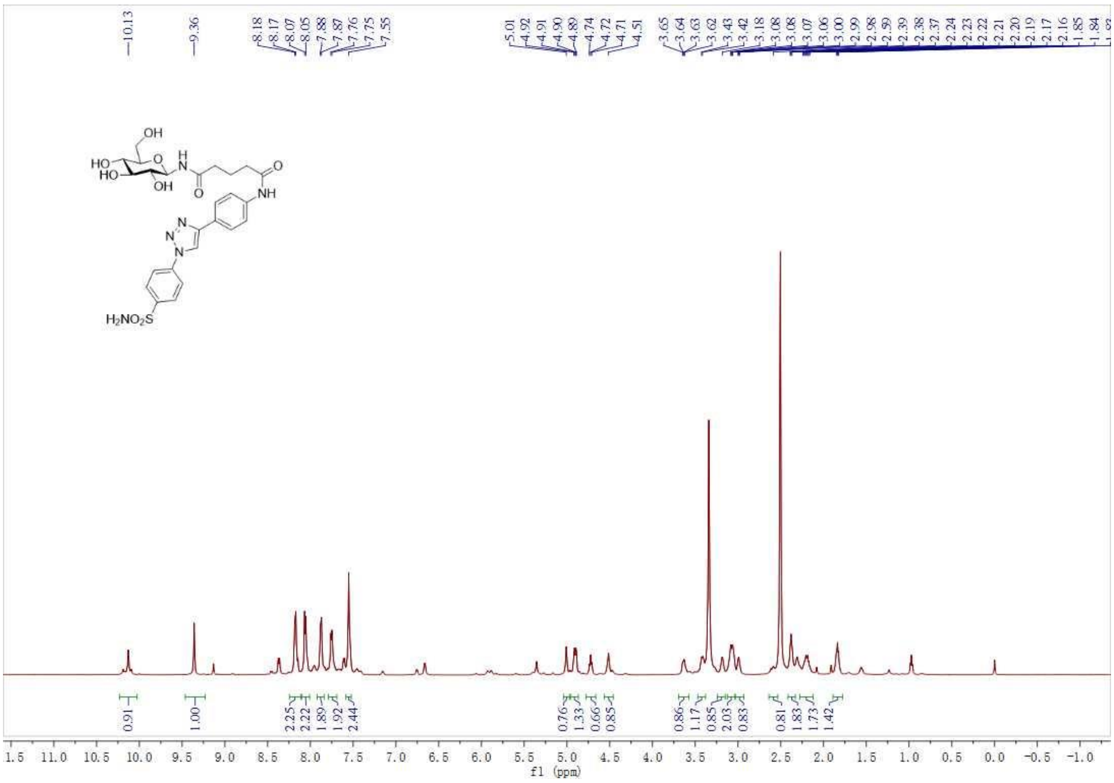

Figure S68. The ^{1}H-NMR of compound 16h

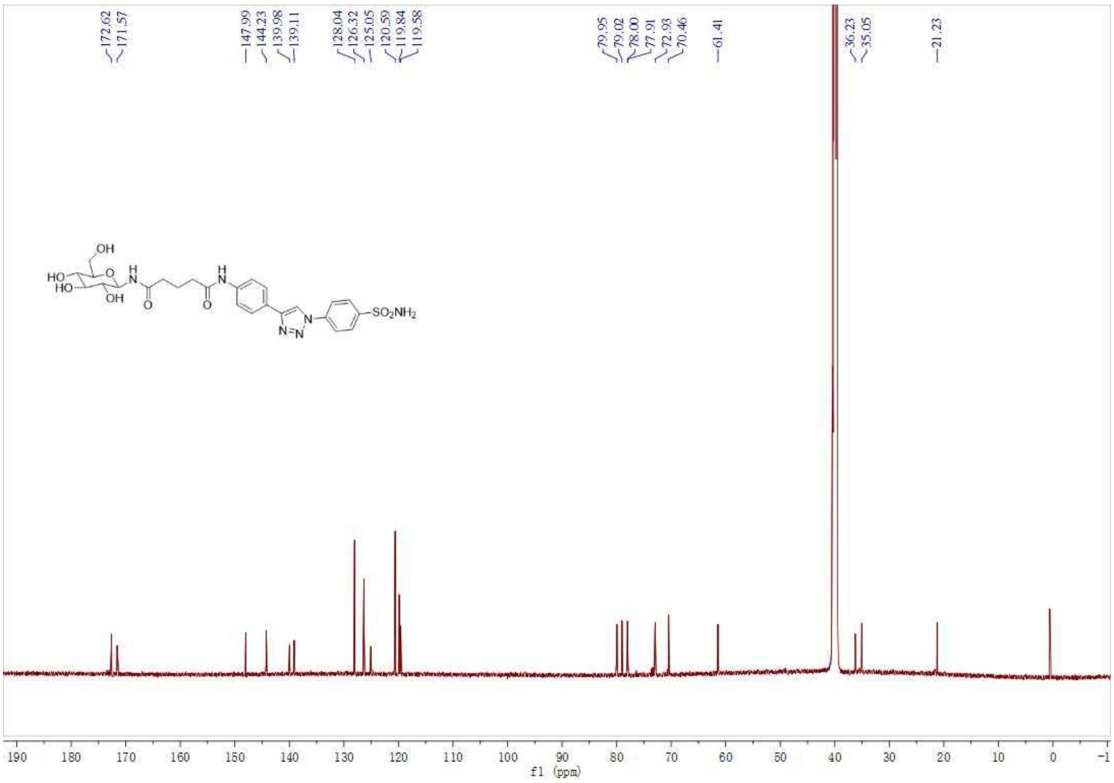

Figure S69. The ^{13}C-NMR of compound 16h

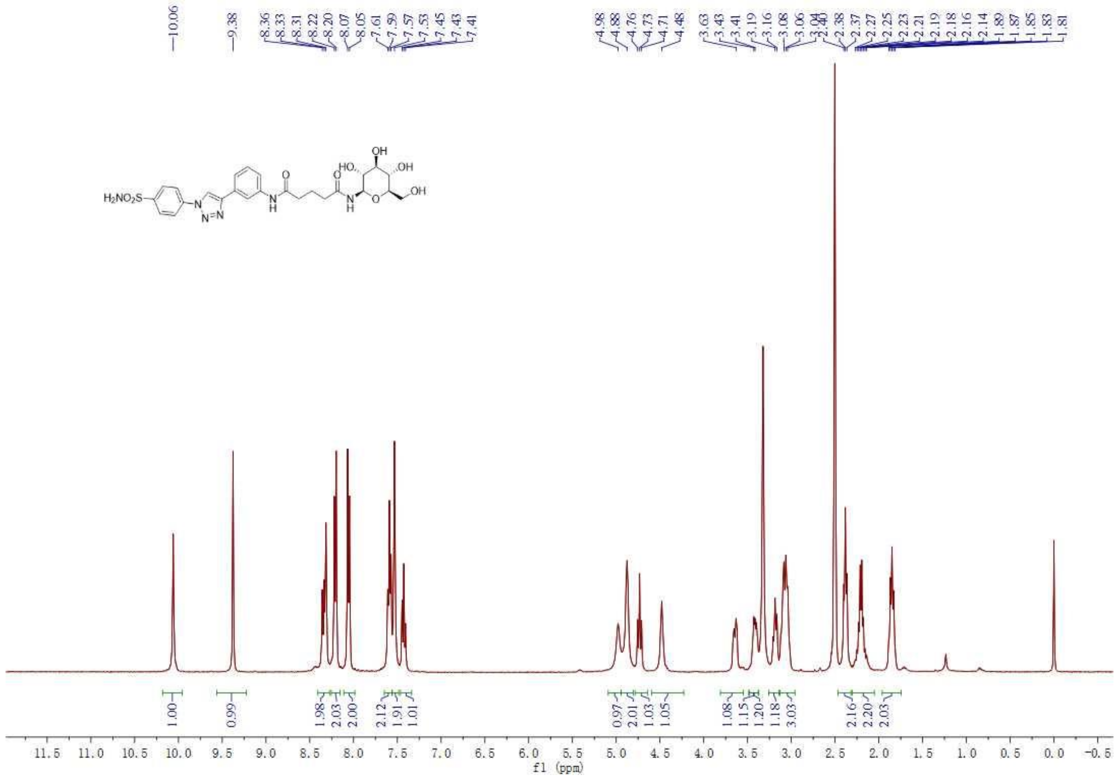

Figure S70. The ^{1}H-NMR of compound 16i

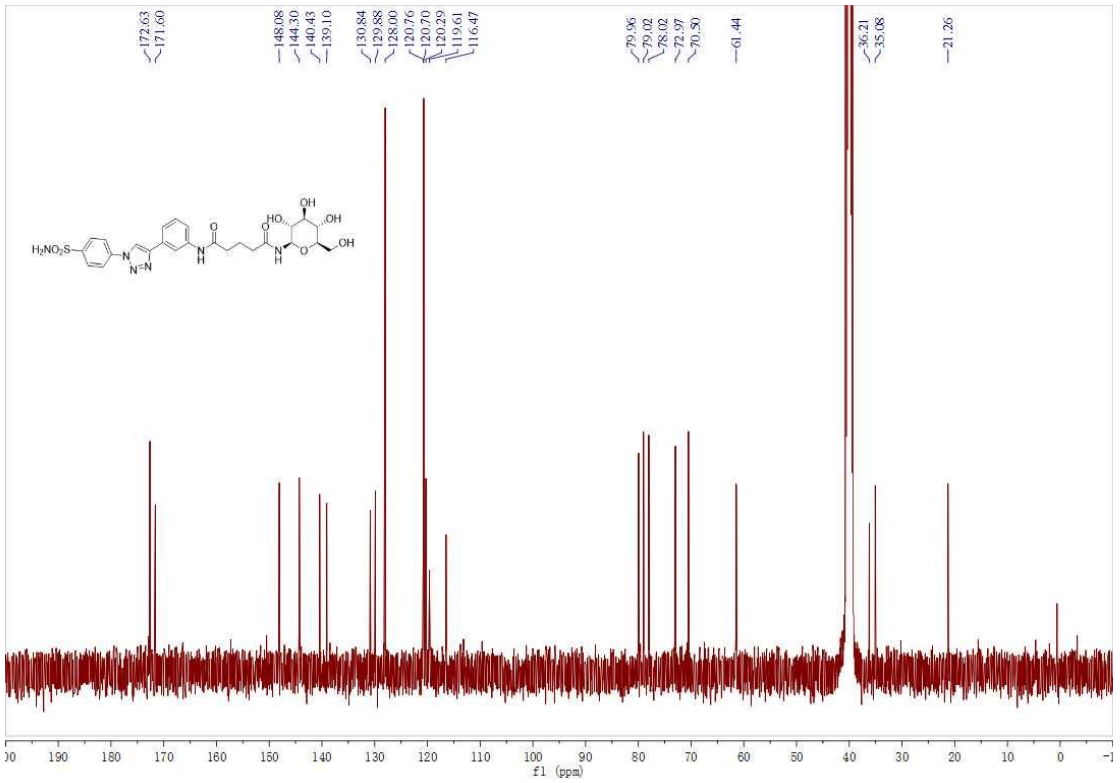

Figure S71. The ^{13}C-NMR of compound 16i

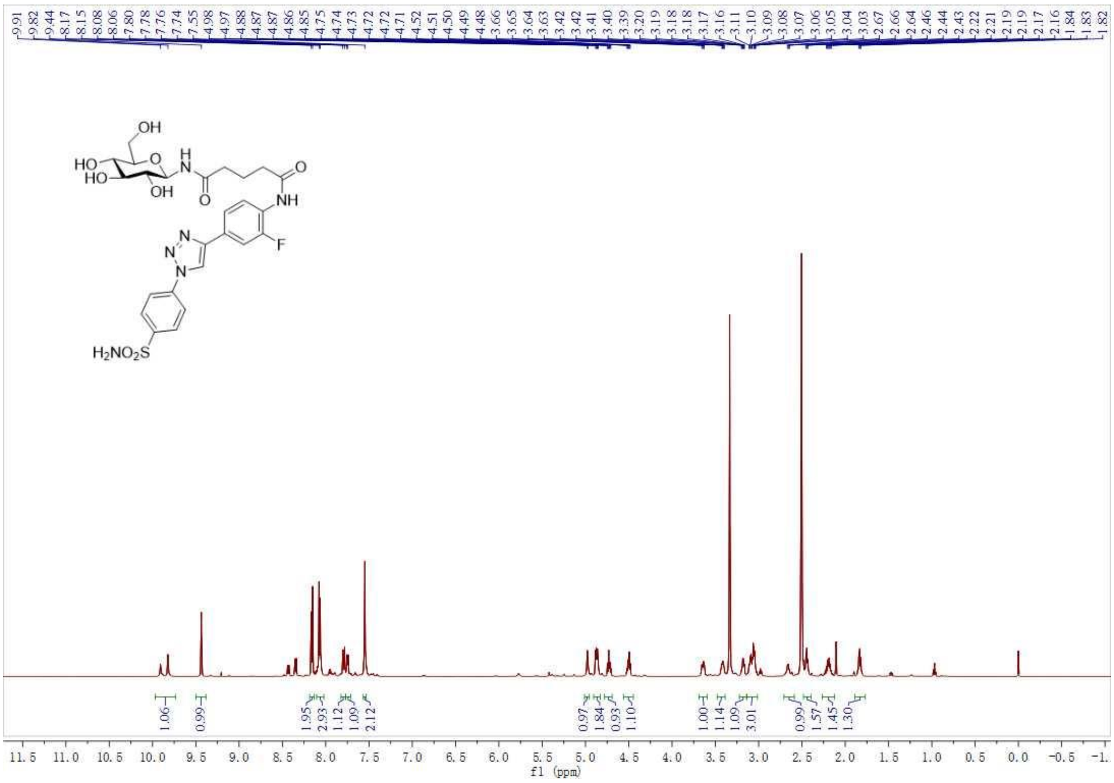

Figure S72. The ^{1}H-NMR of compound 16j

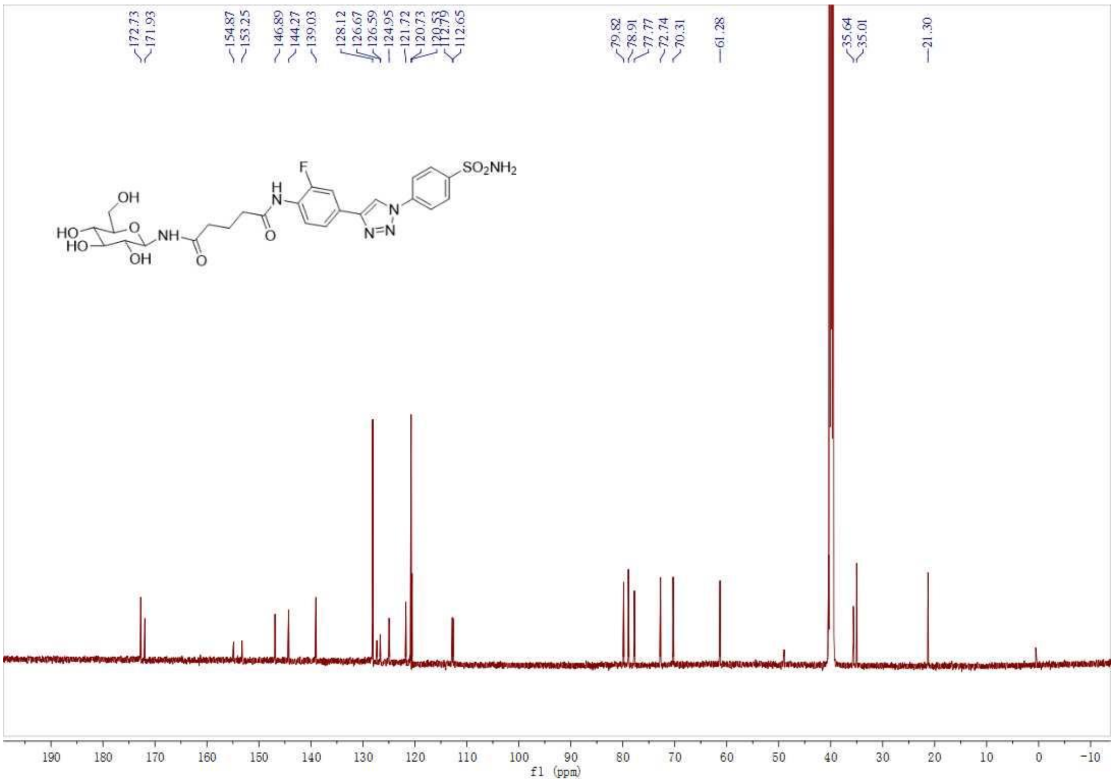

Figure S73. The ^{13}C-NMR of compound 16j

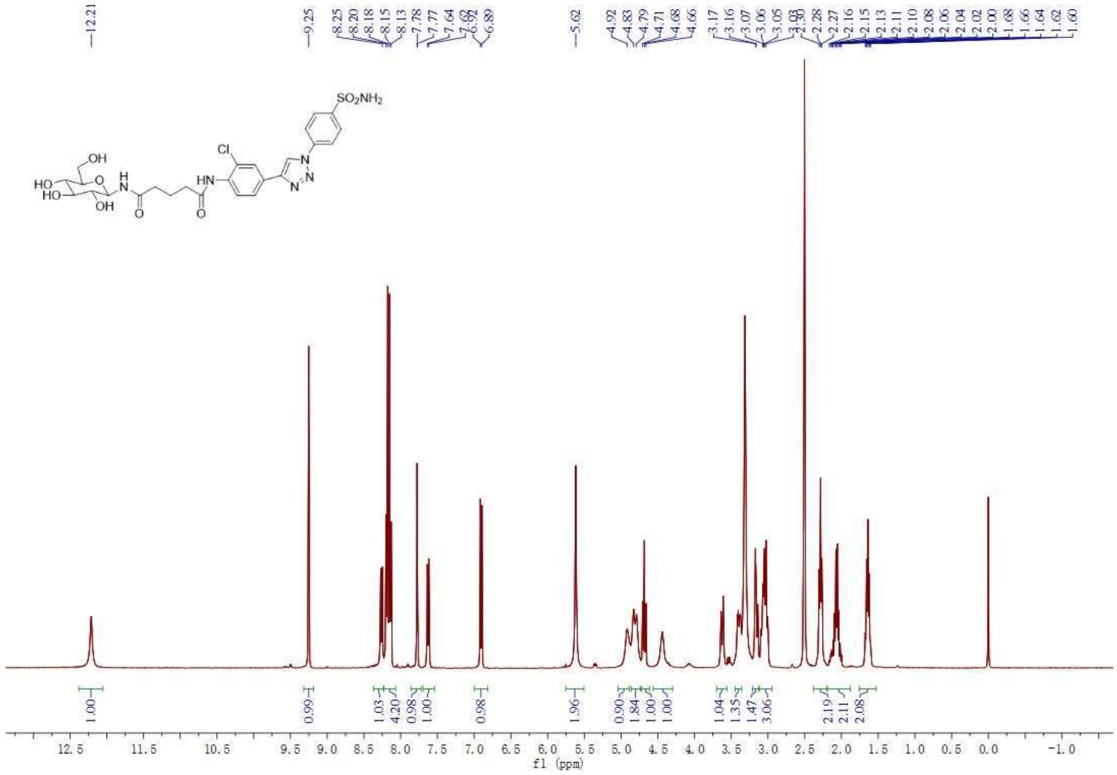

Figure S74. The ^{1}H-NMR of compound 16k

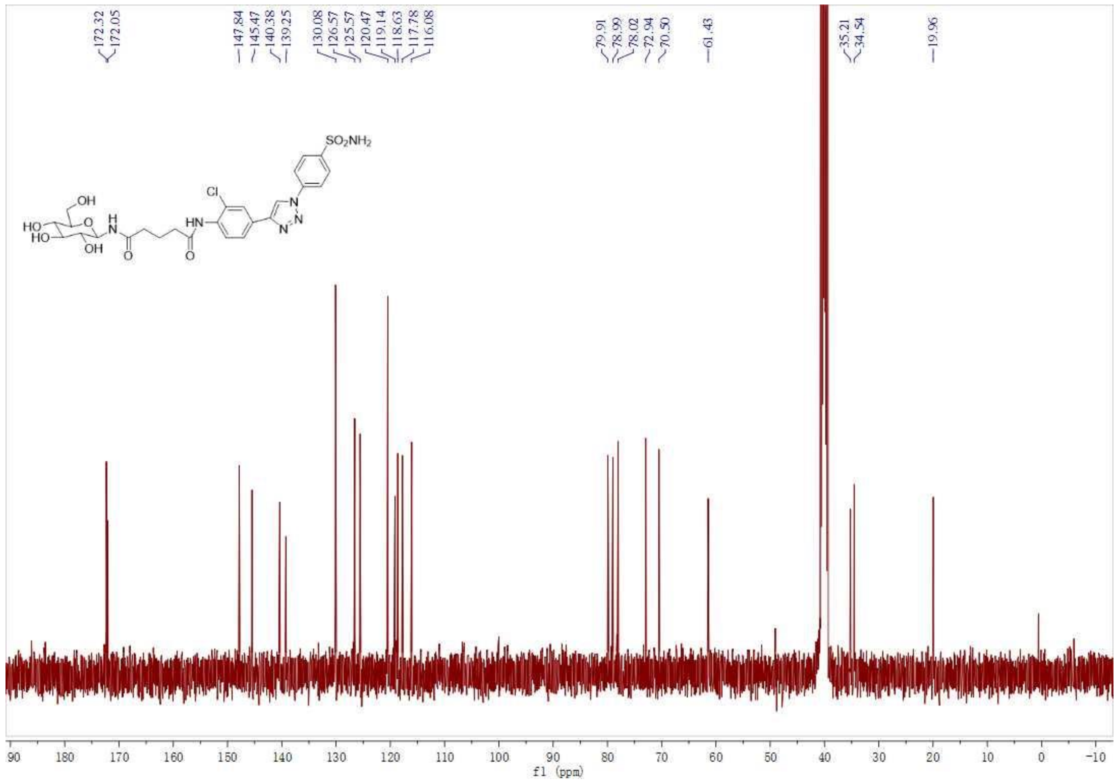

Figure S75. The ^{13}C-NMR of compound 16k

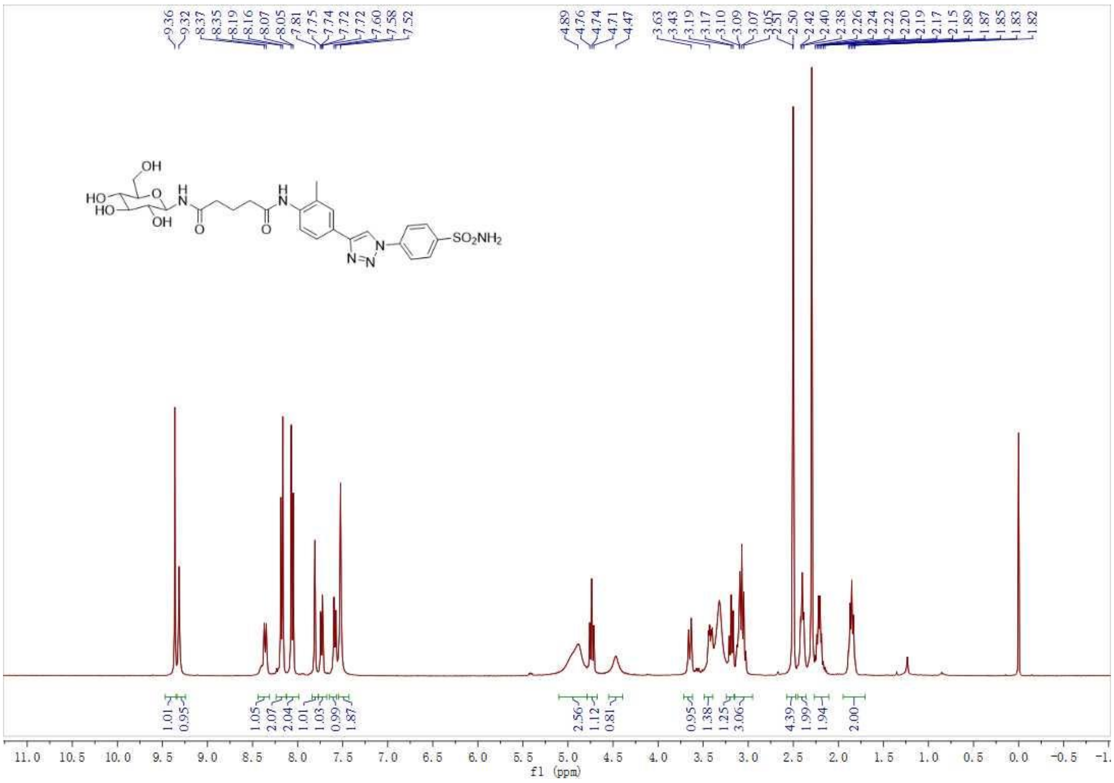

Figure S76. The ^{1}H-NMR of compound 16l

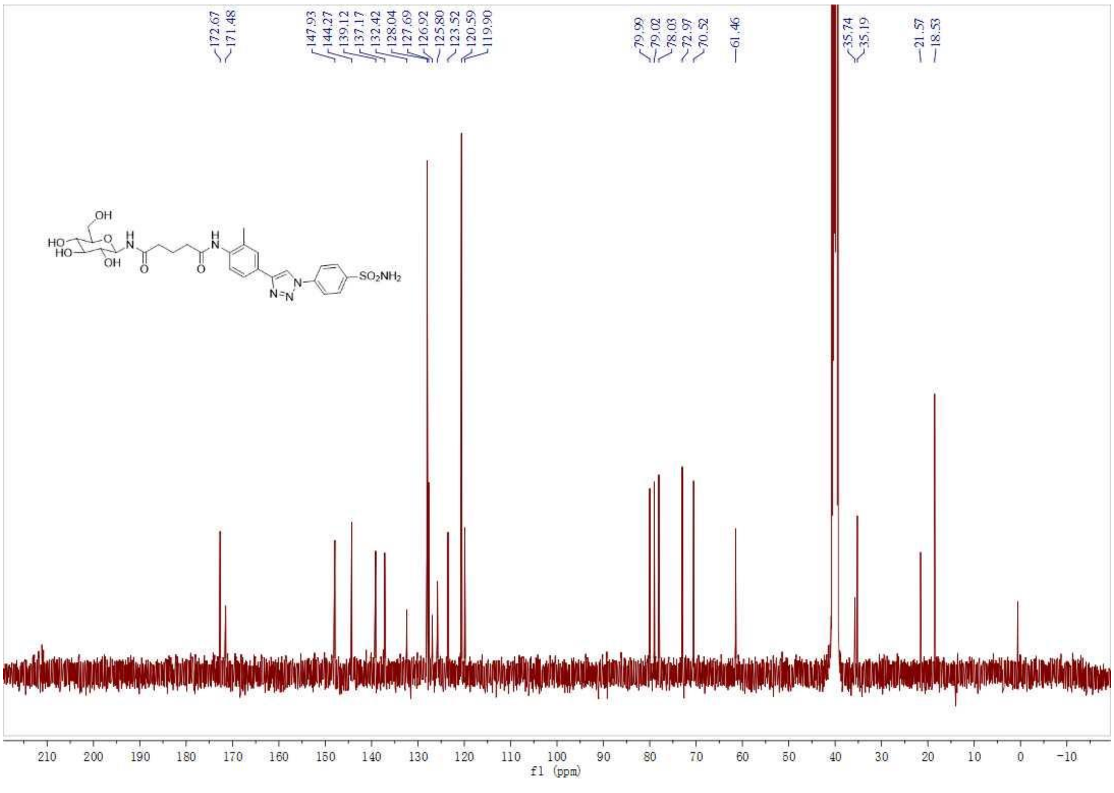

Figure S77. The ^{13}C-NMR of compound 16l

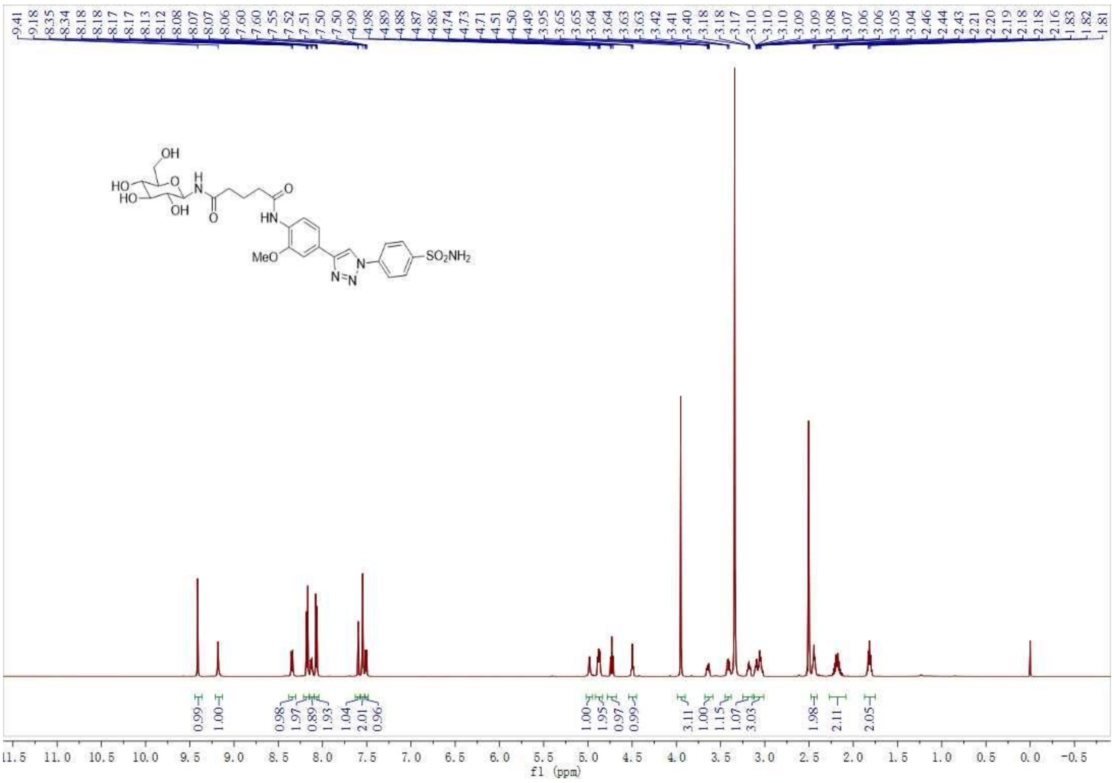

Figure S78. The ^{1}H-NMR of compound 16m

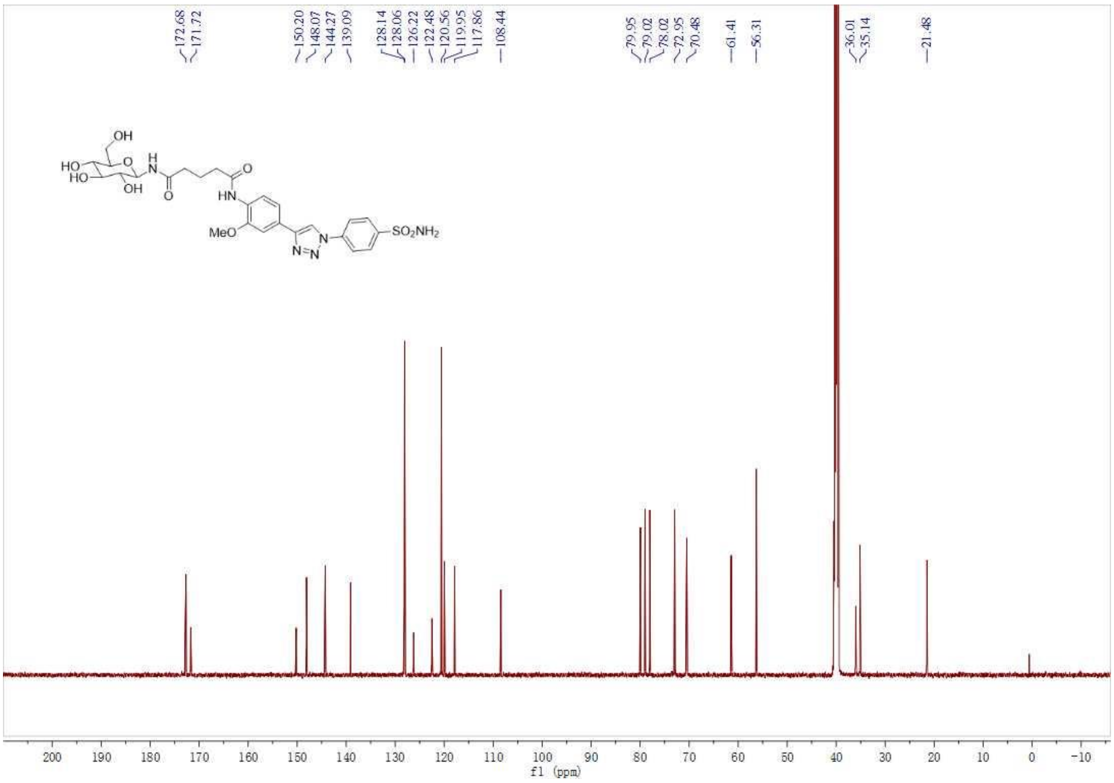

Figure S79. The ^{13}C-NMR of compound 16m

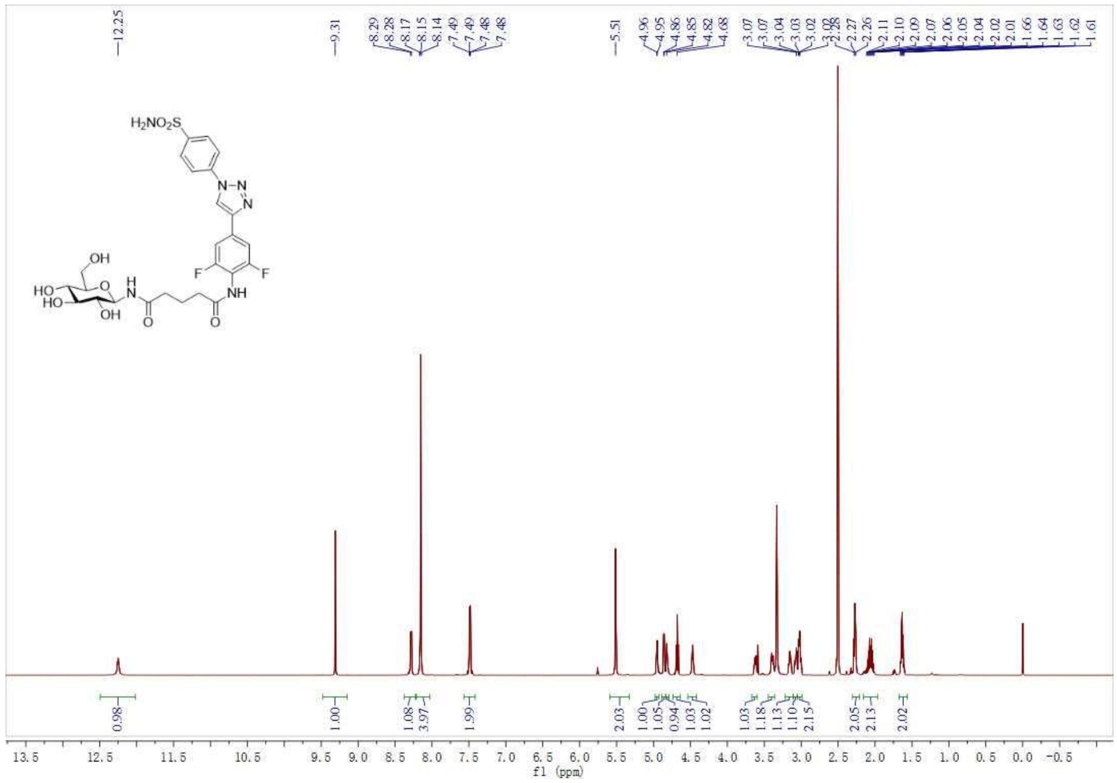

Figure S80. The ^{1}H-NMR of compound 16n

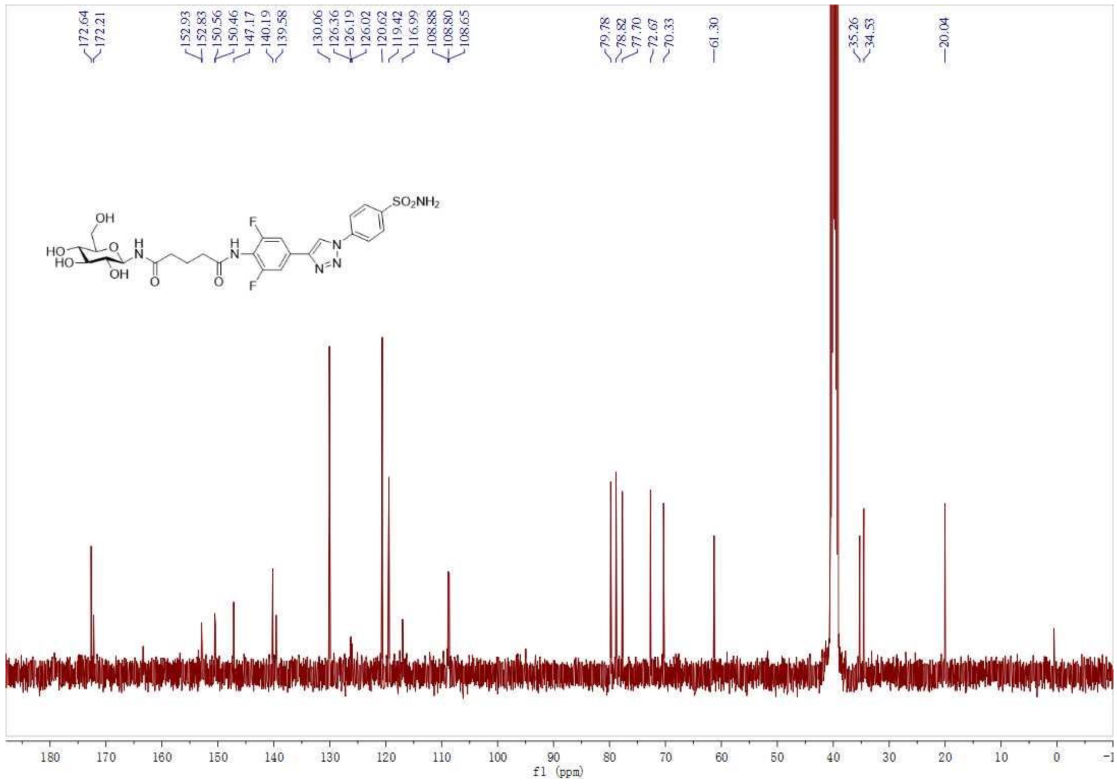

Figure S81. The ^{13}C-NMR of compound 16n

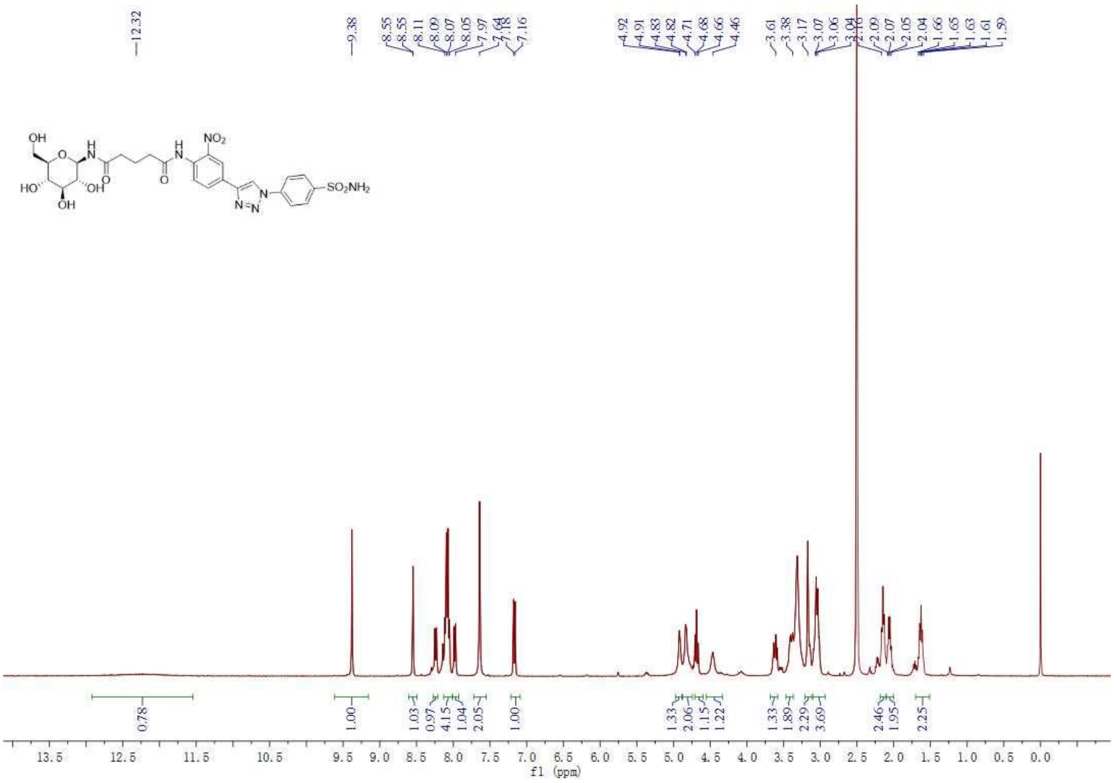

Figure S82. The ^{1}H-NMR of compound 16o

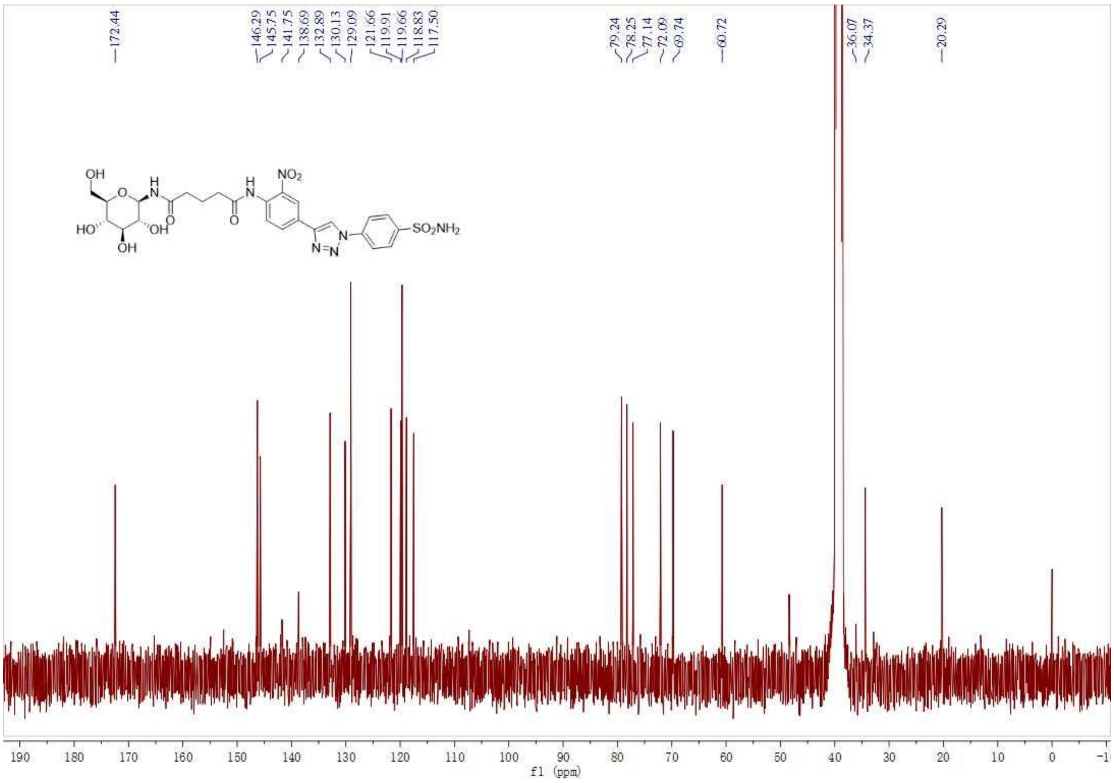

Figure S83. The ^{13}C-NMR of compound 16o
